# Evolutionary History of Sexual Differentiation Mechanism in Insects

**DOI:** 10.1093/molbev/msac145

**Published:** 2022-07-12

**Authors:** Yasuhiko Chikami, Miki Okuno, Atsushi Toyoda, Takehiko Itoh, Teruyuki Niimi

**Affiliations:** Division of Evolutionary Developmental Biology, National Institute for Basic Biology, 38 Nishigonaka, Myodaiji, Okazaki, Aichi 444-8585, Japan; Department of Basic Biology, School of Life Science, The Graduate University for Advanced Studies, SOKENDAI, 38 Nishigonaka, Myodaiji, Okazaki, Aichi, 444-8585, Japan; Division of Microbiology, Department of Infectious Medicine, School of Medicine, Kurume University, 67 Asahi-machi, Kurume, Fukuoka, 830-0011, Japan; Comparative Genomics Laboratory, National Institute of Genetics, 1111 Yata, Mishima, Shizuoka 411-8540, Japan; Advanced Genomics Center, National Institute of Genetics, 1111 Yata, Mishima, Shizuoka 411-8540, Japan; School of Life Science and Technology, Tokyo Institute of Technology, 2-12-1 Ookayama, Meguro, Tokyo 152-8550, Japan; Division of Evolutionary Developmental Biology, National Institute for Basic Biology, 38 Nishigonaka, Myodaiji, Okazaki, Aichi 444-8585, Japan; Department of Basic Biology, School of Life Science, The Graduate University for Advanced Studies, SOKENDAI, 38 Nishigonaka, Myodaiji, Okazaki, Aichi, 444-8585, Japan

**Keywords:** sexual differentiation, alternative splicing, *doublesex*, insect, Zygentoma

## Abstract

Alternative splicing underpins functional diversity in proteins and the complexity and diversity of eukaryotes. An example is the *doublesex* gene, the key transcriptional factor in arthropod sexual differentiation. *doublesex* is controlled by sex-specific splicing and promotes both male and female differentiation in holometabolan insects, whereas in hemimetabolan species, *doublesex* has sex-specific isoforms but is not required for female differentiation. How *doublesex* evolved to be essential for female development remains largely unknown. Here, we investigate ancestral states of *doublesex* using *Thermobia domestica* belonging to Zygentoma, the sister group of Pterygota, that is, winged insects. We find that, in *T. domestica*, *doublesex* expresses sex-specific isoforms but is only necessary for male differentiation of sexual morphology. This result supports the hypothesis that *doublesex* initially promoted male differentiation during insect evolution. However, *T. domestica doublesex* has a short female-specific region and upregulates the expression of *vitellogenin* homologs in females, suggesting that *doublesex* may already play some role in female morphogenesis of the common ancestor of Pterygota. Reconstruction of the ancestral sequence and prediction of protein structures show that the female-specific isoform of *doublesex* has an extended C-terminal disordered region in holometabolan insects but not in nonholometabolan species. We propose that *doublesex* acquired its function in female morphogenesis through a change in the protein motif structure rather than the emergence of the female-specific exon.

## Introduction

Animals have evolved diverse sex differences including sexual dimorphism (Darwin 1871; Geddes and Thomson 1889), which could be argued to be the driving force of eco-evolutionary dynamics, including extinction rate and interspecific interaction (Fryxell et al. 2019). In the last three decades, the genetic pathways that create sex and sexual dimorphism have been elucidated in many animal species. Although these pathways likely have a single origin (Beukeboom and Perrin 2014), they have undergone extensive changes throughout the course of animal evolution (Wilkins 1995; Bachtrog et al. 2014; Bopp et al. 2014; Herpin and Schartl 2015).

This diversity of the gene cascade can be attributed to differences in its composition. For example, in eutherians such as mice and humans, the master regulator of sex is the *Sex-determining region Y* (*Sry*), a member of the high mobility group-box transcriptional factor family (Gubbay et al. 1990; Sinclair et al. 1990; Koopman et al. 1991; Miyawaki et al. 2020), whereas *DM domain gene on the Y chromosome* (*dmy*) of the *doublesex* and *mab-3*-related transcriptional factor (DMRT) family is the master sex-determining regulator in the medaka fish (Matsuda et al. 2002; Nanda et al. 2002). Similarly, there are an increasing number of examples of diversification of the gene repertoires in animals (Hasselmann et al. 2008; Hattori et al. 2012; Sato et al. 2010; Takehana et al. 2014). In addition, recent studies on the function of *doublesex* (*dsx*) genes in Pterygota, that is, winged insects, indicate that there also seems to be diversity in sex-determining pathways that is independent of the gene composition of the cascade.

Sexually dimorphic morphology in Pterygota arises during postembryonic development. *dsx*, a member of the DMRT family, acts as a global regulator at the bottom of the cascade and thus governs sexual differentiation (Kopp 2012; Verhulst and van de Zande 2015). In many pterygote insects, *dsx* is controlled by sex-specific splicing. In Diptera, Coleoptera, Lepidoptera, and Hymenoptera, sex-specific Dsx protein variants are essential for promoting either male or female differentiation (e.g., Hildreth 1965; Burtis and Baker 1989; Ohbayashi et al. 2001; Kijimoto et al. 2012; Shukla and Palli 2012; Ito et al. 2013; Gotoh et al. 2016; Xu et al. 2017; Roth et al. 2019; Wang et al. 2022a, 2022b). For example, in the fruit fly *Drosophila melanogaster, dsx* is required for sex differentiation, as manifested in external genitalia and foreleg bristle rows, and *dsx* mutants show an intersexual phenotype in these traits, with both male and female differentiation inhibited (Hildreth and Lucchesi 1963; Hildreth 1965). In contrast, in the sawfly *Athalia rosae* (Mine et al. 2017, 2021), silverleaf whitefly *Bemisia tabaci* (Guo et al. 2018), brown planthopper *Nilaparvata lugens* (Zhuo et al. 2018), German cockroach *Blattella germanica* (Wexler et al. 2019), and damselfly *Ischnura senegalensis* (Takahashi et al. 2019, 2021), *dsx* exhibits sex-specific isoforms and is responsible for male differentiation of morphological traits, but unnecessary for female differentiation. Thus, the roles of *dsx* in sexual differentiation differ among pterygote taxa despite expressing sex-specific isoforms: the double-output, that is, essential for both male and female differentiation in holometabolan species and the single-output, that is, essential for male differentiation only in hemimetabolan and some hymenopteran species. This diversity—leading to the differences in outputs of the cascade—depends on the distinct functionality of a single gene, *dsx*, and can be attained regardless of the differences in gene composition of pathways. Therefore, it would appear that there are at least two categories of sex-determining cascades in insects: the diversity in gene repertoires and the diversity in function of a single gene, *dsx*. Currently, it remains unclear how the latter diversity evolved.

*dsx* in crustaceans and arachnids is highly expressed in males without sex-specific isoforms (Kato et al. 2011; Pomerantz et al. 2015; Li et al. 2018; Panara et al. 2019). Accordingly, *dsx* is required solely for male differentiation of morphological traits in the water flea *Daphnia magna*. Wexler et al. (2019) proposed a stepwise evolution in which *dsx* acquired sex-specific isoforms that later became essential for female differentiation. However, the role of *dsx* is more diverse than expected. In Hymenoptera, *dsx* is involved in female differentiation of gonads in the honeybee *Apis mellifera* (Roth et al. 2019) but remains nonessential for female differentiation in the sawfly *At. rosae* (Mine et al. 2017, 2021). In the milkweed bug *Oncopeltus fasciatus*, *dsx* facilitates both female and male differentiation of genital organs (Just et al. 2021). *dsx* in *Be. tabaci* positively regulates expression of a yolk precursor gene *vitellogenin* in females, but remains nonessential for female morphology (Guo et al. 2018), implying that *dsx* functionality differs in morphogenesis and other biological processes in females. Overall, inferring the evolutionary history of *dsx* in the Pterygota remains a challenging task, and it is unclear what factors led to the feminizing roles of *dsx* (Hopkins and Kopp 2021).

The phylogenetic distance between crustaceans and Pterygota and the lack of information on outgroups more closely related to Pterygota may explain current knowledge gaps in the evolution of *dsx* from a monofunctional to a bifunctional regulator in arthropods. To close this gap, we included the firebrat *Thermobia domestica* (Zygentoma) in our analysis of *dsx*. Zygentoma is the sister group of Pterygota (Misof et al. 2014), does not copulate, and displays simple sexual dimorphisms, that is, nonaedeagus male penises and female ovipositors (Kristensen 1975; Matsuda 1976; Emeljanov 2014; Beutel et al. 2017; Boudinot 2018). Otherwise, there is little morphological difference between females and males, as Darwin noted (1871: 348): “The sexes do not differ.” These features suggest that the level of sexual differentiation in this species is very simple and likely ancestral. Therefore, Zygentoma offers an ideal model for investigating the ancestral state of *dsx* in Pterygota. In this study, we investigated *dsx* in *T. domestica* and analyzed its functions in sexual differentiation. We also performed phylogenetic analysis, ancestral sequence (AS) reconstruction, and protein structure prediction to determine the evolutionary history of *dsx*.

## Results and Discussion

### Molecular Evolution of DMRT Family Genes and Gene Duplication of *dsx* in Insects

Five Doublesex and Mab-3 (DM) domain-containing genes were found in the transcriptome database of the firebrat *T. domestica*. To identify which corresponds to the *dsx* ortholog in *T. domestica*, we compared these to DM domain-containing gene homologs found in transcriptome/genome/protein databases of various arthropods and vertebrates and performed molecular phylogenetic analyses based on amino acid sequences of their DM domains.

**Fig. 1.**
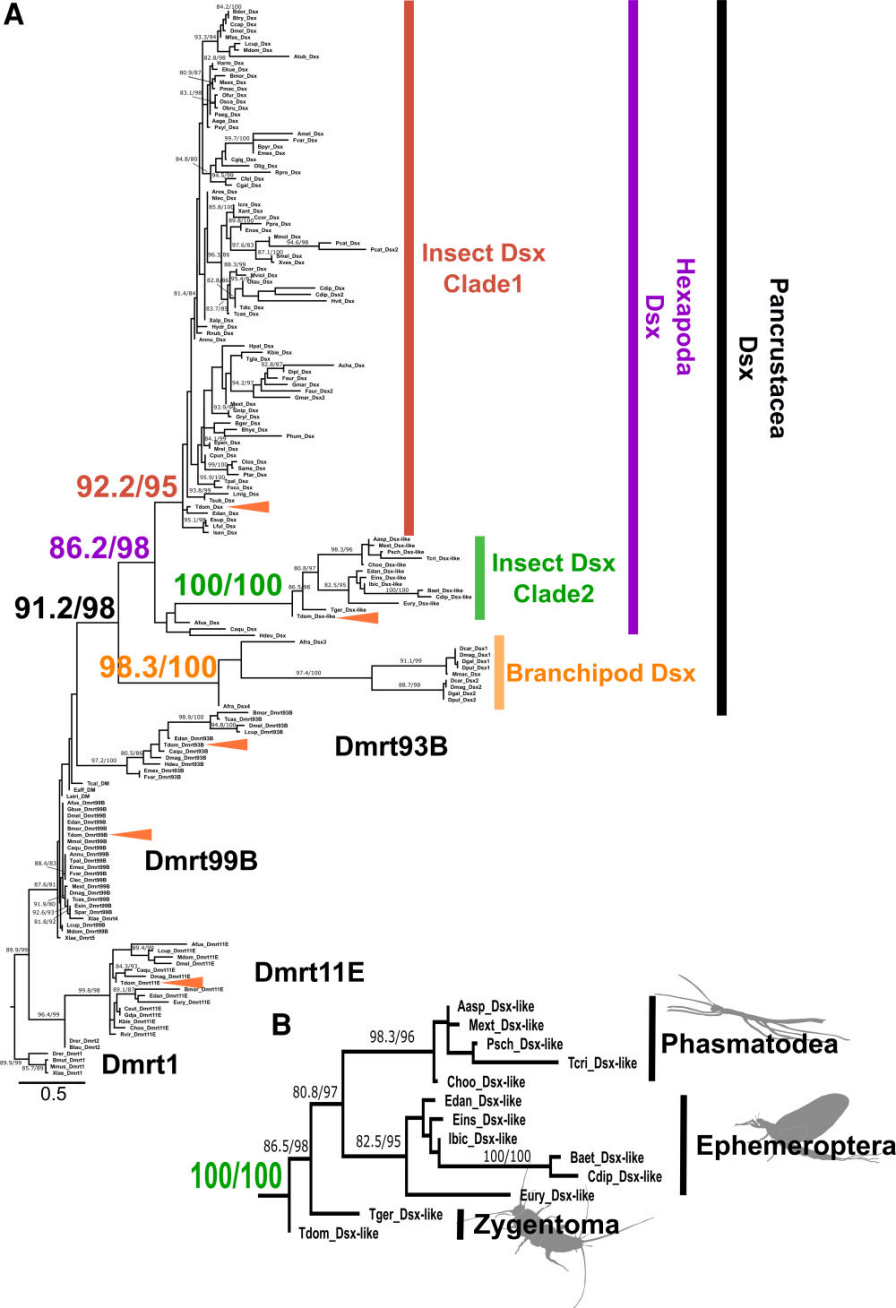
Molecular phylogeny of *doublesex* in Pancrustacea and Vertebrata. (*A*) Molecular phylogeny of *doublesex* and Mab-3-related transcriptional factors (DMRTs). Phylogenetic analysis was based on the amino acid sequences of DM domain and performed by IQ-TREE following multiple sequence alignment using MAFFT software. The maximum-likelihood method was applied. The 166 operational taxonomic units used for the phylogenetic analysis are listed in supplementary table S2, supplementary material online. Arrow heads indicate the DM domain-containing genes in *Thermobia domestica*. (*B*) Enlarged view of insect Dsx Clade2 (*dsx-like* clade). Numerical value on each node indicates SH-aLRT/UFBoot values. Values on nodes where both SH-aLRT and UFBoot are >80% are shown. Larger numbers are the supporting values on the branches relevant to this study. The full tree information can be found in a supplementary file, supplementary material online (nexus format).

We reconstructed the phylogenetic relationship of 198 DM domain-containing sequences, which covered all the hexapod orders except Archaeognatha (supplementary table S1, Supplementary Material online). However, we failed to robustly recover some clades such as Insect Dsx and Pancrustacea Dsx, because the support values, that is, SH-aLRT/UFBoot, were lower than the thresholds (supplementary fig. S1, Supplementary Material online; see Materials and Methods). We assumed that this unreliability might be due to the presence of rapidly evolving sequences. Accordingly, we excluded such problematic sequences and performed the analysis again. The new analysis covered 29 out of 32 insect orders. In the new tree, the Pancrustacean *dsx* was robustly grouped in a clade that was distinct from the other DMRT family genes (fig. 1*A*). Within this clade, three reliable subclades were recognized: Insect Dsx Clade 1, Insect Dsx Clade 2, and Branchiopod Dsx Clade. Insect Dsx Clade 1 contained the *dsx* found in Pterygota including *Drosophila melanogaster*. This clade also contained one of the five DM domain-containing gene homologs of *T. domestica*. It is likely this gene is the corresponding ortholog of *T. domestica*. In the Branchiopod Dsx Clade, there are genes from branchiopods. This clade contained two *dsx* genes previously reported in diplostracan species (Kato et al. 2011), which suggests that *dsx* was duplicated specifically in the diplostracan lineage.

We found that several species of Zygentoma, Ephemeroptera (mayflies), and Phasmatodea (stick insects) contain a *dsx-like* homolog of the Insect Dsx Clade 2 (fig. 1*A*and*B*) as well as the *dsx* ortholog of the Insect Dsx Clade 1. The two insect Dsx clades were robustly grouped into a single clade, Hexapod Dsx. Hence, it is inferred that the duplication of *dsx* occurred from the common ancestor of Branchiopoda and Hexapoda before Zygentoma diverged. Also, the two paralogs may have been retained until at least the divergence of Eumetabola (=Hemiptera + Thysanoptera + Psocodea + Holometabola). The molecular evolution of *dsx* has been inferred from *dsx* for some pterygote insects, mainly holometabolan insects (Wexler et al. 2014; Mawaribuchi et al. 2019), and the presence of a *dsx-like* gene may have been overlooked in their analyses.

Dsx has an intertwined Zinc Finger region within the DM domain that is important for the roles of Dsx (Zhu et al. 2000). This region comprises two motifs, CCHC and HCCC. To investigate whether there is an intertwined structure in *dsx-like*, we compared the sequences of Dsx and Dsx-like of *T. domestica*. We found both the CCHC and HCCC motifs in Dsx and Dsx-like (supplementary fig. S2, Supplementary Material online). Therefore, *dsx-like* is an active gene rather than a pseudogene in terms of the functional domain structure.

In summary, we report that the genome of *T. domestica* also contains both *dsx* and *dsx-like*, reflecting the presumed ancestral state in Pterygota in terms of the gene copy number of *dsx*. Gene duplication generally leads to neo-/sub-functionalization, which is a precursor to functional diversification (cf., Taylor and Raes 2004). Our results include an analysis of the expression profiles and functions of *dsx* as well as *dsx-like* in *T. domestica*.

### Sex-specific Splicing of *dsx* in *T. domestica*

**Fig. 2.**
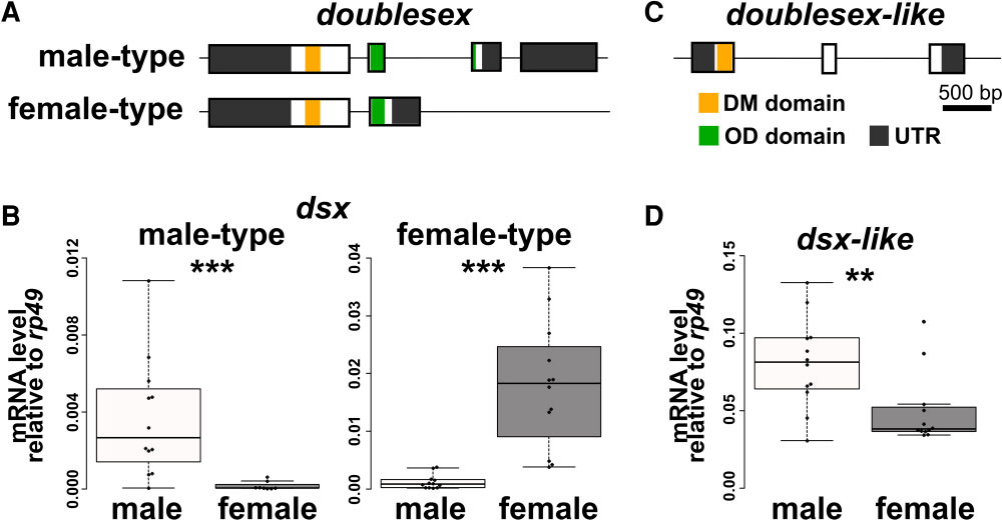
Structural features of *dsx* and *dsx-like* in *Thermobia domestica*. (*A*) Exon–intron structures of *dsx* in *Thermobia domestica*. Upper and lower schematic images show the gene structure of *dsx* male-type and female-type, respectively. (*B*) Expression level of *dsx* in males and females of *T. domestica*. (*C*) Exon–intron structures of *dsx-like* in *T. domestica*. (*D*) Expression level of *dsx-like* in males and females. Exon–intron structure is determined by mapping the mRNA sequence of each gene to the genome of *T. domestica*. Expression level (*B* and *D*) was measured using RT-qPCR of *dsx* and *dsx-like* in the adult fat body and is indicated as relative values to expression of the reference gene, *ribosomal protein 49* (*rp49*). Each plot signifies the mRNA expression level of each individual. Total *N* = 20 (*dsx* male-type), 23 (*dsx* female-type), and 24 (*dsx-like*). Results of Brunner–Munzel tests are indicated by asterisks: **P < 0.01; ***P < 0.001 and are described in supplementary table S3, supplementary material online.

Splicing of *dsx* produces sex-specific isoforms in all pterygote insects studied thus far, with the exception of the termite *Reticulitermes speratus* (Miyazaki et al. 2021), the silverleaf whitefly *Be. tabaci* (Guo et al. 2018), and the body louse *Pediculus humanus* (Wexler et al. 2019), suggesting that sex-specific splicing regulation of *dsx* was acquired before the divergence of Pterygota. To examine whether the sex-specific splicing of *dsx* occurred before the emergence of pterygote insects, we investigated the expression profile of *dsx* and *dsx-like* in *T. domestica*. Full-length mRNA sequences of *dsx-like* and *dsx* in *T. domestica* were determined using RNA-seq and rapid amplification of cDNA ends methods. We then investigated gene structures by mapping the mRNA sequences to our genome database. *dsx* consists of five exons with two isoforms (fig. 2*A*): a long (951 bp) and short (756 bp) isoform. The result of reverse transcriptional quantitative polymerase chain reaction (RT-qPCR) analysis showed that long and short isoforms were highly expressed in males and females, respectively (fig. 2*B*; Brunner–Munzel test, *P* = 1.75 × 10^−6^ and 2.20 × 10^−16^ in the long and the short isoforms). This result suggests *dsx* is controlled by sex-specific splicing. We refer to the male-biased isoform as *dsx* male-type and the female-biased isoform as *dsx* female-type. The *dsx-like* is expressed approximately 2-fold more in males than females (Brunner–Munzel test, *P* = 0.00924) and has three exons but no sex-specific isoform (fig. 2*C*and*D*), indicating that *dsx-like* is not regulated by sex-specific splicing.

Our results provide further evidence that sex-specific splicing of *dsx* already existed in the common ancestor of Pterygota and Zygentoma (= Dicondylia), which diverged ∼421 Ma. Misof et al. (2014) estimated that the common ancestor of *Daphnia* and hexapods occurred at ∼508 Ma. Therefore, *dsx* sex-specific splicing regulation is a plesiomorphic feature of insects acquired between 508 and 421 Ma and has been conserved for ∼400 My in each taxon of Dicondylia.

### Function of *dsx* for Internal Reproductive System and Body Size in *T. domestica*

Deciphering the role of *dsx* of *T. domestica* is essential for determining the ancestral roles of *dsx* in pterygote insects. Hence, we conducted a functional analysis of not only *dsx* but also *dsx-like*, given that this paralog may also play a role in sexual differentiation.

We silenced *dsx* and *dsx-like* by RNA interference (RNAi) and then quantified the expression of *dsx* and *dsx-like* in fat bodies of RNAi individuals by RT-qPCR. *dsx* silencing in females and *dsx-like* silencing in both sexes showed significantly reduced expression of each target genes compared with their expression in *enhanced green fluorescent protein* (*egfp*) RNAi controls (Brunner–Munzel test, *P* = 0.0265 in female *dsx*, 4.40 × 10^−16^ in male and female *dsx-like*; supplementary fig. S3*A*and table S3, Supplementary Material online). In the case of *dsx* RNAi males, we did not find any significant effect related to *dsx* expression. Suspecting that outliers might have affected this result, we tested for outliers in *dsx* RNAi males and found one outlier (supplementary table S4, Supplementary Material online). Our reanalysis, after having removed the outlier, showed that *dsx* expression was significantly reduced in *dsx* RNAi males (Brunner–Munzel test, *P* = 0.00545; supplementary fig. S3*B* and table S3, Supplementary Material online). Therefore, we concluded that *dsx* and *dsx-like* dsRNAs can knock down each target gene. Finally, we report that *dsx* RNAi showed no effect on *dsx-like* expression and vice versa.

**Fig. 3.**
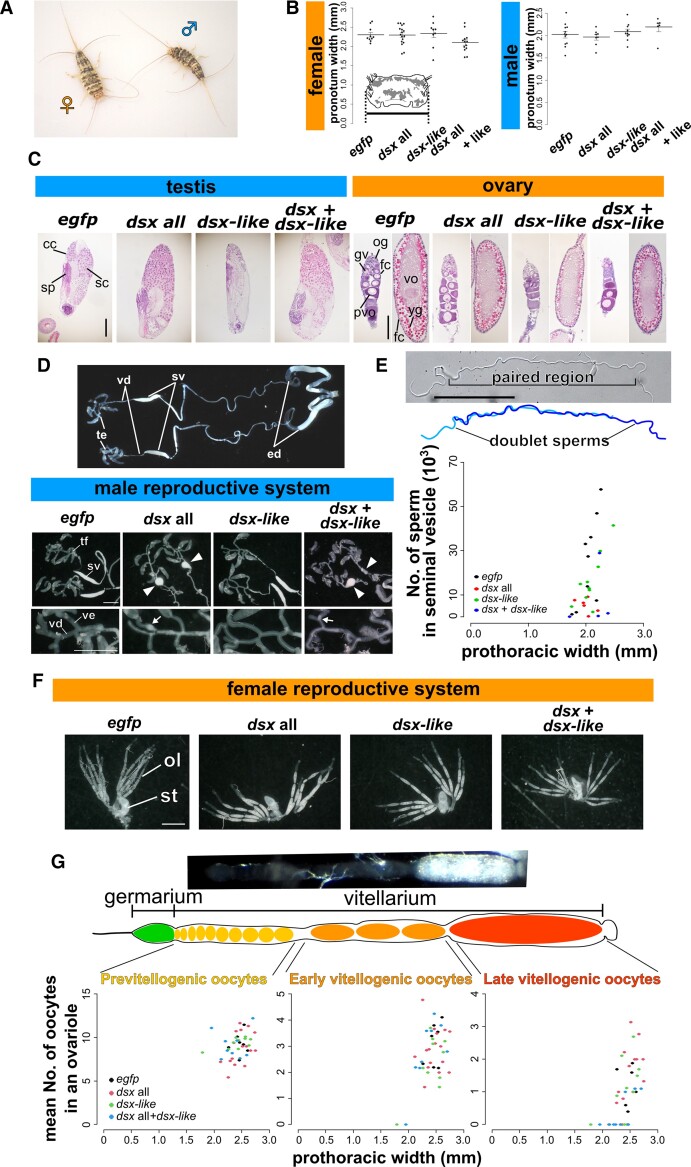
Function of *doublesex* and *doublesex-like* in terms of body size, internal reproductive system, and gametogenesis of *Thermobia domestica*. (*A*) A pair of *T. domestica*. Female and male are somewhat similar in appearance. (*B*) Body size of RNAi treatment groups. Width of the pronotum (prothoracic tergum) was used as an indicator of body size. Graph shows mean ± SE (standard error). Results of the GLM analysis shown in supplementary table S5 (female) and S6 (male), supplementary material online. Any significant effect can be detected in the RNAi treatments. Total *N* = 49 in females and 36 in males. (*C*) Histology of gonads in the RNAi groups. Paraffin. Hematoxylin–Eosin staining. For ovary images, left and right panels in each treatment show germarium/previtellogenesis and vitellogenesis, respectively. (*D*) Effects of RNAi on male internal reproductive system. Upper photo shows the gross morphology of the reproductive systems in the nontreated male. Lower photos show the morphology of RNAi males (arrowheads show rounded seminal vesicle). Lowest photos are focused on the vas efferens (arrows show sperm clogged in the tubule). (*E*) Sperm of RNAi males. Upper photo and figure are sperm morphology in the nontreated male. The sperm forms doublets in the seminal vesicle. Lower figure shows sperm number of RNAi males. Results of the GLM analysis are shown in supplementary table S6, supplementary material online. Significant effect was detected in the *dsx* RNAi treatment (*P* = 0.00487). Total *N* = 29. (*F*) Effects of RNAi on the female internal reproductive system. (*G*) Effects of the RNAi on oocyte number. Upper photo shows the ovariole of the nontreated female. Lower figures show the number of oocytes in RNAi females along with oogenetic stages. Results of the GLM analysis are given in supplementary table S5, supplementary material online. The number of late vitellogenic oocytes was correlated with pronotum width, although any significant effect can be detected in RNAi treatments. Total *N* = 42 in each stage. In each panel, the *egfp*, *dsx* all, *dsx-lik*e and *dsx* + *dsx-like* represent the *egfp* dsRNA injected group (control), *dsx* sex-common region dsRNA injected group, *dsx-like* dsRNA injected group, and both *dsx* sex-common region and *dsx-like* dsRNAs injected group, respectively. Each plot in (*B*), (*E*), and (*G*) represent the value of each individual. cc, cystocyte; fc, follicle cell; gv, germinal vesicle; og, oogonia; ol, ovariole; pvo, previtellogenic oocyte; sc, spermatocyte; sp, sperm; st, spermatheca; sv, seminal vesicle; tf, testicular follicle; yg, yolk granule; ve, vas efferens; vd, vas deferens, vo, vitellogenic oocyte. Scales: 50 µm (*C*); 10 µm (*E*); 1,000 µm (*D* and *F*).

We also performed a double knockdown of *dsx* and *dsx-like*, to address the possibility that *dsx* and *dsx-like* are redundant. We examined the effects of silencing on sexual dimorphism, specifically body size (fig. 3*A*) and development of reproductive systems. The pronotum width as a measure of body size was not affected in either knockdown group (fig. 3*B*; supplementary tables S5 and S6, Supplementary Material online). The *dsx* RNAi, *dsx-like* RNAi, and double knockdown did not show any histological differences in testes, ovaries, and gametogenesis compared with the controls (fig. 3*C*; Supplementary material S1, Supplementary Material online). Conversely, in the *dsx* knockdown group (*dsx* alone or *dsx* and *dsx-like*), the male seminal vesicle, which is a sperm storage organ and normally has a bean pod shape, became rounded (fig. 3*D*).

Given the morphological abnormality in the seminal vesicle of *dsx* RNAi males, the storage sperm might have been affected. Therefore, we calculated the number of sperm in the seminal vesicle and found that the number in *dsx* RNAi males was lower than in the control group (fig. 3*E*; supplementary table S6, Supplementary Material online; generalized linear model [GLM], *P* = 0.00487). Silencing *dsx* or *dsx-like* or both did not affect normal differentiation of the female reproductive systems, including the spermatheca (fig. 3*F*; supplementary fig. S4, Supplementary Material online). Furthermore, there was no effect on the number of oocytes in any treatment (fig. 3*G*; supplementary table S5, Supplementary Material online).

The lack of effect of RNAi on the development of testes and ovaries may be due to the timing of the RNAi treatment, which was performed after gonadal differentiation. This is supported by the findings of previous study (Klag 1977) suggesting that sex differences in gonads and germ cells arise during embryogenesis. Analysis of embryonic RNAi would be necessary to test this hypothesis, and such experiment will be left to future studies, as our study focused on *dsx* function during postembryonic development. The impact of *dsx* RNAi on the storage sperm number would seem to depend on the abnormality of the seminal vesicle, given that we did not detect effects on spermatogenesis in *dsx* RNAi males. The lack of effect of *dsx* on the body size of *T. domestica* is consistent with studies in *D. melanogaster* (Hildreth 1965; Rideout et al. 2015). The effect on the development of internal reproductive systems other than the gonads is consistent with results from studies of *At. rosae* (Mine et al. 2017, 2021) and *Bl. germanica* (Wexler et al. 2019).

### Function of *dsx* in the Morphology of Genital Organs of *T. domestica* and Evolution of the Function of *dsx* in Sexual Morphogenesis in Insects

**Fig. 4.**
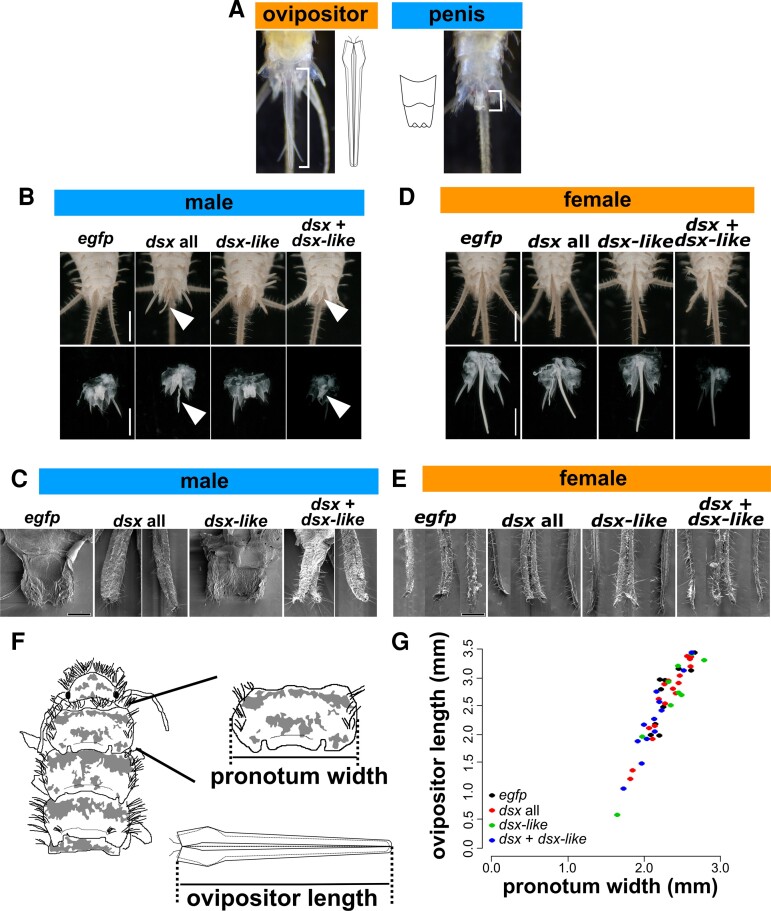
Function of *doublesex* and *doublesex-like* in the morphogenesis of genital organs in *Thermobia domestica*. (*A*) Sexually dimorphic traits of *T. domestica*: females possessing an ovipositor and males a penis. (*B*) Effects of RNAi treatments on male penial structure. Upper image: ventral side of male abdomen. Lower images focus on male penis. Arrowheads indicate ovipositor-like structure in *dsx* or both *dsx* and *dsx-like* RNAi groups. (*C*) SEM images of male penial structure. In *dsx* and *dsx* + *dsx-like* RNAi, the two photos are merged into one image. Left panel: ovipositor valvula II (inner sheath)-like structure. Right panel: ovipositor valvula I (outer sheath)-like structure. Details can be found in Supplementary material online. (*D*) Effects of RNAi treatments on female ovipositor. Upper images: ventral side of the female abdomen. Lower images: female ovipositor. (*E*) SEM images of female ovipositor structure. Left and right panels show the valvula II; middle panel shows the valvula I. The results of histological observations are shown in supplementary fig. S3, supplementary material online. Details can be found in Supplementary material online. (*F*) Schematic images of measured parts. (*G*) Effects of RNAi treatments on growth of ovipositor. Each plot indicates ovipositor length of each individual. Results of the GLM analysis are shown in supplementary table S5, supplementary material online. Ovipositor length correlated with prothoracic width (*P* = 2.00×10^−16^), although any significant effects can be seen in RNAi treatments. Total *N* = 38. In each panel, the *egfp*, *dsx* all, *dsx-lik*e, and *dsx* + *dsx-like* indicates *egfp* dsRNA injected group (control), *dsx* sex-common region dsRNA injected group, *dsx-like* dsRNA injected group, and both *dsx* sex-common region and *dsx-like* dsRNAs injected group, respectively. Scales: 1 cm (B and D); 50 µm (*C* and *E*).

In *T. domestica*, sexual dimorphism is evident in the external genital organs, that is, male penis and female ovipositor (fig. 4*A*). Males of *T. domestica* have unpaired small external genitalia on the abdominal segment IX. Females have an ovipositor consisting of two paired appendage-like structures on the abdominal segments VIII and IX. Males of the *dsx* knockdown groups (*dsx* only and both *dsx* and *dsx-like* RNAi) had two pairs of appendage-like structures resembling the female ovipositor (fig. 4*B*and*C*; supplementary material, Supplementary Material online); this effect was not observed in *dsx-like* RNAi males. Our results indicate that *dsx* is essential for male differentiation of morphological traits in *T. domestica*. Our analysis showed that—in contrast to males—females were not affected by the RNAi treatments, neither in terms of the external morphology (ovipositors) or at the tissue or cellular levels (fig. 4*D*and*E*; supplementary fig. S5, Supplementary Material online). We then measured the length of the ovipositor in females of the RNAi-treated groups to examine the involvement of *dsx* and *dsx-like* in growth of female morphology. The results show that *dsx* and *dsx-like* RNAi had no significant effect on ovipositor length (fig. 4*F*and*G*; supplementary table S5, Supplementary Material online).

One possible reason why *dsx* RNAi failed to affect female morphology is that *dsx* is not essential for female morphogenesis. Alternatively, the knockdown level of *dsx* mRNA could have been insufficient in females. Indeed, the remaining mRNA level of *dsx* RNAi groups relative to the controls was ∼30% in males and ∼50% in females at the median (supplementary table S3, Supplementary Material online). Therefore, the knockdown efficiency of *dsx* was less in females than in males in terms of the median. However, the mRNA levels of individual females showed that, in half of the females, the level of expression of *dsx* was lower than the minimum value of the control group (supplementary fig. S3*A*, Supplementary Material online). In some female individuals, *dsx* mRNA was repressed to levels that were the same or lower as the corresponding levels in males with morphological defects. Hence, we concluded that *dsx* expression is suppressed in a certain number of females used in each analysis. Because we could not find any effect on female morphology even in females where *dsx* is severely suppressed, we surmise that the insufficient knockdown level of *dsx* is unlikely to be the reason for the lack of the impact of *dsx* RNAi on female morphology. Plausibly, *dsx* would be nonessential for female differentiation of morphology during postembryonic development in *T. domestica*.

The knockdown of both *dsx* and *dsx-like* had the same effect as *dsx* RNAi alone. It is likely that, because *dsx-like* is not involved in the development of sexual morphology in *T. domestica*, we could not detect differences in the phenotype of the male genital organ between the double knockdown of *dsx* and *dsx-like* and the single knockdown of *dsx*. Also, it is unlikely that *dsx-like* functions redundantly with *dsx*.

Sexual morphology, for example, reproductive systems and genital organs, formed during postembryonic development is controlled by *dsx* in males but is *dsx*-independent in females of nonholometabolan insects such as *T. domestica* (Zygentoma: this study), *Bl. germanica* (Dictyoptera: Wexler et al. 2019), and the brown planthopper *Ni. lugens* (Hemiptera: Zhuo et al. 2018). Based on these findings, we infer that *dsx* may not be essential for female differentiation of morphology at the evolutionary point of the common ancestor of Dicondylia, strongly supporting the hypothesis proposed by Wexler et al. (2019).

To elucidate the timing of acquisition of roles of *dsx* in female morphogenesis during postembryonic development, we must interpret the role of *dsx* in Hymenoptera, the basal clade of Holometabola. Studies on the honeybee, *Ap. mellifera*, have revealed through genome editing that *dsx* controls female differentiation of the internal reproductive system under worker nutrition conditions (Roth et al. 2019). In the honeybee, sex differences in the gonads are established during embryogenesis (Lago et al. 2020). Therefore, the male-like reproductive organ in *dsx* mutant females in Roth et al. (2019) would be expected to show an effect during embryogenesis, not during postembryonic development. We cannot conclude whether *dsx* is nonessential for female morphogenesis in the honeybee, in as much as the information on the roles of *dsx* in sexual morphology is limited to the gonads and heads of worker females. However, given that *dsx* does not affect the development of the head in *Ap. mellifera* females (Roth et al. 2019), leg pigmentation, pheromone synthesis, and wing morphology in females of the parasitoid wasp *Nasonia vitripennis* (Wang, Rensink, et al. 2022; Wang, Sun, et al. 2022), and female traits in *At. rosae* (Mine et al. 2017, 2021), it is reasonable to infer for the time being that *dsx* is nonessential for female morphogenesis during postembryonic development in the common ancestor of Hymenoptera. This interpretation and the essential roles of *dsx* for female development that have been uncovered in other holometabolan insects suggest that *dsx* became essential for feminization of morphology during postembryonic development at the point of the common ancestor of holometabolan insects except for Hymenoptera, that is, Aparaglossata, emerging ∼327 Ma.

### Cryptic Role of *doublesex* for Female-Specific Transcripts in *T. domestica* and its Opposite Roles in the Sexes

*dsx* in *T. domestica* does not seem to show opposing functions between sexes in postembryonic morphogenesis. Conversely, other biological processes are open to further investigation. We tested whether *dsx* contributes to expression of *vitellogenin* (*vtg*), a yolk protein precursor gene that is highly expressed in female animals (Byrne et al. 1989; Hayward et al. 2010). Previous studies have shown that *vtg* in pterygote insects is controlled by *dsx* (e.g., Suzuki et al. 2003; Shukla and Palli 2012; Thongsaiklaing et al. 2018). Our RNA-seq analysis showed that three *vtg* homologs, that is, *vtg1*, *vtg2*, and *vtg3,* expressed female-specificity in the fat body in *T. domestica* (supplementary fig. S6 and table S7, Supplementary Material online). Through the use of RT-qPCR, we analyzed the expression of *vtg* in fat bodies of *dsx, dsx-like,* or both genes RNAi groups.

**Fig. 5.**
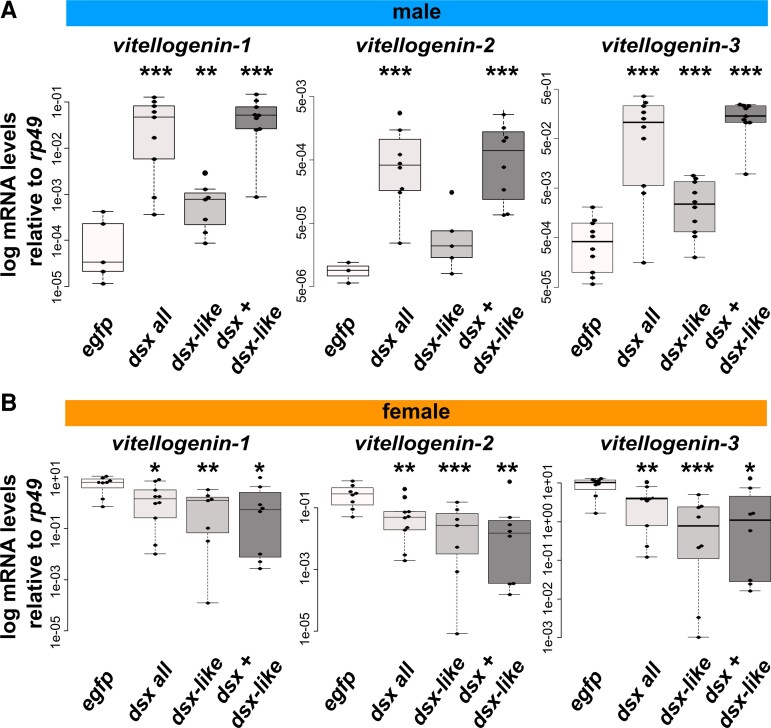
Function of *doublesex* for *vitellogenin* expression in *Thermobia domestica*. (*A*) *Vitellogenin* expression level in RNAi males. (*B*) *vitellogenin* expression level in RNAi females. mRNA expression levels were measured through RT-qPCR. Shown are the log-scale relative values of expression levels of three *vitellogenin* homologs to the reference gene, *ribosomal protein 49* (*rp49*). Each plot indicates mRNA expression levels of each individual. In each panel, *egfp*, *dsx* all, *dsx-lik*e, and *dsx* + *dsx-like* indicates *egfp* dsRNA injected group (control), *dsx* sex-common region dsRNA injected group, *dsx-like* dsRNA injected group, and both *dsx* sex-common region and *dsx-like* dsRNA injected group, respectively. Brunner–Munzel test was performed to ascertain the difference in mRNA expression level between control and *dsx* or *dsx-like* RNAi groups. *P*-values were adjusted with Holm’s method. *P < 0.05, **P < 0.001, ***P < 0.0001. *P* ≥ 0.05 is not shown. Statistical results are described in supplementary table S3, supplementary material online. Total *N* = 30 (*vitellogenin-1*), 24 (*vitellogenin-2*) and 39 (*vitellogenin-3*) in males and 33 (*vitellogenin-1*), 33 (*vitellogenin-2*), and 34 (*vitellogenin-3*) in females.

In *dsx* RNAi males, all *vtg* mRNAs were expressed 45- to 1,530-fold higher than controls (fig. 5*A*; supplementary table S3, Supplementary Material online: Brunner–Munzel test, *P* = 2.87 × 10^−8^, 6.60 × 10^−16^ and 2.80 × 10^−4^ in *vtg1*, *vtg2*, and *vtg3*). *vtg1* and *vtg3* mRNAs were significantly up-regulated in *dsx-like* RNAi males compared with controls (fig. 5*A*: Brunner–Munzel test, *P* = 0.0139 and 0.00497 in *vtg1* and *vtg3*). In both *dsx* and *dsx-like* RNAi males, the effect was similar to that in *dsx* RNAi males (fig. 5*A*: Brunner–Munzel test, *P* = 6.60 × 10^−16^, 0.0162, and 6.60 × 10^−16^ in *vtg1*, *vtg2*, and *vtg3*). We then found that the expression of all *vtg* genes was significantly reduced in *dsx* RNAi females (fig. 5*B*; supplementary table S3, Supplementary Material online: Brunner–Munzel test, *P* = 0.0433, 0.00422, and 0.00623 in *vtg1*, *vtg2*, and *vtg3*). This reduction rate was approximately 0.2- to 0.4-fold. Furthermore, *vtg* expression was significantly reduced in *dsx-like* RNAi females (Brunner–Munzel test, *P* = 0.00256, 3.80 × 10^−6^, and 1.49 × 10^−5^ in *vtg1*, *vtg2*, and *vtg3*) and both *dsx* and *dsx-like* RNAi females (fig. 5*B*; Brunner–Munzel test, *P* = 0.0305, 0.00892, and 0.0197 in *vtg1*, *vtg2*, and *vtg3*). These results show that *dsx* and *dsx-like* of *T. domestica* control *vtg* negatively in males and positively in females.

Our results indicate that *dsx* performs opposite roles in the sexes, that is, repressive in males and promotive in females in terms of *vtg* expression. *dsx-like* also shows opposite functions for *vtg* expression in males and females. It is unlikely that this result is due to *dsx-like* regulating *dsx* transcription, as *dsx-like* did not affect *dsx* expression (supplementary fig. S3*A*, Supplementary Material online). A possible hypothesis is that *dsx-like* may regulate *vtg* expression as a co-regulator that binds *dsx* or other transcription factors.

We do not know whether *dsx* of *T. domestica* has sex-opposite control over genes other than *vtg* homologs, as much as our analysis was limited to *vtg* homologs. However, our analysis of these genes indicates that molecular function of *dsx* in this species includes sex-opposite function in the transcription of some genes. In *Be. tabaci*, *dsx* positively regulates *vtg* expression in females, even though it is nonessential for female differentiation of morphological traits (Guo et al. 2018). *dsx* of this species does not negatively regulate *vtg* in males. Therefore, the functionality of *dsx* found in *T. domestica*, in other words, the sex-opposing roles in the expression of some genes and functions that are nonessential for female morphogenesis, is a phenomenon that is yet to be reported in any insect or other animal. This functionality indicates that even if *dsx* can oppositely function in a sex-opposite sense for some gene expression, it does not necessarily show opposing functions in morphogenesis between sexes. This difference in functionality may be due to the differences in genes under *dsx* control, some influencing morphogenesis and others different aspects such as yolk synthesis in females.

Genes under *dsx* control in males are *dsx*-free in females of *I. senegalensis* (Takahashi et al. 2021), *Bl. germanica* (Wexler et al. 2019; Pei et al. 2021), and *Ni. lugens* (Zhuo et al. 2018). Feminizing roles of *dsx* in morphogenesis and other biological processes may have appeared in the common ancestor of Aparaglossata (or Holometabola) as an entirely novel function, that is, neo-functionalization. In contrast, the contribution of *dsx* to the expression of some genes in females of *T. domestica* (this study), *Be. tabaci* (Guo et al. 2018), *Ap. mellifera* (Velasque et al. 2018), and Aparaglossata raises the alternative hypothesis that the capacity of *dsx* to contribute to female differentiation was already present in the common ancestor of Dicondylia and later became essential for female morphogenesis in the common ancestor of Aparaglossata. In this evolutionary process, the role of *dsx* in feminization of postembryonic morphogenesis in Aparaglossata could be due to extending its capability to control some genes in females. Moreover, the capacity to regulate some female genes might be a “minor function” of *dsx* in nonholometabolan females, as predicted by Wexler et al. (2019).

At this time, we do not have sufficient evidence to determine which of these hypotheses is the most plausible. However, the latter hypothesis may plausibly explain the presence of female-specific coding sequences of *dsx* and the high expression of *dsx* female-type during postembryonic development, in non-Aparaglossatan insects. In general, there seems to be a low expression level in nonfunctional isoforms (Keren et al. 2010). Furthermore, neutral or nonfunctional isoforms are rapidly replaced across species and sometimes lost or become nonsense forms with very short cording regions, suggesting that isoforms and exons need to have some function to be maintained across species (Graveley 2001; Xing and Lee 2006; Kelemen et al. 2013). It is therefore possible that, due to roles such as regulating female *vitellogenin* transcription, *dsx* could maintain female isoforms and exons with long coding regions across taxa and be highly expressed during postembryonic development.

### Evolution of C-terminus Disordered Region of *dsx* Female-type

**Fig. 6.**
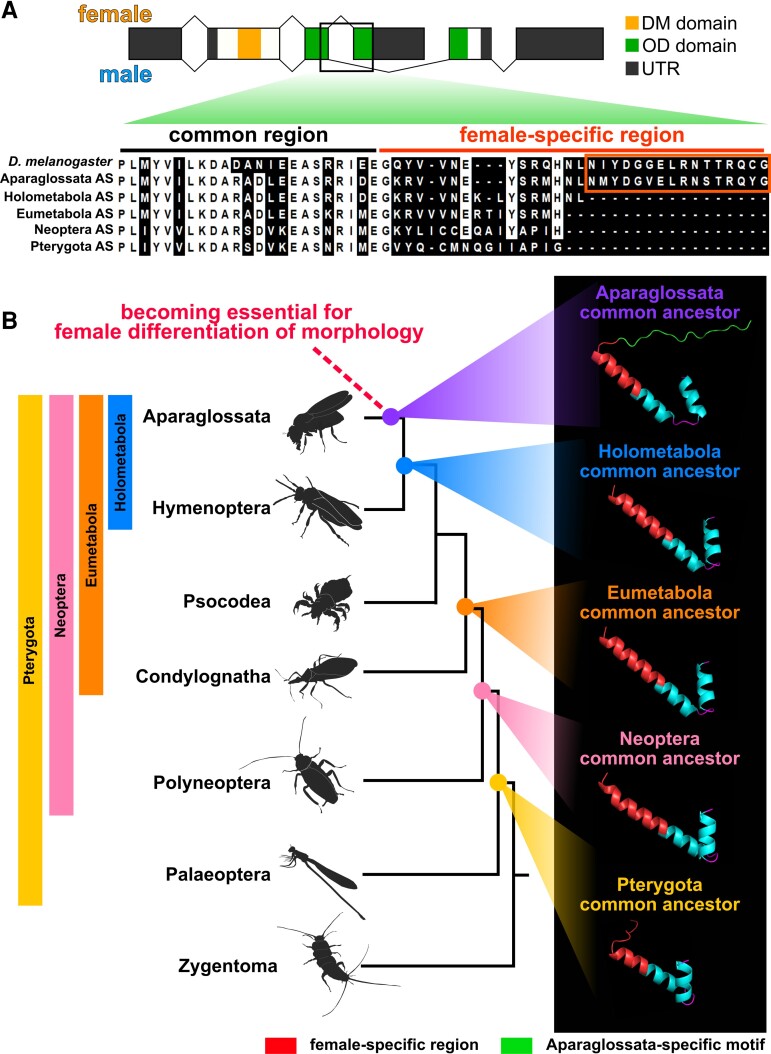
Evolution of C-terminal sequence of *doublesex* in insects. (*A*) ASs of *dsx*. AS were reconstructed from 49 *dsx* proteins of insects via maximum-likelihood methods of MEGA X. Details on species and proteins used for the AS reconstruction are found in supplementary table S8, supplementary material online. The most probable sequences were applied. Results of AS reconstruction are described in supplementary table S9, supplementary material online. Probabilities of sites of AS are listed in supplementary table S11, supplementary material online. All sites of Aparaglossata-specific region in AS other than Aparaglossata AS were gaps with probabilities >0.9. Details are given in tsection Materials and Methods. Upper scheme: *dsx* gene structure of *D. melanogaster*. Lower image: outcome of multiple sequence alignments (MSA) of *dsx* sequences by MAFFT. OD domain sequences at C-terminal side were used for MSA. White background in MSA result indicates the conserved sites that share residues in the 80% taxa. The Aparaglossata-specific motif is indicated by orange frame. (*B*) Predicted protein structures of *dsx* female-type in common ancestors of insect taxa. The phylogenetic relationship is based on topology from Misof et al. (2014). 3D images in the right panel indicate predicted structures of the OD domain including female-specific region of *dsx*. Protein structures were predicted using AlphaFold2-based algorism (ColabFold: Mirdita et al. 2021). Red in the 3D image indicates female-specific region, green indicates Aparaglossata-specific motif. Information on the reliable values, that is, predicted local distance difference test: plDDT, of the prediction is given in the Material and Methods section and supplementary fig. S11, supplementary material online.

One of the most puzzling problems is how *dsx* became recruited, in an evolutionary sense, for female differentiation of morphological traits (cf., Hopkins and Kopp 2021). Here, we found that the C-terminal sequences, including the oligomerization (OD) domain of the Dsx female-type, is much shorter in *T. domestica* (38 aa) than in *D. melanogaster* (53 aa) (supplementary fig. S7, Supplementary Material online). The OD domain is essential for female differentiation in *D. melanogaster* as it physically binds to Dsx itself, transcription factors, and co-activators (An and Wensink 1995; Erdman et al. 1996; Ghosh et al. 2019; Romero-Pozuelo et al. 2019). Therefore, we hypothesized that the additive region found in *D. melanogaster* occurred at the evolutionary stage of the common ancestor of Aparaglossata, at which point *dsx* became essential for female morphogenesis. To test this hypothesis, we obtained sequences of Dsx female-type from 48 insect species based on the National Center for Biotechnology Information protein/transcriptome shotgun assembly database and previous studies (supplementary table S8, Supplementary Material online) and reconstructed ASs of the Dsx female-type. Reconstruction of the ASs revealed that the C-terminal 16-amino acid region of Dsx female-type found in the common ancestor of Aparaglossata was absent in common ancestors of other taxa (fig. 6*A*; supplementary fig. S8 and table S9, Supplementary Material online). This motif was conserved within Aparaglossata in our dataset although moderate sequence diversification was observed (supplementary fig. S8, Supplementary Material online). In our dataset, almost none of the sequences of this motif were found in species in which *dsx* is nonessential for female differentiation of morphological traits during postembryonic development. Exceptionally, *dsx* of *At. rosae* showed an amino acid sequence in the region corresponding to this motif. However, most of the sequences widely conserved in Aparaglossata were not seen in *At. rosae* (supplementary fig. S8, Supplementary Material online). Furthermore, our reconstruction of the ASs revealed that the relevant regions were acquired independently in *At. rosae* and Aparaglossata (fig. 6*A*).

The Aparaglossata-specific region is located in the distal (C-terminal) part of the female-specific region in *D. melanogaster*. This distal region is a disordered region, that is, a mobile region that lacks a fixed structure, following an α-helix loop in the proximal region (Yang et al. 2008). To investigate whether the acquisition of the disordered region occurred in Aparaglossata, we predicted the protein structure of Dsx female-type ASs of Pterygota, Neoptera, Eumetabola, Holometabola, and Aparaglossata. According to the Alphafold2 algorism-based structure prediction, the female-specific region of Dsx in the common ancestor of Aparaglossata had a proximal α-helix loop structure and a distal random coil indicating a disordered region (fig. 6*B*). This structure is similar to that of *D. melanogaster*, as determined through a crystal structural analysis (Yang et al. 2008). The proximal α-helix loop structure also predicted the common ancestors of taxa other than Aparaglossata. The random coil following the α-helix structure was predicted in all common ancestors, but its length was shorter than that of the common ancestor to Aparaglossata. Determining the structure via nuclear magnetic resonance or cryo-electron microscopy will help verify essential details at the structural level; however, our theoretical predictions suggest that the disordered region following the α-helix structure in the female-specific region may have been extended in the common ancestor of Aparaglossata.

Our results suggest that both extension of the disordered region following the α-helix loop in the female-specific region of Dsx and the feminizing function of *dsx* in morphogenesis occurred in the common ancestor of Aparaglossata. Currently, the causality between these two events is uncertain, as we do not know which event occurred first. In general, disordered regions in transcription factors play essential roles in transcriptional activity through post-translational modifications and binding to co-activators and nucleic acids (Liu et al. 2006; Darling and Uversky 2018). Furthermore, Wang et al. (2019) have shown that in the diamondback moth *Plutella xylostella,* when the Aparaglossata-specific motif is specifically broken by deletion or frameshift mutations using the CRISPR/Cas9 method, the female morphology is transformed into the intersexual phenotype. This result indicates that the Aparaglossata-specific motif is essential for female differentiation of morphology in *Pl. xylostella*. These facts suggest that extension of the C-terminal region of Dsx female-type may have been a key event associated with acquiring the female-differentiating roles of *dsx* in morphology during postembryonic development.

The general predictions are that low adaptive isoforms will theoretically become more adaptive through later accumulation of mutations (Boue et al. 2003; Xing and Lee 2006; Keren et al. 2010). In these predictions, such isoforms could have new functions, through mutations in the coding elements. We infer that *dsx* acquired the female-specific isoform that is expressed but not necessary for morphogenesis and later turned into an essential factor in female morphogenesis through some coding mutation such as the C-terminal extension. This process indicates that the neo-functionalization of the female-specific isoform of *dsx* was attained as a result of the accumulation of the coding mutations and fits well with the above prediction that coding mutations gives rise to functionalization of low adaptive isoforms.

### On the Origin of Outputs of the Sexual Differentiation Mechanism

**Fig. 7.**
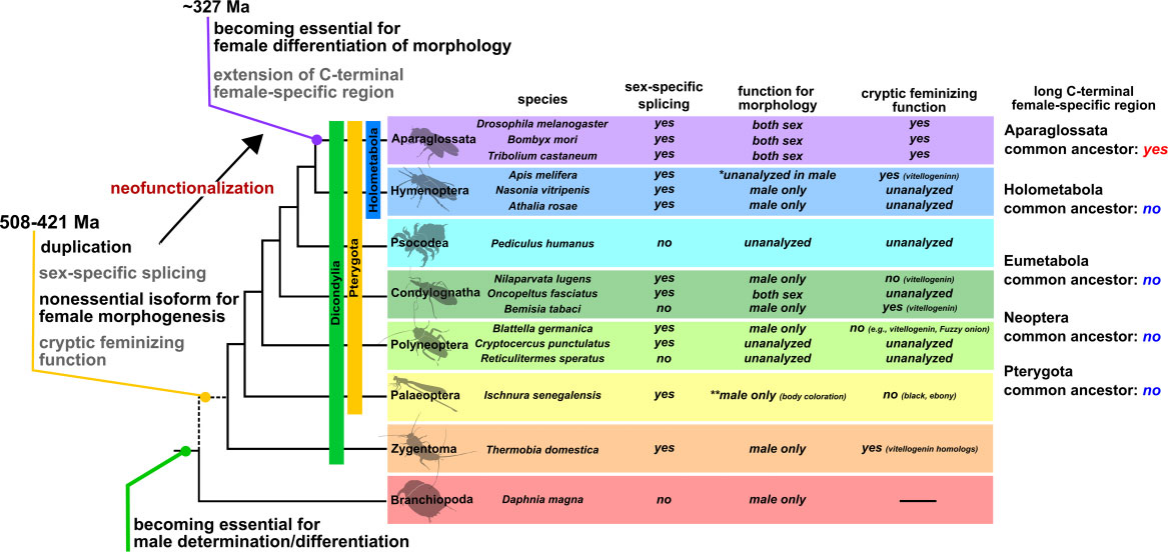
Schematic diagram of the evolutionary history of *doublesex* proposed in this study and the feature of *dsx* in insects. In our hypothesis, the female isoform of *dsx* may not have been essential for female differentiation of morphological traits at least from the common ancestor of Dicondylia to the common ancestor of Aparaglossata. In contrast, this female isoform may have contributed to the expression of some genes in females (“cryptic feminizing function” in the figure). The phylogenetic relationship and divergence time are referenced in Misof et al. (2014). The dotted line in the phylogenetic relationship indicates that the taxa occurring from the common ancestor between Branchiopoda and Dicondylia to the common ancestor of Dicondylia are omitted. Here, we also show information on the current knowledge of *dsx* features in insects and a branchiopod. In Aparaglossata, we show only three representative species. This information was based on the following studies: Hildreth (1965), Burtis and Baker (1989) and Clough et al. (2014) in *Drosophila melanogaster* (Diptera), Ohbayashi et al. (2001), Suzuki et al. (2003), and Xu et al. (2017) in *Bombyx mori* (Lepidoptera), Shukla and Palli (2012) in *Tribolium castaneum* (Coleoptera), Roth et al. (2019) and Velasque et al. (2018) in *Apis mellifera* (Hymenoptera), Wang et al. (2020) in *Nasonia vitripenis* (Hymenoptera), Mine et al. (2017, 2021) in *Athalia rosae* (Hymenoptera), Wexler et al. (2019) in *Pediculus humanus* (Psocodea) and *Blattella germanica* (Dictyoptera), Zhuo et al. (2018) in *Nilaparvata lugens* (Hemiptera), Just et al. (2021) in *Oncopeltus fasciatus* (Hemiptera), Guo et al. (2018) in *Bemisia tabaci* (Hemiptera), Miyazaki et al. (2021) in the wood roach *Cryptocercus punctulatus* and *Reticulitermes speratus* (Dictyoptera), Takahashi et al. (2019, 2021) in *Ischnura senegalensis* (Odonata), this study in *Thermobia domestica* (Zygentoma), and Kato et al. (2011) in *Daphnia magna* (Branchiopoda). In Condylognatha, information on *dsx* in the blood-sucking bug *Rhodnius prolixus* is omitted. *R. prolixus* has sex-specific isoforms of *dsx* whose function has not been investigated (Wexler et al. 2014). The term “unanalyzed” means the functional analyses of *dsx* have not been performed in the relevant species. Information on the roles of *dsx* of some species in female morphogenesis is limited to some body parts: body coloration in *I. senegalensis* (Takahashi et al. 2021), leg pigmentation, pheromone synthesis, and wing morphology in *Na. vitripenis* (Wang, Rensink, et al. 2022; Wang, Sun, et al. 2022), and worker morphology in *Ap. mellifera* (Roth et al. 2019). The asterisk (*) in *Ap. mellifera* indicates that the functional analysis of *dsx* in males was not conducted although gonad differentiation of female workers was affected by *dsx* knockouts (Roth et al. 2019; see main text). The double-asterisk (**) in *I. senegalensis* shows that this species has polymorphic coloration in females, that is, gynomorph (normal female color) and andromorph (male-like color) and that *dsx* is involved in color formation of males and andromorphic females but not gynomorphic females (see Takahashi et al. 2021), suggesting that *dsx* is nonessential for the female color development. In our hypothesis, the essential roles of *dsx* for female development in *O. fasciatus* (Just et al. 2021) may have occurred in parallel with Aparaglossata.

Recent studies on insects (Mine et al. 2017; Guo et al. 2018; Zhuo et al. 2018; Wexler et al. 2019; Takahashi et al. 2021), including this study, show that sexual differentiation mechanisms are diverse in their outputs as well as their gene repertoires. This output diversity is attributed to functional diversity of a single gene, *dsx*, in the sexual differentiation of morphogenesis during postembryonic development. Determining the evolutionary origin of diversity in the output is an enigmatic problem in sexual development. Data on the roles of *dsx* are limited to some traits in some species and are unavailable in many non-Aparaglossatan species, although functional analyses of *dsx* have been rapidly progressing using emerging model species. Unquestionably, comprehensive information on *dsx* functions for sexually dimorphic morphology from wider taxa is essential in order to fully tracing the evolution of *dsx*. We propose, albeit prematurely, the hypothesis that *dsx* may become essential for female differentiation in sexual morphology by expanding its cryptic feminizing role, that is, functions for expression of some female genes, in association with mutations in female-specific motifs (fig. 7). Since *dsx* of non-Aparaglossata has the female-specific exon but is not essential for female morphogenesis, the neo-functionalization is unlikely to depend on acquisition of the female-specific exon. This hypothesis can explain how single genes acquire novel outputs of sexual development although our hypothesis does not prevent alternative explanations from being proposed.

In this study, we have primarily discussed the functionality of *dsx* for sexual differentiation of morphology during postembryonic development. Therefore, it is unclear whether our conclusions about the evolutionary process apply to sexual behavior, including sex pheromone secretion and courtship, as well as sexual determination and gonadal differentiation during embryogenesis. Detailed studies of sex differences at various levels across insect taxa will test evolutionary hypotheses and help fully reconstruct the evolutionary history of *dsx* and sexual differentiation mechanisms.

## Materials and Methods

### Animals

The firebrat, *T. domestica* (Packard 1873), was used as an emerging model for apterygote. *T. domestica* is one of the species belonging to Zygentoma (Lepismatidae). The insects were kept at 37°C in total darkness condition and fed with fish food (TetraFin Goldfish Flakes, Tetra GmbH, Melle, Germany) in our laboratory. Stock colonies were reared in plastic cases of 30 cm×40 cm or 18 cm × 25 cm in length. Eggs were collected from tissue paper in the case and incubated at 37°C. For examining the roles of *dsx* and *dsx-like* in the postembryonic morphogenesis, colonies of hatched nymphs were reared up to the fourth instar in a six-well plate and then transferred into 24-well plates to be kept individually. For examining the roles of *dsx* and *dsx-like* in *vitellogenin* expression, female and male insects were collected from the stock colony and transferred into the plates. For examining the function of *dsx* and *dsx-like* for sexual morphology and gametogenesis, we used firebrats from April to June, 2019, February to April, April to July, and September to December, 2020. For investigating the roles of *dsx* and *dsx-like* in the *vitellogenin* expression, firebrats were manipulated from June to July, 2020.

### Estimation of Molt Timing

Estimating the molt timing of insects is essential for the analysis of developmental processes and the functions of developmental regulatory genes. The timing of Hemimetabolan or Holometabolan insects can be estimated using morphological changes such as a wing growth. However, timing is hard to estimate in apterygote insects since they have little change in their morphology during postembryonic development. *T. domestica* forms scales in the fourth instar, and changes the number and length of its styli during the fourth to ninth instar under our breeding conditions. These features can be used to estimate molt timing, but it is difficult to apply these criteria to experiments using adults or a large number of nymphs. To resolve this problem, we used leg regeneration after autotomy and time-lapse imaging to estimate the molt timing of *T. domestica*. Autotomy occurs at the joint between the trochanter and femur in *T. domestica*. An autotomized leg regenerates after one molt (Buck and Edwards 1990). For the RNAi analysis during postembryonic development, we amputated a right hindleg at the autotomic rift, using tweezers, and observed whether the leg had regenerated. This test enabled us to rapidly estimate the molt timing. For the RNA-seq and the RT-qPCR analysis, the time-lapse imaging was used to determine the precise time of molt. We build a time-lapse imaging system with a network camera system (SANYO, Tokyo, Japan) set in an incubator at 37°C (supplementary fig. S9*A*, Supplementary Material online). Photos of insects in the 24-well plate were taken every 5 min. We created a time-lapse movie from the photos every 12 h using ImageJ 1.52a (https://imagej.nih.gov/ij/) and observed whether the insects molted (supplementary fig. S9*B*, Supplementary Material online).

### De Novo Genome Assembly

A whole genome of *T. domestica* was sequenced to analyze the exon–intron structure of *dsx*. We selected an adult female of *T. domestica* from our stock colony and removed its alimentary canal. Genomic DNA was extracted from the sample using DNeasy Blood and Tissue Kit (QIAGEN K.K., Tokyo, Japan). A paired-end library was constructed from 1 µg of the DNA using TruSeq DNA PCR-Free LT Sample Prep kits (Illumina K.K., Tokyo, Japan) following the manufacturer’s instructions. The library was run on a sequencer (HiSeq 2500; Illumina K.K.). We obtained 417 Gb of raw reads and assembled them using Platanus v1.2.4 assembler (Kajitani et al. 2014) after removal of the adapter sequences. The genome sequence can be obtained from the DNA Data Bank in Japan (Accession number: DRA005797; Bioproject: PRJDB5781).

### Transcriptome Analysis

To search for *ds*x and *vitellogenin* (*vtg*) homologs, we performed RNA-seq analysis. Adults of 15 ♀♀ and 15 ♂♂ of *T. domestica* were sampled 1,440 min after a molt in December, 2019. The fat bodies of the individuals were removed using tweezers in a phosphated buffered saline (PBS; pH = 7.2). Three adults were used per sample. Total RNA was extracted from 10 samples (5♀♀, 5♂♂) using RNeasy Micro kits (QIAGEN K.K.) following the manufacturer’s instructions. The concentration of purified RNA was measured using a Qubit 4 fluorometer (QIAGEN K.K.) with Qubit RNA BR Assay kits (QIAGEN K.K.). Paired-end libraries were constructed from 100 ng of the total RNAs using TruSeq RNA Library Prep kits v2 (Illumina K.K.) following the manufacturer’s instructions. The libraries were run on a sequence (Hiseq, Illumina). The library preparation and sequencing were performed by Genewiz Strand-Specific RNA-seq service. We mapped the reads obtained to the assembled genome using the HISAT2 program (Kim et al. 2019) with a default option and counted the mapped reads using the STRINGTie program (Pertea et al. 2015) with default parameter settings. Differential expression gene analysis was performed based on the count matrix using the “edgeR” package (Robinson et al. 2010) in R-v4.0.3 (R Core Team 2020). Information about the samples can be obtained from the National Center for Biotechnology Information (NCBI) BioSample database (Accession numbers: SAMN18175012–SAMN18175021).

### Molecular Phylogenetic Analysis

Dsx is a member of the Doublesex and Mab-3 Related transcriptional factor (DMRT) family, and has a DNA-binding domain, Doublesex, and Mab-3 (DM) domain. Pancrustacea generally has four DMRT family genes, Dsx, Dmrt11, Dmrt93B, and Dmrt99B (Mawaribuchi et al. 2019). Phylogenetic analysis of DM domain-containing gene homologs was performed using the amino acid sequences of the DM domain. First, we used the Dsx sequences of *D. melanogaster* as a query and obtained 198 metazoan DM domain-containing proteins from the NCBI and the i5k databases (https://i5k.nal.usda.gov/) and our genome data of *T. domestica* by the BLAST analysis (listed in supplementary table S1, Supplementary Material online). We then aligned the sequences using MAFFT version 7 (Katoh and Standley 2013) with the -linsi option (to use an accuracy option, L-INS-i) and manually extracted the DM domain. The result of the multiple sequence alignment can be obtained from supplementary sequence file S1, Supplementary Material online. Molecular phylogenetic analysis of the aligned sequences was performed using a maximum-likelihood method with the IQ-TREE software (Minh et al. 2020). The substitution model was selected by -MPF and -AIC option of the IQ-TREE. The best-fit model was the LG + I+G4. The proportion of invariable sites is 0.0644, and the Gamma shape alpha was 0.7651. We used the ultrafast bootstrap (UFBoot) and Simodaira-Hasegawa approximate likelihood ratio test (SH-aLRT) to evaluate the branch reliability according to the manufacturer's recommendation (http://www.iqtree.org/doc/Frequently-Asked-Questions#how-do-i-interpret-ultrafast-bootstrap-ufboot-support-values). We set 1,000 replications in each test. Typically, the branch with the SH-aLRT ≥80% and the UFboot ≥95% would be reliable (Guindon et al. 2010; Minh et al. 2013). Therefore, we regarded the branch with both support values of more than the thresholds as the reliable clade.

Some previously reported dsx sequences were not grouped into the Insect Dsx Clade and were located close to other DMRT family genes, raising the possibility of the long-branch attraction due to rapidly evolving sequences. This problem might be a reason why some phylogenetic clades such as Pancrustacea Dsx are not robustly supported and why we failed to recover insect *dsx* clades. Thus, we excluded such problematic sequences and the relevant sequences, that is, other sequences from species with at least one fast-evolving sequence. The excluded sequences are indicated by colored in supplementary fig. S1 and table S1, Supplementary Material online. Then, we re-conducted the phylogenetic analysis using the remaining 166 sequences in the same way as the first reconstruction. The alignments were in supplementary sequence S2, Supplementary Material online. The best-fit model was the WAG + I+G4. The proportion of invariable sites is 0.0645, and the Gamma shape alpha was 0.6664. The clades interested in this study had strong supporting values (fig. 1). The full tree information (nexus format) can be obtained from the supplementary file, Supplementary Material online.

The position of noninsect hexapod’s Dsx such as Diplura and Collembola Dsx was still ambiguous within Hexapoda Dsx Clade since the relevant SH-aLRT/UFBoot values remained unreliable. The reason for the unreliability is not apparent to us. Typically, robust phylogenetic analyses are challenging in superfamilies of fast-evolved and deeply branched proteins (Chan and Ragan 2013) due to sequence divergence leading to unprecise alignments. Also, taxon sampling problems are a possibility. Since the sequence information on noninsect hexapods is rather limited, only a small number of sequences could be included in the analysis. When these sequences are enriched, the phylogenetic position of *dsx* in noninsect hexapods will be robustly estimated. Future tasks would focus on the relationship of noninsect hexapods.

### Full-length cDNA and Exon–intron Structures

To elucidate the exon–intron structures of Dsx and Dsx-like, we determined the full-length cDNA sequences using a Rapid Amplification of cDNA Ends (RACE) method and performed a BLAST analysis for our genome database of *T. domestica*. We extracted total RNA from eggs, whole bodies, fat body, and gonads of nymphs and adult females and males of *T. domestica* using TRI Reagent (Molecular Research Center Inc., Ohio, USA) following the manufacturer’s instructions. The total RNAs were treated with RNase-Free DNase I (New England BioLabs Japan Inc., Tokyo, Japan) to exclude remaining genomic DNA and purified by phenol/chloroform extraction and ethanol precipitation. For 5′-RACE analysis, mRNAs were purified from 75 µg of the total RNAs using Dynabeads mRNA Purification kit (Thermo Fisher Scientific K.K., Tokyo, Japan) following the manufacturer’s instruction. We then ligated an RNA oligo at the 5′-end of the mRNA using GeneRacer Advanced RACE kits (Thermo Fisher Scientific K.K.). For 3′-RACE analysis, we ligated an RNA oligo of the SMART RACE cDNA Amplification Kit (Takara Bio Inc., Shiga, Japan) at 3′-end of the total RNA during reverse transcription. First stranded (fs-)cDNA was generated from the RNAs using SuperScript III Reverse Transcriptase (Thermo Fisher Scientific K.K.). We used primers specific to the RNA oligos and performed RACE analysis by nested RT-PCR using Q5 High-Fidelity DNA polymerase (New England BioLabs Japan Inc., Tokyo, Japan). The primers specific to *dsx* and *dsx-like* were made from sequences of the relevant genomic regions and are listed in supplementary table S10, Supplementary Material online. The amplicons were separated using the agarose gel-electrophoresis and cloned using TOPO TA Cloning Kit for Sequencing (Thermo Fisher Scientific K.K.) following the manufacture’s protocol. We used a DH5α *Escherichia coli* strain (TOYOBO CO., LTD., Osaka, Japan) as the host cell. Plasmids were extracted using the alkaline lysis and purified by phenol–chloroform and ethanol precipitation. The nucleotide sequences of the cloned amplicons were determined from the purified plasmids by the Sanger Sequencing service of FASMAC Co. Ltd. (Kanagawa, Japan). We then searched the genomic region of the full-length cDNA sequences of *dsx* and *dsx-like* via local blastn analysis.

### Reverse Transcription-quantitative PCR

To quantitative mRNA expression levels, we performed RT-qPCR analysis. For investigating the sex-specific expression profile of *dsx* and *dsx-like*, we used the fat body of adults of *T. domestica* since the sexes can be distinguishable by the external morphology at this stage. Fat bodies also exhibit sex-specific physiological functions in adults. Thirteenth instar individuals and adults after molting were sampled for investigating roles of the genes in the sexually dimorphic morphology and the *vitellogenin* expression, respectively. The sample sizes are reported in the figure legends and supplementary table S3, Supplementary Material online. We dissected the individuals in PBS and collected their fat body in 2 ml tubes containing TRI Reagent (Molecular Research Center Inc., Ohio, USA). The fat bodies then were disrupted using a TissueLyser LT small beads mill (QIAGEN K.K.). These disrupted samples were preserved at −80°C until used. Total RNA was extracted from the samples according to the manufacture’s protocol for the TRI Reagent. Extracted RNA was treated with 2% RNase-free DNase I (New England BioLabs Japan Inc.) at 37°C for 40 min and purified by phenol/chloroform extraction and ethanol precipitation. We measured the concentration of the total RNA using a spectrophotometer (DS-11+, Denovix Inc., Wilmington, USA). fs-cDNA was synthesized from 350 ng of the total RNA using SuperScript III Reverse Transcriptase (Thermo Fisher Scientific K.K.). We diluted the fs-cDNA to 1:2 with MilliQ water and preserved it at −30°C until it was used in RT-qPCR assay. The RT-qPCR assays were performed using a LightCycler 96 instrument (Roche, Basel, Switzerland) according to the manufacture’s protocol with the THUNDERBIRD SYBR qPCR Mix (TOYOBO Co. Ltd.). The reaction volume was 10 µl. We used 1 µl of the fs-cDNA as templates. The preparation of the RT-qPCR solution proceeded on ice. The protocol of the RT-qPCR was as follows: preincubation at 95°C for 600 s and 45 cycles of three-step reactions, such as denaturation at 95°C for 15 s, annealing at 60°C for 15 s and extension at 72°C for 45 s. We used *ribosomal protein 49* (*rp49*) as a reference gene, as described by Ohde et al. (2011). We designed primer sets of the target genes by the Primer3Web version 4.1.0 (Untergasser et al. 2012) following the manufacture’s recommended condition of the THUNDERBIRD SYBR qPCR Mix. We confirmed the primers’ specificity using melting curves ranging from 65°C to 95°C. We selected primer sets exhibiting a single peak. The primers are listed in supplementary table S10, Supplementary Material online. Each RT-qPCR was technically replicated three times. Some samples were excluded before analyzing the data when the Ct value of any genes was not detected in one or more replicates or when the Ct value of the reference gene deviated from that of other samples. In these removed data, a technical error was suspected. We calculated the expression level of target genes by the 2^–ΔΔCt^ method (Livak and Schmittgen 2001) and performed the Brunner–Munzel (BM) test for ΔCt value. The BM test was carried out using R-v4.0.3. with the *brunnermuzel.test* function of the “brunnermuzel” package (https://cran.r-project.org/web/packages/brunnermunzel/index.html). Holm’s method was used for multiple comparison analyses between the control and treatments. The data are listed in supplementary table S3, Supplementary Material online. In the *dsx* expression of the RNAi male, we performed the Smirnov-Grubbs (SG) test for ΔCt value using the *grubbs.test* function of the “outliers” package in R (https://cran.r-project.org/web/packages/outliers/index.html) (supplementary table S4, Supplementary Material online). An outlier was detected in the *dsx* RNAi male. We repeatedly performed the SG test using the data excluding the outlier. No further outliers were detected. Finally, we re-analyzed the data, excluding the outlier, using the BM test (supplementary table v3, Supplementary Material online).

### RNAi Analysis

The RNAi assay can be used to examine the roles of genes during postembryonic development in *T. domestica* (Ohde et al. 2011). The sexual differentiation of insects is generally assumed to be a cell-autonomous mechanism that is independent of systemic hormonal control (Verhulst and van de Zande 2015) as discussed in De Loof and Huybrechts (1998) and Bear and Monteiro (2013) and progresses during postembryonic development. Therefore, nymphal RNAi is the most effective tool to investigate the roles of genes on sexual trait formation during postembryonic development. To reduce the risk of off-target effects, the dsRNA was designed to avoid the region of the DM domain. We also confirmed that the dsRNA had no contiguous matches of more than 20 bases with other genes on the genome by BLAST (blastn option). To produce templates for the dsRNA, we cloned the regions of *dsx* and *dsx-like* from the fs-cDNA using the same method as the RACE analysis. We amplified the template DNAs from purified plasmids with PCR using Q5 High-Fidelity DNA Polymerase and purified the amplified DNA with the phenol/chloroform extraction and the ethanol precipitation. dsRNA was synthesized from the purified DNA using Ampliscribe T7-Flash Transcription kits (Epicentre Technologies, Co., Wisconsin, USA). We designed the PCR primers using the Primer3Web version 4.1.0 (Untergasser et al. 2012). The PCR primers are listed in supplementary table S10, Supplementary Material online. In nymphal RNAi analysis, we injected the dsRNAs repeatedly into the abdomen of the nymphs of *T. domestica* with each molt from the fourth or fifth instar to thirteenth instar to sustain the RNAi effect during postembryonic development. The initial stage was the same within a single experiment. This repeated RNAi treatment was effective in some insects such as *Bl. germanica* (Wexler et al. 2019). We sampled the individuals 1, 3, and 5 days after molting, using phenotypic observations, analysis of *dsx* knockdown effects, and the oocyte number. To determine the sex of individuals, we initially observed the gonads: testis and ovary. In our RNAi analysis, the gonads completely formed and there was no difference between the control and *dsx* RNAi individuals in external morphology (fig. 3*C*). Therefore, individuals with testis were males and those with ovaries were females. *T. domestica* molts throughout its life, even after sexual maturation, and produces *vtg* during each adult instar (Rousset and Bitsch 1993). To analyze the *vtg* mRNA levels, we also injected the dsRNAs of *dsx* and *dsx-like* repeatedly into the females and males every 3 days from 12 h after molting. We sampled the females and males at 720 ± 20 min after subsequently molts.

### Phenotype Observation

We dissected thirteenth instar individuals in PBS using tweezers and removed the thoraxes, reproductive systems, and external genital organs. We took images using the digital microscope system (VHX-5000, KEYENCE, Tokyo, Japan). The thoraxes and external genital organs were fixed with FAA fixative (formaldehyde: ethanol: acetic acid = 15:5:1) at 25°C overnight and then preserved in 90% ethanol. We used the length of the prothorax as an indicator of body size. To measure the prothoracic width, the prothoracic notum was removed from the fixed thorax after treatment with 10% NaOH solution at 60°C for 30 min to dissolve the soft tissues. The notum was mounted in Lemosol on a microscope slide. The prepared specimens were imaged using a KEYENCE VHX-5000. With the microscope at 50×, the length of the notum was measured. The ovipositor length was also measured using the microscope at 20× and 50×. To count the sperm number, sperm was collected from seminal vesicles and diluted with 5 ml MilliQ water. 50 µl of the diluted sperm was spotted on a microscope slide and dried overnight. We technically replicated the measurement three times for ovipositor length and six times in sperm number and calculated these means. Measurement was performed by blinding the treatment. We counted the number of oocytes in ovarioles using an optical microscope at 50× (Olympus, Tokyo, Japan). A GLM was used to analyze differences in ovipositor length (length data) and sperm and oocyte number (count data) among RNAi treatments. The body size, target genes, and interactions between the target genes were used as explanatory variables. The length was assumed to follow a Gaussian distribution, and the count data to have a negative binomial distribution. We used R-v4.0.3 in these analyses and the *glm* and the *glm.nb* (MASS package) functions for the length and count data, respectively. To analyze the contribution of the explanatory variables, a likelihood ratio test for the result of GLM was performed using the *Anova* function of the car package. The statistical results are listed in supplementary tables S5 (female) and S6 (male), Supplementary material online.

### Scanning Electron Microscopy

The NanoSuit method (Takaku et al. 2013) was used for the scanning electron microscopy (SEM) analysis. Male penises and female ovipositors preserved in 90% ethanol were washed with distilled water and immersed in 1% Tween20 at 25°C for 10 min. The samples were mounted on stubs and imaged using a low-vacuum SEM (DX-500; KEYENCE).

### Histology

The gonads of RNAi individuals were fixed with Bouin’s fixative (saturated picric acid: formaldehyde: glacial acetic acid = 15:5:1) at 25°C overnight and washed with 90% ethanol plus Lithium Carbonate (Li_2_CO_3_). The ovipositors of RNAi individuals were fixed with FAA fixative at 25°C overnight and then were transferred into 90% ethanol. The samples were dehydrated and cleared with an ethanol-butanol series. The cleared samples were immersed and embedded in paraffin at 60°C. The paraffin blocks were polymerized at 4°C and cut into 5-µm thick sections using a microtome (RM2155: Leica, Wetzlar, Germany). The sections were mounted on microscope slides coated with egg white-glycerin and stained using Delafield’s Hematoxylin and Eosin staining. After staining with the hematoxylin, the slides were washed with 1% hydrochloric acid–ethanol for 40 s. The stained slides were enclosed with Canada balsam. We observed the slides on an optical microscope (Olympus) and took photos using a digital single-lens reflex camera (Nikon, Tokyo, Japan).

### Ancestral Sequence Reconstruction

To infer the sequence evolution of the Dsx, we conducted an ancestral sequence reconstruction (ASR) of the C-terminal sequences of the Dsx female-type homologous sequence. First, we searched homologous sequences to Dsx female-type from NCBI protein/transcript shotgun assembly databases and previous studies. The searches in the NCBI databases were performed by the BLAST search. We closely examined the alignment results of the BLAST and selected sequences with at least 10 amino acids aligned with the female-specific region of each query sequence. We do not know whether some of these sequences are expressed in females and contribute to female morphogenesis, as these sequences have not been investigated their expression profiles and function in the species. We decided that it was not problem to use these sequences since we focused on the evolution of sequences homologous to Dsx female-type in each insect taxa. In Diptera, we set Dsx female-type of *D. melanogaster* (Accession #: NP_001287220) as a query and obtained nine sequences. In Lepidoptera, we used Dsx female-type of *Bombyx mori* (NP_001036871) as a query and get 10 sequences. In Coleoptera, *dsx* female-type of *Tribolium castaneum* (AFQ62106) was set in a query and then 10 sequences were obtained. We used Dsx female-type of *Ap. mellifera* (NP_001128407) and *At. rosae* (XP_012262256) as queries to search hymenopteran sequences. We also searched some hymenopteran sequences from the NCBI databases based on a previous study (Baral et al. 2019). 10 hymenopteran sequences were obtained. To obtain sequences of Psocodea and Hymenoptera, we set the sequences of *Pe. humanus* (QGB21102) and *Rhodonius prolixus* (QGB21099) as queries and searched the NCBI blast database. Wexler et al. (2019) showed that Dsx of *Pe. humanus* (Psocodea) has isoforms without sex-specificity. In this study, based on the blast search and exon structure, we regarded that the PhDsx1 in Wexler et al. (2019) is homologous to the Dsx female-type. The sequences of *Ni. lugens* (AWJ25056) and *Bl. germanica* (QGB21105 and QGB21106) were obtained from the database based on previous studies (Zhuo et al. 2018; Wexler et al. 2019). We selected two sequences from *Bl. germanica*, as this species has two female-specific Dsx isoforms (Wexler et al. 2019). The sequences of *Cryptocercus punctulatus* and *I. senegalensis* were obtained from previous studies (Miyazaki et al. 2021; Takahashi et al. 2021). In *T. domestica*, the sequence identified in this study was used. The sequence names are listed in supplementary table S8, Supplementary Material online. We then manually extracted the OD domain and performed multiple sequence alignments (MSA) using the MAFFT version 7 (Katoh and Standley 2013) with the -linsi option (supplementary sequence file 3, Supplementary Material online). We reconstructed ASs from the MSA using MEGA X software. The maximum-likelihood method was applied to the ASR. The JTT + G model was chosen as a substitution model by AIC-based model selection. The guide tree was reconstructed based on previously reported phylogenetic relationships (Wiegmann et al. 2011; Misof et al. 2014; Li et al. 2017; Peters et al. 2017; Zhang et al. 2018; Kawahara et al. 2019; McKenna et al. 2019; Gustafson et al. 2020) (supplementary fig. S10, Supplementary Material online). We selected the most probable sequences for the following analyses. The results of ASR can be seen in supplementary table S9, Supplementary Material online. The probabilities of sites of AS that we focused on are listed in supplementary table S11, Supplementary Material online. In Aparaglossata (Node 77) and Holometabola (Node 87) AS, almost all probabilities of sites were more than 0.9. Except sites were sites 83 and 98 in Node 77 and sites 77–79 and 83 in Node 87. These sites other than sites 77 had probabilities >0.5. Thus, we concluded that the AS in Aparaglossata and Holometabola, which we considered the most critical, was reconstructed with sufficient reliability. No residues had the probabilities = 0 in the Aparaglossata-specific region of Holometabola AS. In contrast, in nonholometabolan insects, since our taxon sampling is limited to several species (Eumetabola in Node 92, Neoptera in Node95, Pterygota in Node 96), the probabilities of some sites are lower than 0.5. These low probable sites are not necessarily confident. To conclude with reliability, it is no doubt that analyses based on a larger number of species will be essential. However, all sites of the Aparaglossata-specific region in these AS were gaps with the probabilities >0.9. The result of the sites of the Aparaglossata-specific region seems to be relatively reliable in our analysis. Thus, our conclusion that the Aparaglossata-specific region occurred in the common ancestor of Aparaglossata would be confident. To compare the sequences, we then performed MSA of the most probable reconstructed ASs and the sequence of *D. melanogaster* using MAFFT version 7 (fig. 6*A*).

### Protein Structure Prediction

To infer the evolution of protein structures of Dsx, we conducted the protein structure prediction. The ASs reconstructed by the above section were used for the protein structure prediction. The sequences were obtained from supplementary sequence file 3, Supplementary Material online. The protein structure prediction was performed using the Alphafold2-based algorism (ColabFold: Mirdita et al. 2021) with the default option. The accuracy of predictions was evaluated based on the predicted Local distance difference test (plDDT) score that was automatically calculated on the ColabFold. We selected a model with the highest average plDDT score in each prediction. The average plDDT scores were 81.824 (Aparaglossata), 89.165 (Holometabola), 87.376 (Eumetabola), 90.721 (Neoptera), and 90.720 (Pterygota). The plDDT scores were >70 in the helical structure predicted as the α-helix loop of the female-specific Dsx region. Generally, predicted structures of plDDT > 70 are regarded to be a confident prediction (cf., Tunyasuvunakool et al. 2021). Therefore, we assessed the α-helix loop of the female-specific region of Dsx as the confidently predicted structure. The graph of the plDDT score of each model is shown in supplementary fig. S11, Supplementary Material online. The 3D models of predicted structures were visualized with the PyMOL Molecular Graphics System, Version 2.0 (Schrödinger, LLC.). On the viewer, we colored the female-specific region and the Aparaglossata-specific region with red color and the green color, respectively.

## Supplementary Material

msac145_Supplementary_Data

## Data Availability

The draft genome data were deposited in the DNA Data Bank of Japan (Accession number: DRA005797; Bioproject: PRJDB5781). The raw read data of the transcriptome were in the NCBI Sequence Read Archive (Accession numbers: SRR13870115–SRR13870124; Bioproject: PRJNA707122). The sequences of *dsx* male-type, *dsx* female-type, and *dsx-like* are also in GenBank (Accession numbers: MW711323, MW711324, and MW711325, respectively). We deposited the data sets used for generating supplementary tables S3, S5, and S6, fig. 1A, supplementary fig. S1 in the figshare (doi:10.6084/m9.figshare.20103809).
