# Supplementary material for: Evolutionary History of Sexual Differentiation Mechanism in Insects
Source: Mol Biol Evol. 2022 Jul 12;39(7):msac145. doi: 10.1093/molbev/msac145 (PMC9290531; doi:10.1093/molbev/msac145)
Supplement: msac145_Supplementary_Data [file msac145_supplementary_data.zip › Supplementary_materials.docx]

**Supplementary Material for**

**Evolutionary history of sexual differentiation mechanism in insects**

Yasuhiko Chikami, Miki Okuno, Atsushi Toyoda, Takehiko Itoh, Teruyuki Niimi

Teruyuki Niimi

Email: [niimi@nibb.ac.jp](mailto:niimi@nibb.ac.jp)

**This PDF file includes:**

Supplementary text

Supplementary Figures 1 to 11

Caption of Supplementary Tables 1 to 11

Supplementary Sequence 1 to 3

Supplementary references

**Other supplementary materials for this manuscript include the following:**

Supplementary Tables 1 to 11: an EXCEL file

Supplementary File tree information: a nexus format file (.txt)

**Supplementary Text**

**Detailed results**

The general morphology of the external genital organs and the reproductive systems in Zygentoma is reviewed in Matsuda (1976). The recent interpretation of the penial structure and its evolution are explained in Boudinot (2018). The ovipositor structure and its evolution are detailed in Emeljanov (2014). Here, we described the morphology of the genital organs and the reproductive systems in *Thermobia domestica* with our focus points.

**Reproductive system and germline cell morphology in males**

In males of *T. domestica*, a pair of testes was located on the dorsal side of the abdomen. The testis was consisted of some testicular follicles (fig. 3C, D). Each testicular follicle was connected to the vas deferens via the vas efferens (fig. 3D). The seminal vesicle lay between the vas deferens and the ejaculatory duct. A pair of the ejaculatory ducts was associated with each other in the front of the gonopore in the penis (fig. 3D). The testicular follicles were a bean-like shape and the seminal vesicles were a bean pod-like shape. In the testicular follicle, the spermatogonia was in the antero-most part (fig. 3C). The primary and secondary spermatocytes lay in the middle part. In the posterior part of the testicular follicle, there were some sperm bundles (fig. 3C). The wall of the testicular follicle consisted of a single flattened epithelial layer.

We observed the above features of the reproductive system in *dsx* or *dsx-like* RNAi males (fig. 3C, D). In *dsx* and both genes RNAi males, the seminal vesicles were rounded shape. The vas efferens was filled with the sperm (fig. 3D). In contrast, we could not find differences in the morphology of the testicular follicles or spermatogenesis between the RNAi and control males. The male reproductive system and spermatogenesis showed no visible difference between *dsx* female-type and *dsx-like* RNAi females and the control ones.

**Reproductive system and germline cell morphology in females**

In females of *T. domestica*, part of the ovary was on the dorsal side of the abdomen. Each ovary consists of five ovarioles and was attached to the anterior part of the abdomen via the terminal tuft (fig. 3F). The ovarioles were associated with each other at the lateral oviduct. The lateral oviduct was connected to the common oviduct and subsequently opened at the gonopore in the valvula I. There was no vagina between the gonopore and the oviduct. The spermatheca was located on the branch point of the common oviduct along the midline (fig. 3F). The spermatheca was divided into two parts: anterior and posterior (supplementary fig. 4). The anterior part consisted of a pseudostratified layer of the columnar epithelial cells that were secretory. The posterior part was surrounded by a single layer of epithelial cells. The ovariole was panoistic-type and was composed of two parts: the germarium and the vitellarium fig. 4C). The germarium contained many oogonia and young oocytes. The vitellarium had previtellogenic and vitellogenic oocytes. The oocytes in the vitellarium were surrounded by a single layer of follicle cells. There were pedicel cells in the terminal of the ovariole. The previtellogenic oocyte had a large germinal vesicle and basophilic cytoplasm. The vitellogenic oocyte was elongated along the anterior-posterior axis of the ovariole and had eosinophilic cytoplasm. Many eosinophilic lipid droplets were present in the peripheral region of the vitellogenic oocytes. The follicle cells were flattened and columnar in shape in the previtellogenesis and the vitellogenesis.

We observed the above features of the reproductive system in *dsx* or *dsx-like* RNAi females (fig. 3C, F; supplementary fig. 4). We could not detect visible differences in the female reproductive system or oogenesis between the RNAi females and the controls. This result suggests that the *dsx* and *dsx-like* have no function in the formation of female traits and gametogenesis at the tissue and cellular level.

**General morphology of the external genital organ in *Thermobia domestica***

The description below is based on the observation of the controls, i.e., individuals injected *egfp* dsRNA, and is agreed with previous studies in *T. domestica* (Snodgrass 1957; Matsuda 1976; Emeljanov 2014; Boudinot 2018).

*T. domestica* males have a single penis. This penis is an unpaired appendix on the abdomen segment IX and is not copulatory organ (Matsuda 1976). The penis was sub-segmented into two parts. There were many setae on the left and the right side of the distal tips (fig. 4C). The surface of the penis had a reticulated pattern (fig. 4C). This simple penial structure was presumably gained at the last common ancestor of Ectognatha (= Archaeognatha + Zygentoma + Pterygota: Insecta s.str.) (*Boudinot, 2018*).

*T. domestica* females has an ovipositor. This ovipositor consists of two pairs of appendices (gonapophysis) and is derived from the retracted vesicles on the abdomen VIII and IX (Matsuda 1976; Emeljanov 2014). This ovipositor is an autapomorphy of Ectognatha (Kristensen 1975; Beutel 2017). The gonapophyses on the abdomen VIII (valvula I) were the ventral part of the ovipositor and a paired structure. The gonapophyses on the abdomen IX (valvula II) were the dorsal side of the ovipositor and were united to form an unpaired structure (supplementary fig. 5B). The distal tip of the valvula II remained a paired structure and possessed dense setae (fig. 4E), which may play a role in sensory reception. Both valvulae were sub-segmented and have some setae (fig. 4E). The valvula I and II were connected through a tongue-and-groove structure (olistheter). The olistheter consisted of an aulax (”groove”) on the valvula I and a rhachis (”tongue”) on the valvula II (supplementary fig. 5). Within the valvulae, the epithelial cells were beneath the cuticular layer. The cuticular layer was thickened and multi-layered in the outer surface of the ovipositor. In contrast, the inner surface (i.e., the side of the egg cavity) of the ovipositor had a thin and single-layered cuticle. Some lumens of the valvulae were extended along the anterior-posterior axis and were hemocoelic cavities.

Effects of knockdown of *dsx* and *dsx-like* on the external genital organ

In *dsx* RNAi males, a tubular organ was formed instead of the penis (fig. 4B, C). This tubular organ consisted of two pairs of appendage-like structures. The inner one is connected to the gonopore and the ejaculatory duct. The outer one had a lot of setae on its tip (fig. 4C). Thus, the inner pair was similar to the valvula I of the female ovipositor and the outer one was similar to the valvula II. We could detect sub-segmentation in both structures (fig. 4C). These features indicated that the tubular organ in the *dsx* RNAi males was parallel to the female ovipositor. The same phenotype was found in the *dsx* and *dsx-like* double RNAi males. In contrast, the *dsx-like* males possessed a penis the same as that of the control insects.

In females that were treated with RNAi for *dsx*, *dsx-like*, and both genes, the external genital organ was the same as the ovipositor of the control females that described in the above section (fig. 4E, F). This genital organ of RNAi females consisted of two pairs of sub-segmented appendage-like structures and possessed dense setae on the tip of the inner pair. The outer pair was connected to the gonopore and the common oviduct. Thus, in the view of histology, the location, and the relation to other elements, the external organ of the RNAi females was not different from the ovipositor of the control ones.

**Supplementary FIG. 1.**
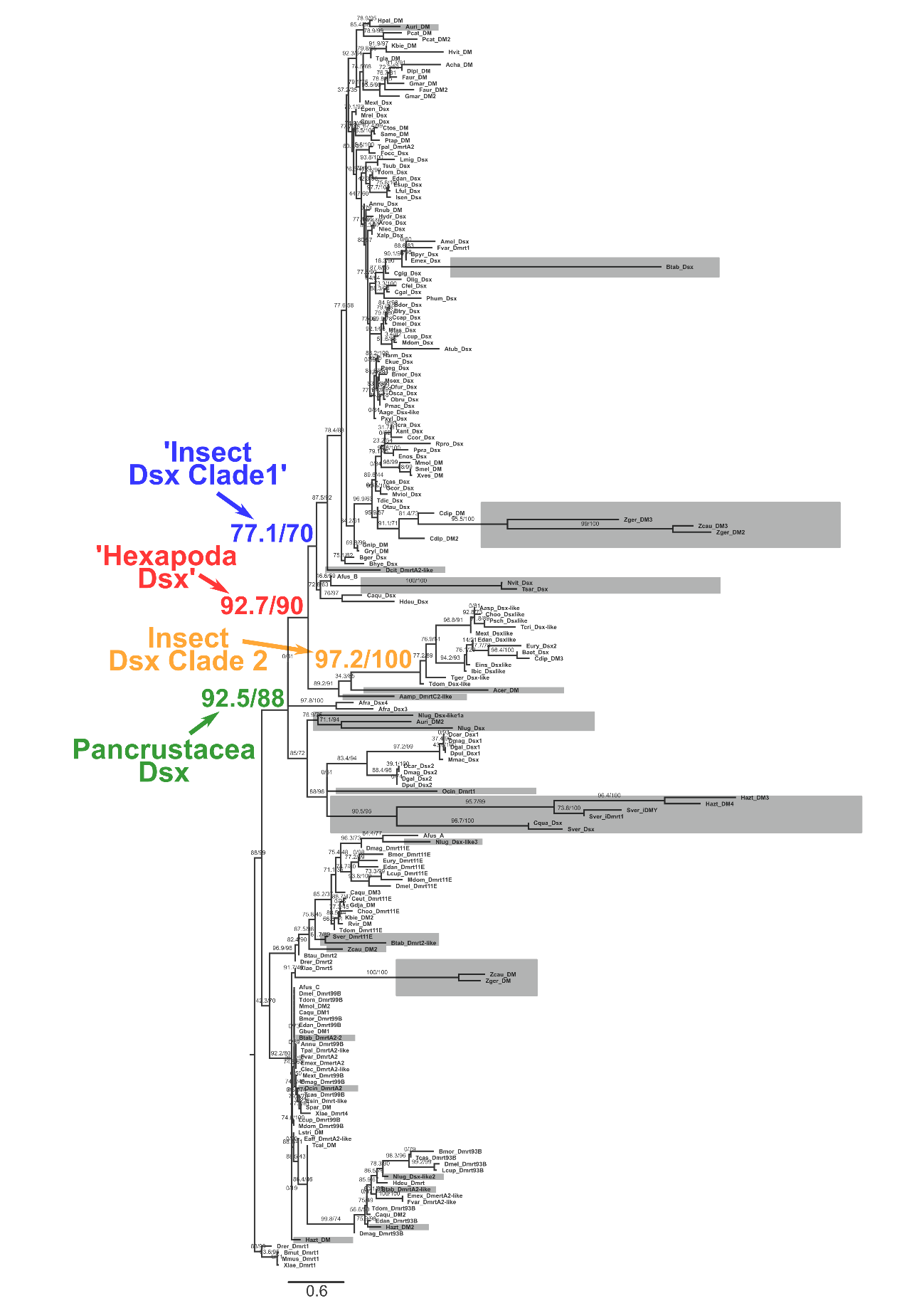
 Initial phylogenetic tree of Dmrt family. The tree was reconstructed using 198 DM domain-containing sequences. The numbers on branches indicates SH-aLRT/UFBoot. Grayed OTUs shows excluded sequences in the re-analysis.

**Supplementary FIG. 2.**
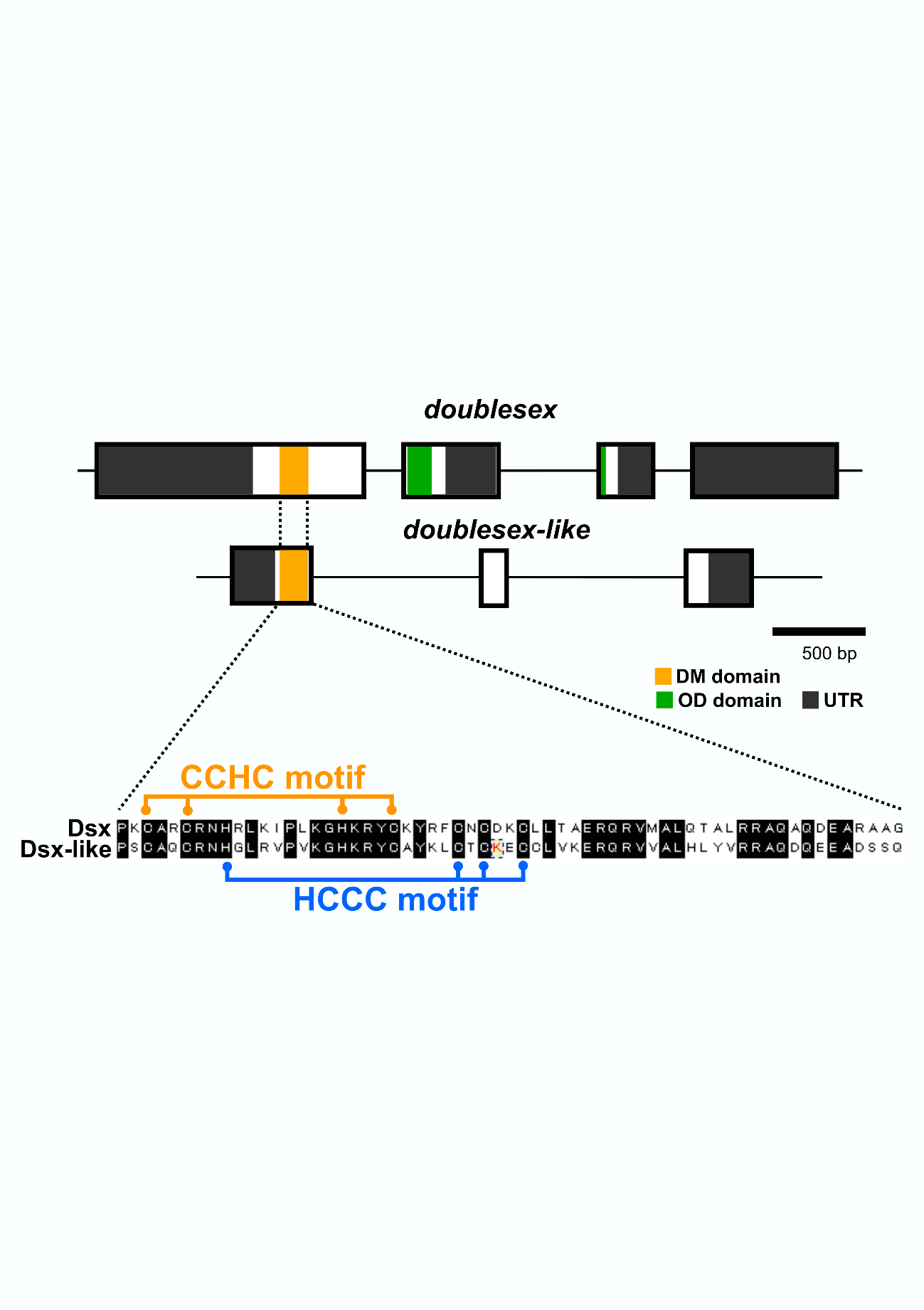
 Structure of DM domain of Dsx and Dsx-like of *T. domestica*. The upper schemes show the exon-intron structure of *dsx* and *dsx-like*. The lower alignment indicates the amino acid sequences of the DM domain in Dsx and Dsx-like. The black-background sites are identical between the two proteins. The intertwined Zinc Finger structure is retained between the proteins. Orange and blue colors indicate the CCHC and the HCCC motifs.

**
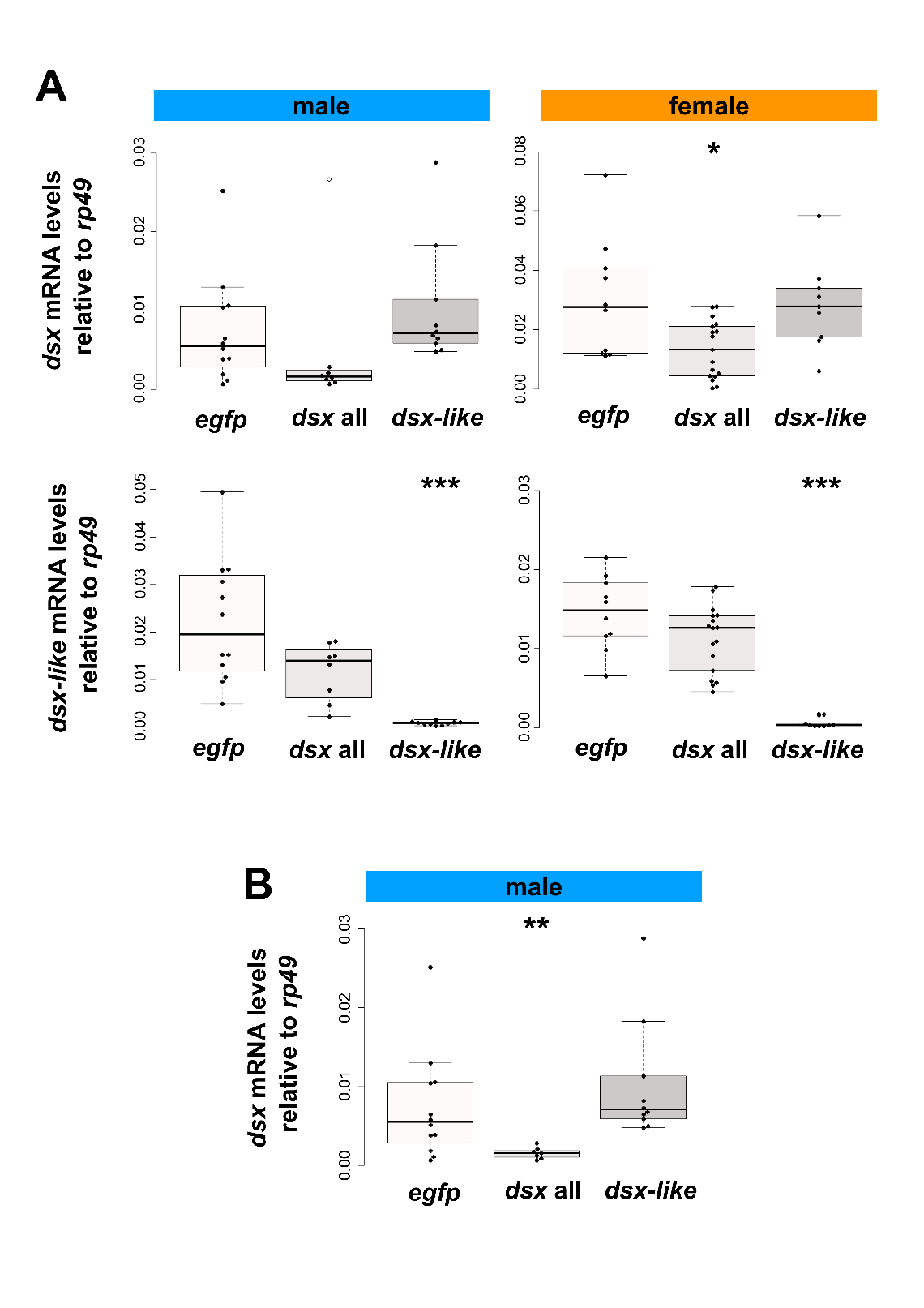
Supplementary FIG. 3.** Expression of *dsx* and *dsx-like* mRNA in nymphal RNAi individuals. (*A*) Expression level of target genes in RNAi individuals. The mRNA levels of *dsx* and *dsx-like* were analyzed by RT-qPCR assay and are the relative value to the expression of the reference gene, *ribosomal protein 49* (*rp49*). The upper graphs are the expression of *dsx* and the lower ones are that of *dsx-like*. The left column is the result in males and the right one is that in females. White plot suggests the outlier. (*B***)** the expression level of *dsx* mRNA in the nymphal RNAi males after excluding an outlier. To test the outlier, the Smirnov–Grubbs’ test was performed. The result of the Smirnov–Grubbs’ test is shown in supplementary table 4. The *egfp*, *dsx* all and *dsx-lik*e indicates the *egfp* dsRNA injected group (control), *dsx* sex-common region dsRNA injected group and *dsx-like* dsRNA injected group, respectively. Results of the Brunner–Munzel test are indicated by asterisks: **P* < 0.05; ***P* < 0.01; ****P* < 0.001 and is also described in supplementary table 3. *P* ≥ 0.05 is not shown. Each plot indicates the value of each individual. Total *N* = 30 and 36 in males and females.

**
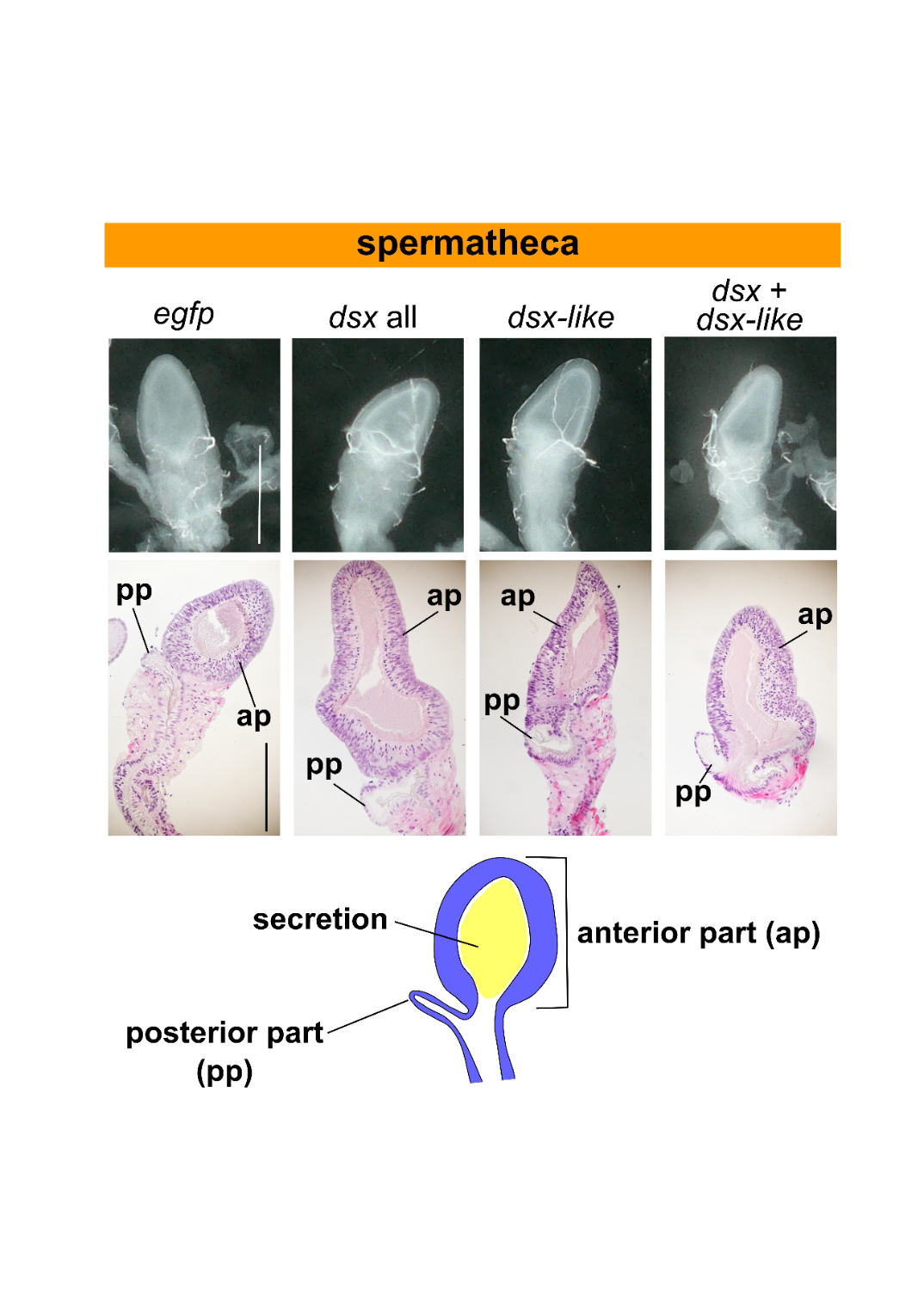
Supplementary FIG. 4.** Morphology of spermatheca in nymphal RNAi females. The upper photos show the light microscopic images of the spermatheca. The middle ones are paraffin sections of the spermatheca. Hematoxylin-Eosin staining. The lower one is the schematic image of the spermatheca of *T. domestica*. The *egfp*, *dsx* all, *dsx-lik*e and *dsx*+*dsx-like* indicates the *egfp* dsRNA injected group (control), *dsx* sex-common region dsRNA injected group, *dsx-like* dsRNA injected group, and both *dsx* sex-common region and *dsx-like* dsRNAs injected group, respectively. The spermatheca is divided into two parts: anterior and posterior part and have secretion within its lumen. Scales: 500 µm. ap, anterior part of spermatheca; pp, posterior part of spermatheca. The detailed description can be seen in Supplementary Material.

**
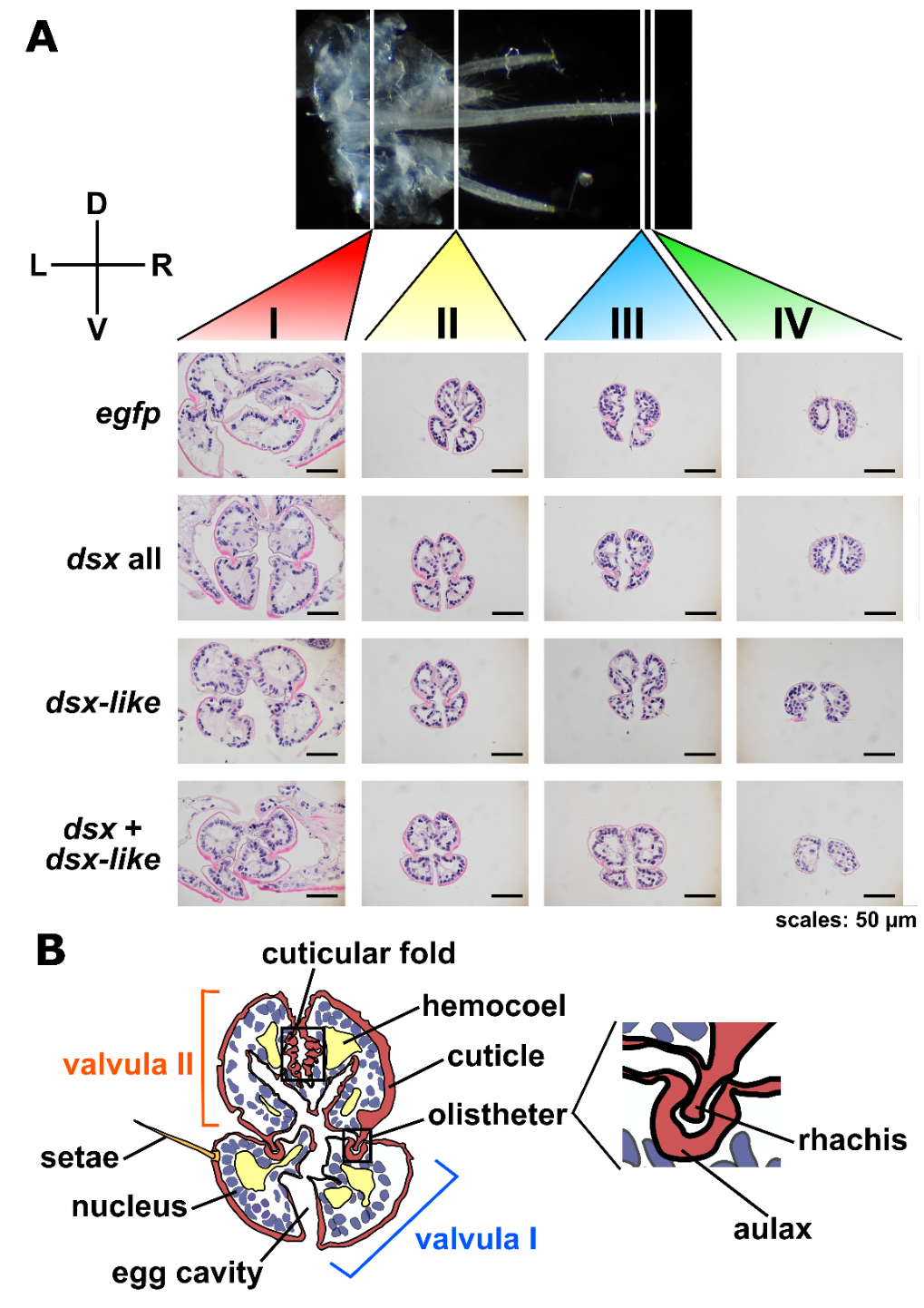
Supplementary FIG. 5.** Morphology of ovipositor in nymphal RNAi individuals. (A) Cross-section of the ovipositor. The photos show the morphology of the ovipositor in four parts: I (proximal part), II (middle part), III (distal part), and IV (most-distal part). The *egfp*, *dsx* all, *dsx-lik*e and *dsx*+*dsx-like* indicates the *egfp* dsRNA injected group (control), *dsx* sex-common region dsRNA injected group, *dsx-like* dsRNA injected group, and both *dsx* sex-common region and *dsx-like* dsRNAs injected group, respectively. D, dorsal; L, left; R, right; V, ventral. Paraffin. Hematoxylin-Eosin staining. Scales: 50 µm. (B) Schematic figure of the ovipositor morphology. This figure is based on the cross-section of the part II in the control female. The part of ovipositor is constituted of two regions: valvula I and II. These regions are coordinated at the olistheter. The dorsal side of valvula II has folded cuticle. The detailed description can be seen in Supplementary Material.

**
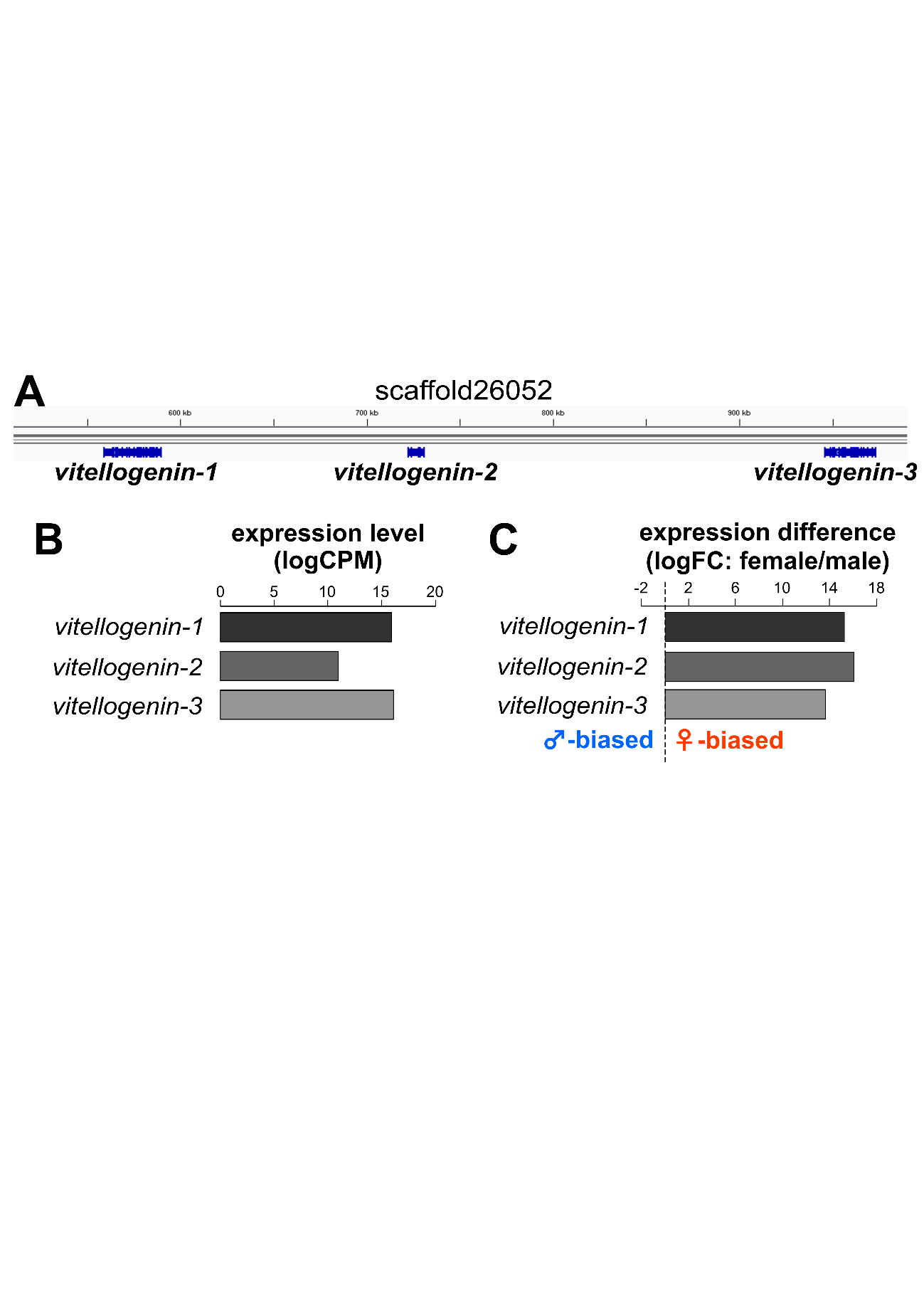
supplementary FIG. 6.** Expression of *vitellogenin* homologs in *T. domestica*. (*A*) Genome mapping of *vitellogenin* mRNA sequences. The picture is a screenshot of the integrative genome viewer (IGV). Vitellogenin genes are located on scaffold26052 of the assembled genome. *T. domestica* has three tandem-repeated *vitellogenin* homologs: *vitellogenin-1*, *vitellogenin-2*, and *vitellogenin-3*. (*B*) Expression level of *vitellogenin* homologs. The expression levels were calculated by the logCPM of transcriptome data in the fat body of males and females. All *vitellogenin* homologs show the high expression level in the fat body. (*C*) Difference in expression of *vitellogenin* homologs between sexes. The differential expression analysis was performed using the edgeR program. The expression difference is shown by the logFC value. When the values are more than 0, genes are expressed higher in females than males. Thus, all *vitellogenin* homologs shown here are expressed much higher in females than in males. Each value in (*B*) and (*C*) can be seen in supplementary table 7.

**
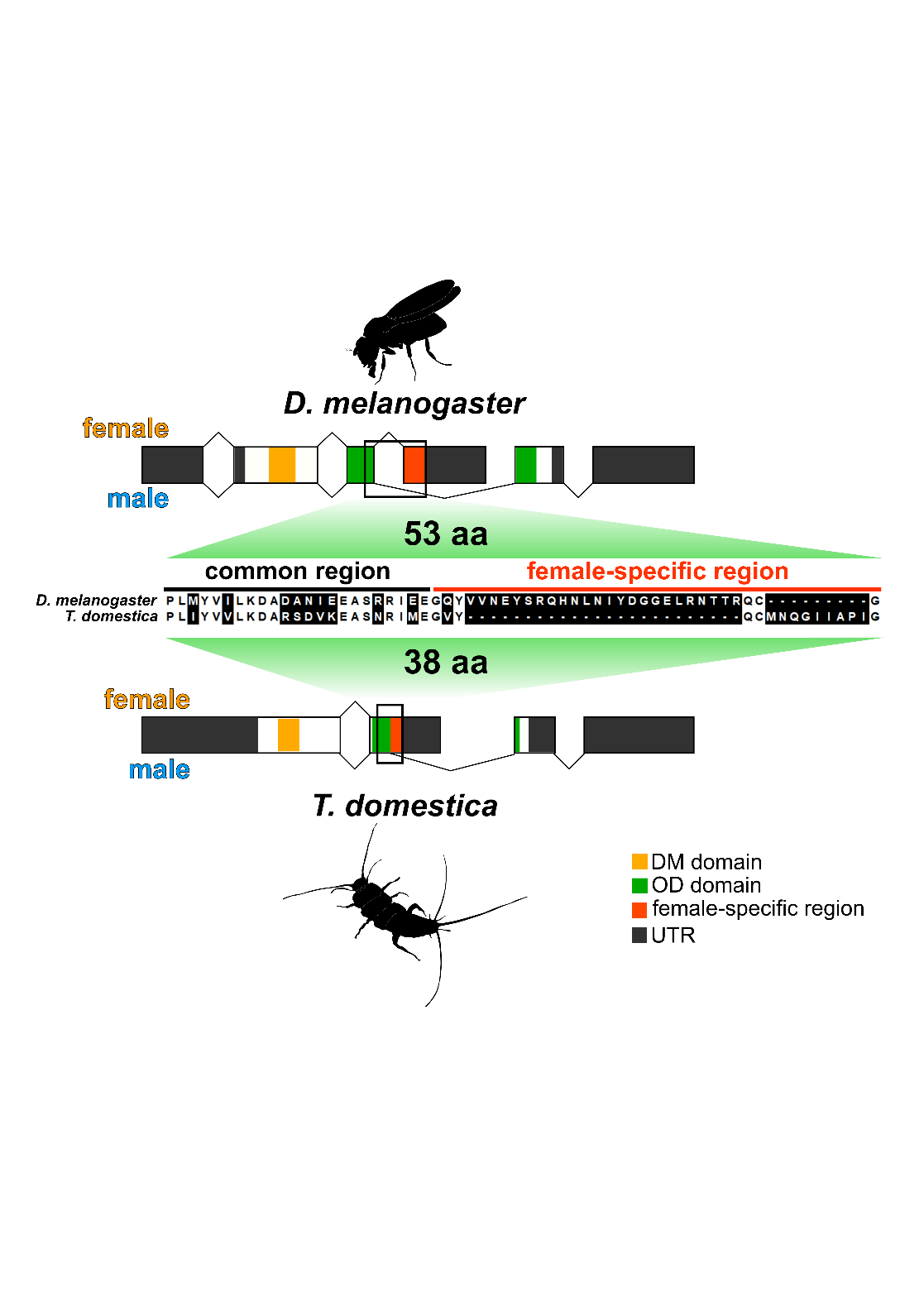
Supplementary FIG. 7.** Comparison of C-terminal sequences of *dsx* female-type between *Drosophila melanogaster* and *Thermobia domestica*. The upper schematic figure shows the gene structure of *dsx* in *D. melanogaster*. The lower schematic figure indicates the gene structure of *dsx* in *T. domestica*. The female-specific region is shown by the orange color. The middle image is the result of the multiple sequence alignment (MSA) of C-terminal region between two species. The MSA was performed using the MAFFT software. The white background indicates the matched residues between the species in the MSA. The female-specific region is much shorter in *T. domestica* (38 aa) than in *D. melanogaster* (53 aa).


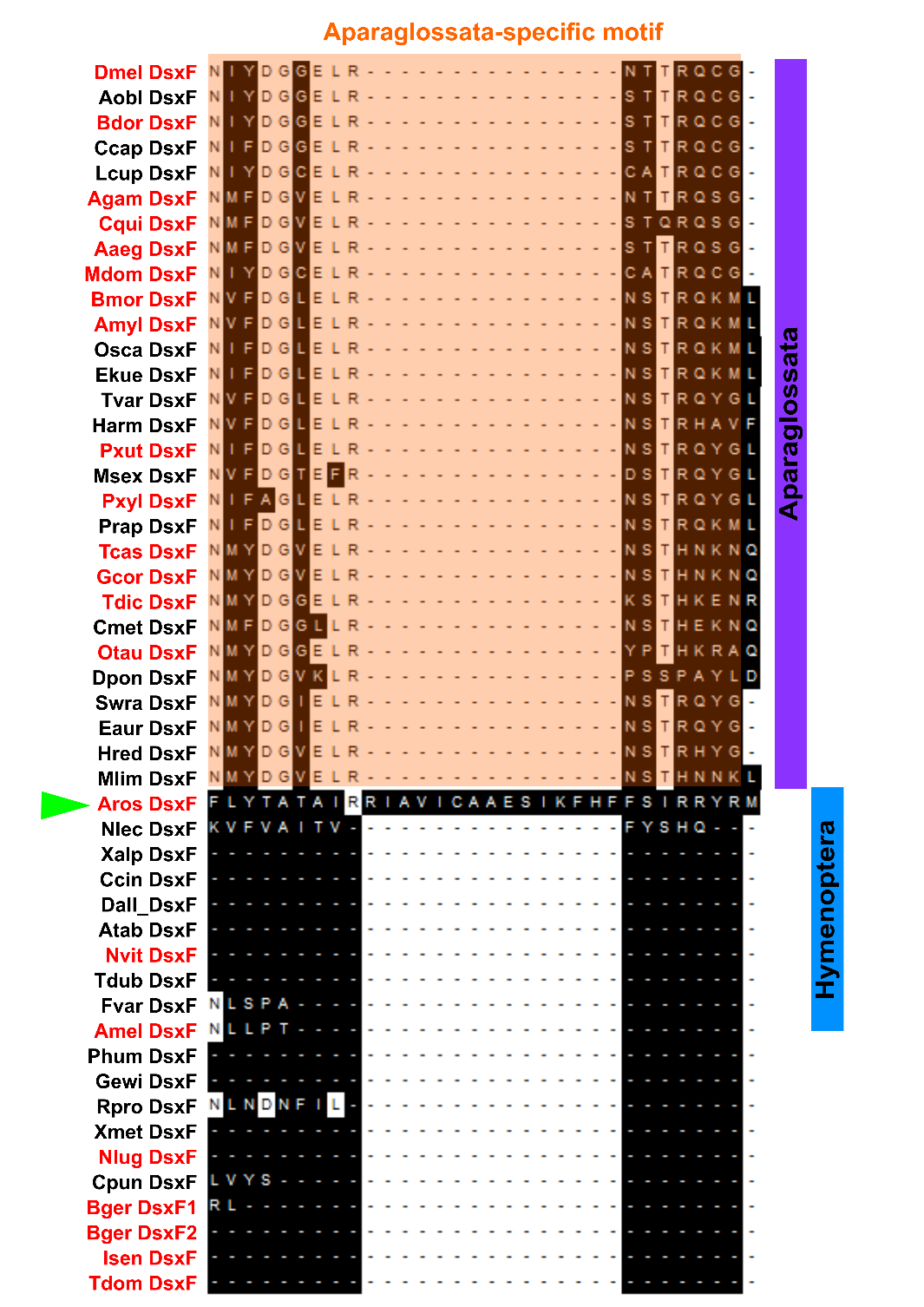
**Supplementary FIG. 8.** Multiple sequence alignments of the Aparaglossata-specific motif. The multiple sequence alignment of the C-terminal sequences was performed by the MAFFT software. This image shows the region around the Aparaglossata-specific motif indicated by the orange color. The species in the sequence name can be obtained from the supplementary table 8. The names indicated by the red color show species in which the functional analysis of *dsx* has been performed. The green arrowhead exhibits the sequence of *Athalia rosae* that has the amino acid sequences corresponding to the motif. The full result of the multiple sequence alignment can be obtained from the supplementary sequence file 3 (FASTA format).

**
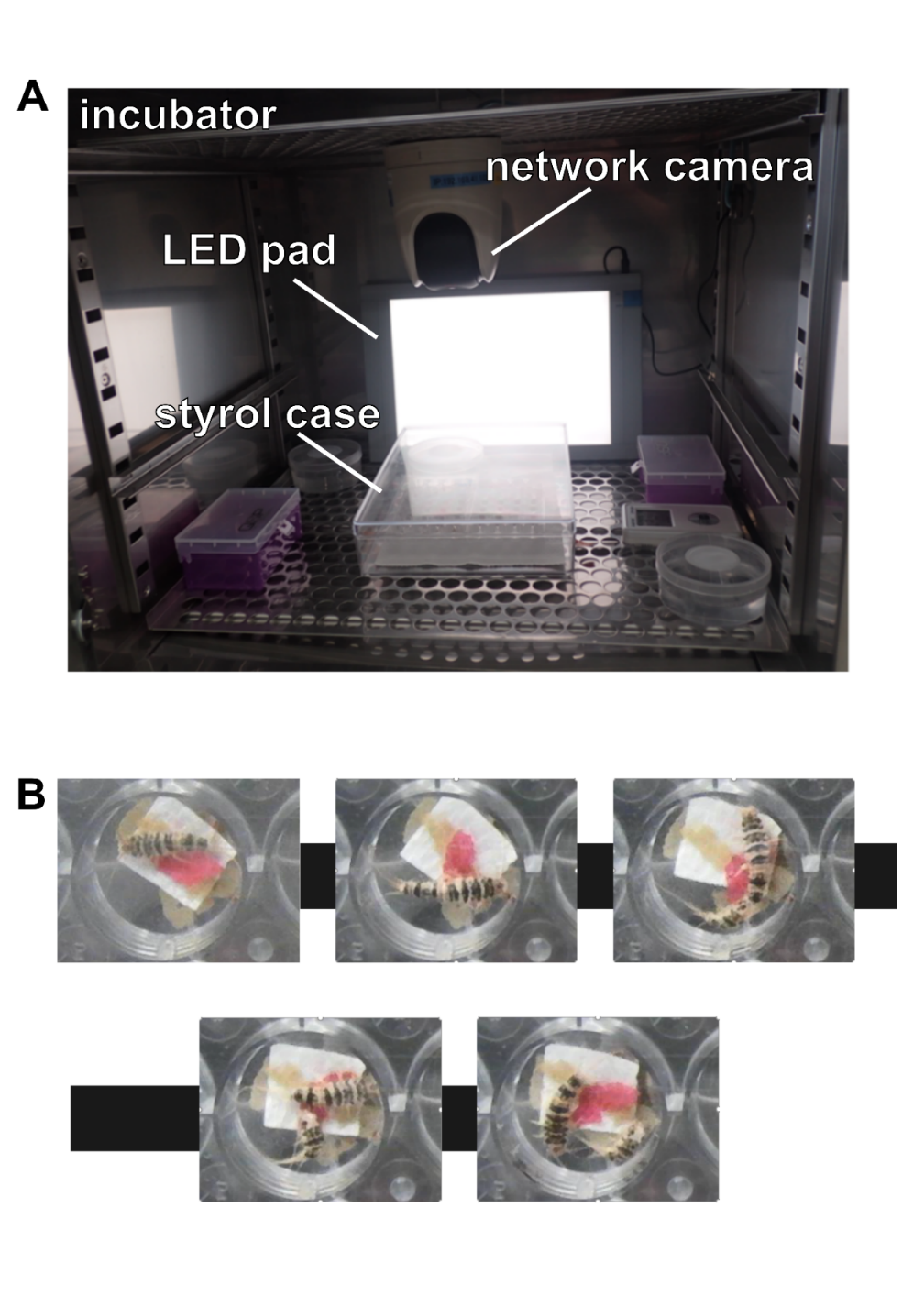
Supplementary FIG. 9.** Time-lapse imaging system. (*A*) A photo of the time-lapse imaging system used to observe the molt of *T. domestica*. The network camera is located on the floor of the incubator. The insects are put on 24-well plates in the styrol case. The LED pad is used for lighting up the inside of the incubator. The temperature within the incubator is kept at 37°C. (*B*) The time-lapse images during the molt. We set the interval of taking a photo every 5 minutes. The photos are ordered along with the time course from upper-right to lower-left. The firebrat proceeded its molting for 10 minutes.

**
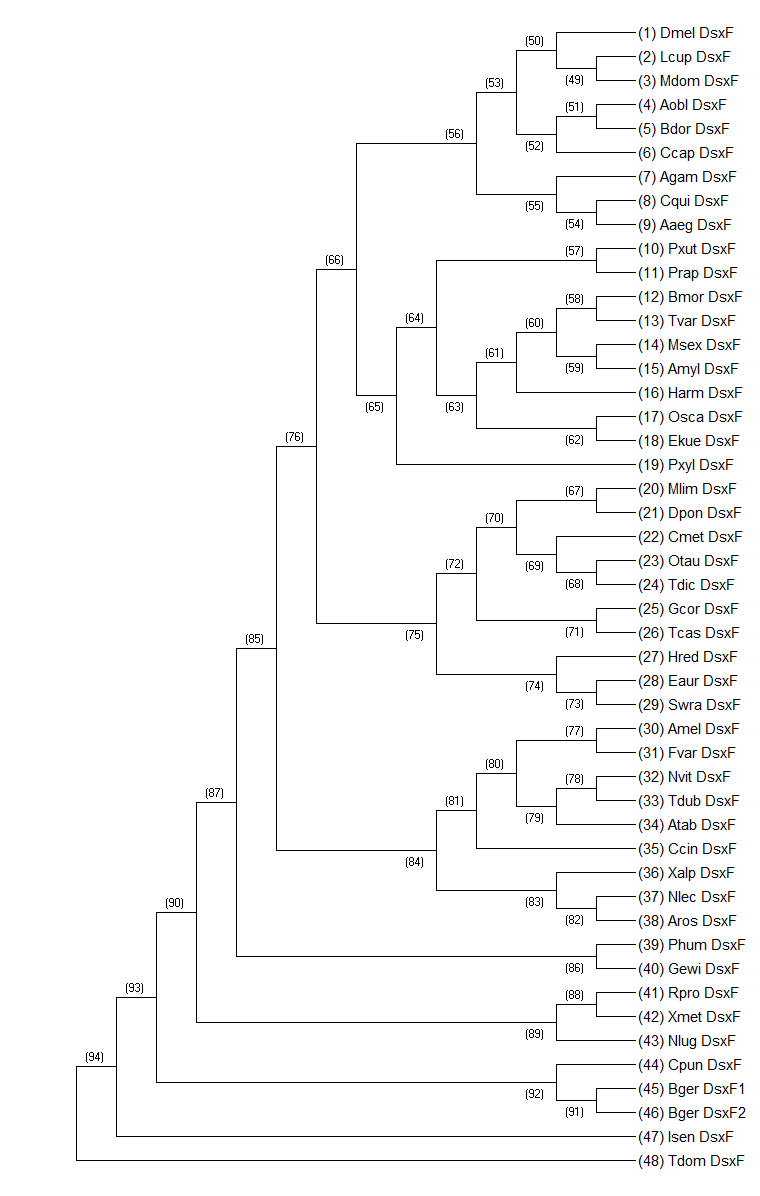
Supplementary FIG. 10.** The guide tree used for the ancestral sequence reconstruction. The tree topology was reconstructed based on previous phylogenetic studies (Wiegmann et al. 2011; Misof et al. 2014; Li et al. 2017; Peters et al. 2017; Zhang et al. 2018; Kawahara et al. 2019; McKenna et al., 2019; Gustafson et al. 2020). The topology is here: “(((((((((((Dmel_DsxF,(Lcup_DsxF,Mdom_DsxF)),((Aobl_DsxF,Bdor_DsxF),Ccap_DsxF)),(Agam_DsxF,(Cqui_DsxF,Aaeg_DsxF))),(((Pxut_DsxF,Prap_DsxF),((((Bmor_DsxF,Tvar_DsxF),(Msex_DsxF,Amyl_DsxF)),Harm_DsxF),(Osca_DsxF,Ekue_DsxF))),Pxyl_DsxF)),((((Mlim_DsxF,Dpon_DsxF),(Cmet_DsxF,(Otau_DsxF,Tdic_DsxF))),(Gcor_DsxF,Tcas_DsxF)),(Hred_DsxF,(Eaur_DsxF,(Swra_DsxF,Ains_DsxF))))),((((Amel_DsxF,Fvar_DsxF),((Nvit_DsxF,Tdub_DsxF),Atab_DsxF)),Ccin_DsxF),(Xalp_DsxF,(Nlec_DsxF,Aros_DsxF)))),(Phum_DsxF,Gewi_DsxF)),((Rpro_DsxF,Xmet_DsxF),Nlug_DsxF)),(Cpun_DsxF,(Bger_DsxF1,Bger_DsxF2))),Isen_DsxF),Tdom_DsxF);”. The numbers on the nodes indicate the operational taxon units (OTUs) and hypothetical taxon units (HTUs) matching with the numbers of taxon names in supplementary table 9. The OTU names can be referred to in supplementary table 8.


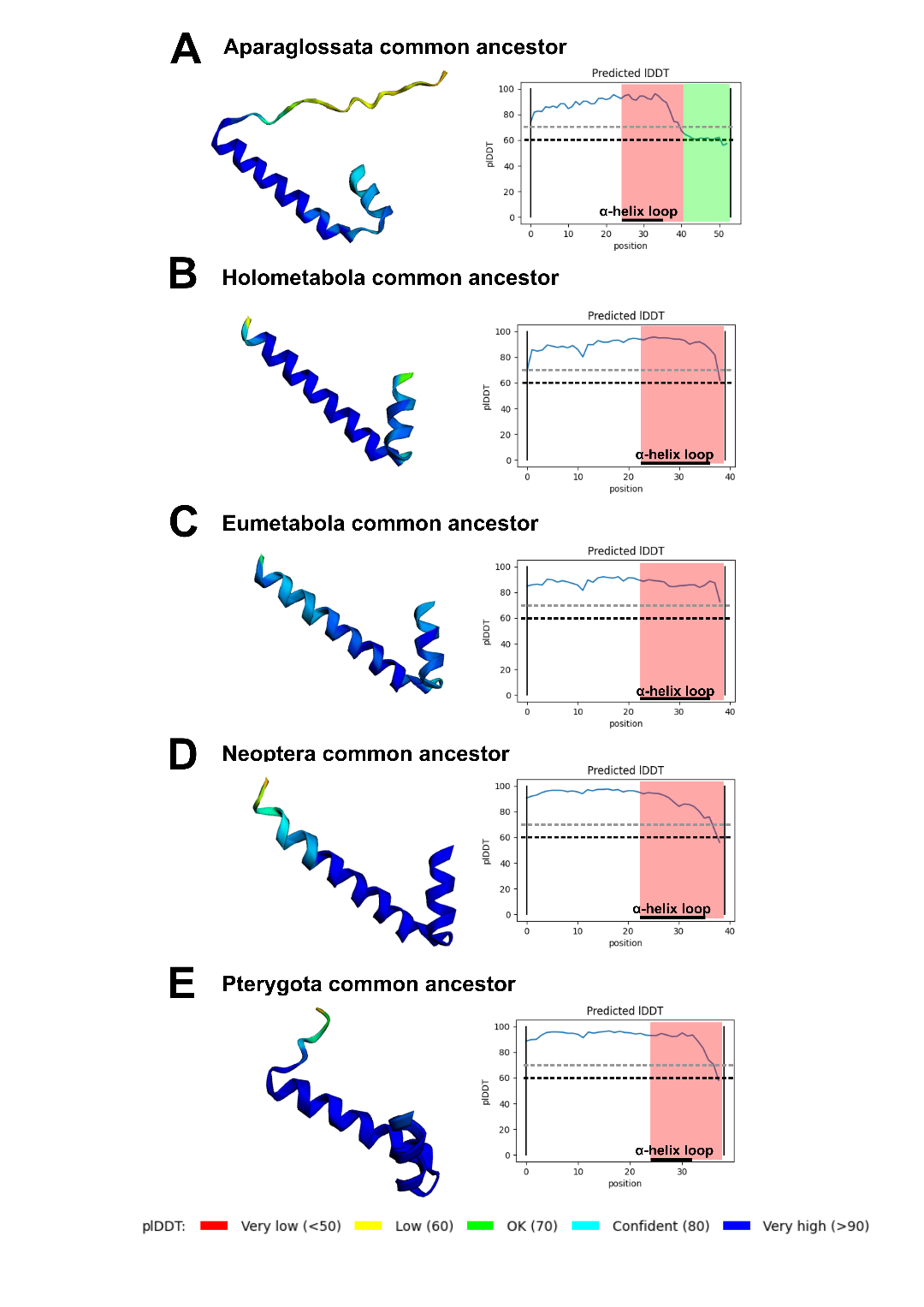
**Supplementary FIG. 11.** Accuracy of structure predictions of *dsx* female-type. Results of prediction of *dsx* female-type structure in the common ancestor of Aparaglossata (*A*), the common ancestor of Holometabola (*B*), the common ancestor of Eumetabola (*C*), the common ancestor of Neoptera (*D*), and the common ancestor of Aparaglossata (*D*). In each panel, the right 3D model shows the predicted structure of *dsx* female-type colored by its predicted local distance difference test (plDDT) score. The legend of color in the 3D model is shown at the bottom of the figure. The left graph indicates the plDDT score in each residue. The female-specific region is shown by red background. The Aparaglossata-specific region is colored by green. The black bar at the bottom of each graph shows the region predicted as an α-helix loop in the female-specific region. The black and gray dotted lines indicate plDDT = 60 and 70.

**Captions of Supplementary Tables**

**Supplementary Table 1.** Supplementary Table 1. Taxa and proteins used for the initial molecular phylogenetic analysis of DMRT family. Orange color indicates excluded species in the re-analysis.

**Supplementary Table 2.** Taxa and proteins used for the molecular phylogenetic analysis of DMRT family after excluding the problematic sequences.

**Supplementary Table 3.** Results of RT-qPCR assay and Brunner–Munzel test.

**Supplementary Table 4.** Results of Smirnov–Grubbs' test for expression level of *dsx* mRNA in nymphal RNAi males.

**Supplementary Table 5.** Results of generalized linear model of female traits.

**Supplementary Table 6.** Results of generalized linear model of male traits.

**Supplementary Table 7.** The expression level of *vitellogenin* genes in *Thermobia domestica*.

**Supplementary Table 8.** The taxa list used for the ancestral sequence reconstruction of *dsx*.

**Supplementary Table 9.** The result of the ancestral sequence reconstruction of the C-terminal region of *dsx* female-type.

**Supplementary Table 10.** Primers’ list used in this study.

**Supplementary Table 11.** Probabilities of reconstructed ancestral sequences of *dsx* female-type.

**Supplementary sequence**

**Supplementary Sequence 1**

>Aage_Dsx-like

PRAPPNCARCRNH--RLKIELKGHKRYCKYR-YCNCEKCRLTADRQRVMALQTALRRAQAQ----------DE-AR--------------------------------------------------------ARAA

>Harm_Dsx

PRAPPNCARCRNH--RLKIELKGHKRYCKYR-NCTCEKCRLTADRQRVMALQTALRRAQAQ----------DE-AR--------------------------------------------------------AR-A

>Bmor_Dsx

PRAPPNCARCRNH--RLKIELKGHKRYCKYQ-HCTCEKCRLTADRQRVMAKQTAIRRAQAQ----------DE-AR--------------------------------------------------------AR-A

>Ekue_Dsx

PRAPPNCARCRNH--RLKIELKGHKRYCKYR-NCMCEKCRLTADRQRVMALQTALRRAQAQ----------DE-AR--------------------------------------------------------AR--

>Msex_Dsx

PRAPPNCARCRNH--RLKIELKGHKRYCKYR-YCTCEKCRLTADRQRVMAMQTAMRRAQAQ----------DE-AR--------------------------------------------------------AR-A

>Ofur_Dsx

PRAPPNCARCRNH--RLKVELKGHKRECKYR-YCTCEKCRLTADRQRVMALQTALRRAQAQ----------DE-AR--------------------------------------------------------AR-S

>Osca_Dsx

PRAPPNCARCRNH--RLKVELKGHKRYCKYR-YCTCEKCRLTADRQRVMALQTALRRAQAQ----------DE-AR--------------------------------------------------------AR-S

>Paeg_Dsx

PRAPPNCARCRNH--RLKIELKGHKRYCKYR-YCTCEKCRLTADRQRVMALQTALRRAQAQ----------DE-AR--------------------------------------------------------AR-A

>Pmac_Dsx

PRAPPNCARCRNH--RLKVELKGHKRYCKYR-YCTCEKCRLTADRQRVMAMQTALRRAQAQ----------DE-AR--------------------------------------------------------AR-A

>Pxyl_Dsx

PRAPPNCARCRNH--RLKVELKGHKRYCKYR-YCNCEKCRLTADRQRVMALQTALRRAQAQ----------DE-AR--------------------------------------------------------AR-A

>Obru_Dsx

PRAPPNCARCRNH--RLKVELKGHKRYCKYR-YCTCEKCRLTADRQRVMALQTALRRAQAQ----------DE-AR--------------------------------------------------------AR-H

>Rnub_DM

PRTPPNCARCRNH--RLKIALKGHKRYCKYR-FCNCDKCRLTAERQRVMALQTALRRAQAQ----------DE-AR--------------------------------------------------------AR-V

>Amel_Dsx

PRA-RNCARCLNH--RLEITLKSHKRYCKYR-TCTCEKCKITANRQQVMRQNMKLKRHLAQ----------DK-VK--------------------------------------------------------VR-V

>Bpyr_Dsx

PRAPRNCARCRNH--RLKITLKSHKRYCKYR-YCNCEKCKITADRQRVMAQQTKLRRQLAQ----------DE-VK--------------------------------------------------------VR-A

>Emex_Dsx

PRAPRNCARCRNH--RLKITLKSHKRYCKYR-YCNCEKCKITADRQRVMAQQTKLRRQLAQ----------DE-VK--------------------------------------------------------VR-A

>Fvar_Dmrt1

PRAPRNCQRCRNH--RLKITVKSHKRYCKYR-FCECDRCKVTADKQRVTAMQAKLRRQLAQ----------DE-VK--------------------------------------------------------VR-V

>Cgig_Dsx

PRTPPNCARCRNH--RLKIPLKGHKRYCKYR-YCKCEKCKLTADRQRVMAKQTALRRALAQ----------DE-VK--------------------------------------------------------IR-A

>Olig_Dsx

PRKPPNCARCRNH--QLKIAVKGHKRYCRYR-YCKCDSCKLTADRQRVMARQTALRRALAL----------DE-VR--------------------------------------------------------VR-A

>Aros_Dsx

PRTPPNCARCRNH--RLKIALKGHKRYCKYR-SCNCEKCRLTAERQRVMALQTALRRAQAQ----------DE-AR--------------------------------------------------------VR-G

>Xalp_Dsx

PRTPPNCARCRNH--RLKIALKGHKRYCKYR-YCNCEKCRLTAERQRVMALQTALRRAQAQ----------DE-AR--------------------------------------------------------VR-G

>Nlec_Dsx

PRTPPNCARCRNH--RLKIALKGHKRYCKYR-SCNCEKCRLTAERQRVMALQTALRRAQAQ----------DE-AR--------------------------------------------------------VR-G

>Bdor_Dsx

PRTPPNCARCRNH--GLKITLKGHKRYCKFR-FCTCEKCRLTADRQRVMALQTALRRAQAQ----------DE-QR--------------------------------------------------------VL-Q

>Btry_Dsx

PRTPPNCARCRNH--GLKITLKGHKRYCKFR-FCTCEKCRLTADRQRVMALQTALRRAQAQ----------DE-QR--------------------------------------------------------VL-Q

>Ccap_Dsx

PRTPPNCARCRNH--GLKITLKGHKRYCKFR-YCTCEKCRLTADRQRVMALQTALRRAQAQ----------DE-QR--------------------------------------------------------VL-Q

>Hpal_DM

PRTPPNCARCRNH--NFKIGLKGHKRYCRFR-SCTCSKCCLTAERQRVMALQTALRRAQAQ----------DE-KY--------------------------------------------------------YE-Q

>Icra_Dsx

PRTPPNCARCRNH--RVKIPLKGHKRYCKYR-TCNCQKCRLTAERQRVMAMQTALRRAQAQ----------DE-AM--------------------------------------------------------LH-A

>Xant_Dsx

PRTPPNCARCRNH--RVKIPLKGHKRYCKYR-TCNCQKCRLTAERQRVMAMQTALRRAQAQ----------DE-AM--------------------------------------------------------LH-A

>Lmig_Dsx

QRTPPNCARCRNH--GYKIPLKGHKRYCKYR-YHTCDKCLLTAERQRVMAMQTALRRAQAQ----------DE-AM--------------------------------------------------------GL-K

>Tsub_Dsx

QRTPPNCARCRNH--GLKIPLKGHKRYCKYR-YHDCDKCLLTAERQRVMALQTALRRAQAQ----------DE-AL--------------------------------------------------------AA-S

>Kbie_DM

PRTPPNCARCRNH--RLKIGLKGHKRYCKFR-SCKCEKCCLTAERQRVMAAQTALRRAQAQ----------DE-QH--------------------------------------------------------LT-S

>Tgla_DM

PRTPPNCARCRNH--RLKIGLKGHKRYCKFR-YCTCDKCRLTAERQRVMAAQTALRRAQAQ----------DE-KY--------------------------------------------------------LS-S

>Mext_Dsx

PRTPPNCARCRNH--RLKIGLKGHKRYCKYR-YCTCSKCRLTAERQRVMALQTALRRAQAQ----------DE-KY--------------------------------------------------------LA-Q

>Mfas_Dsx

PRTPPNCARCRNH--GLKITLKGHKRYCKYR-YCTCEKCRLTADRQRVMALQTALRRAQAQ----------DE-QR--------------------------------------------------------AL-Q

>Dmel_Dsx

PRTPPNCARCRNH--GLKITLKGHKRYCKFR-YCTCEKCRLTADRQRVMALQTALRRAQAQ----------DE-QR--------------------------------------------------------AL-H

>Epen_Dsx

PRTPPNCARCRNH--RLKIGLKGHKRYCKYR-YCNCDKCCLTAERQRVMALQTALRRAQAQ----------DE-AR--------------------------------------------------------QQ-H

>Mrel_Dsx

PRTPPNCARCRNH--RLKIGLKGHKRYCKYR-YCNCDKCCLTAERQRVMALQTALRRAQAQ----------DE-AR--------------------------------------------------------QQ-H

>Gnip_DM

PRTPPNCARCRNH--RLKIGLKGHKRYCKYR-HCTCDKCCLTAERQRVMAMQTALRRAQAQ----------DE-TL--------------------------------------------------------AT-Q

>Gryl_DM

PRTPPNCARCRNH--RLKIGLKGHKRYCKYR-ACTCDKCCLTAERQRVMAMQTALRRAQAQ----------DE-TL--------------------------------------------------------AT-Q

>Cpun_Dsx

PRTPPNCARCRNH--RLKIGLKGHKRYCKYR-YCNCDKCCLTAERQRVMALQTALRRAQAQ----------DE-AR--------------------------------------------------------MA-T

>Bger_Dsx

PRTPPNCARCRNH--RLKIGLKGHKRYCTFR-SCVCEKCVLTAERQRVMALQTALRRAQAQ----------DE-AR--------------------------------------------------------ER-Q

>Tdom_Dsx

ART-PKCARCRNH--RLKIPLKGHKRYCKYR-FCNCDKCLLTAERQRVMALQTALRRAQAQ----------DE-AR--------------------------------------------------------AA--

>Gcor_Dsx

PRTPPNCARCRNH--RMKIALKGHKRYCKYR-TCKCEKCRLTSERQRVMAMQTALRRAQAQ----------DE-AM--------------------------------------------------------MR-N

>Tcas_Dsx

PRTPPNCARCRNH--RLKIALKGHKRYCKYR-TCKCEKCRLTTERQRVMAMQTALRRAQAQ----------DE-AM--------------------------------------------------------LR-S

>Otau_Dsx

PRTPPNCARCRNH--RVKVPLKGHKRYCKYR-HCKCEKCRLTSERQRVMAMQTALRRAQAQ----------DE-AM--------------------------------------------------------LR-Q

>Tdic_Dsx

PRTPPNCARCRNH--RLKIALKGHKRYCKYR-HCKCEKCRLTSERQRVMAMQTALRRAQAQ----------DE-AM--------------------------------------------------------LR-Q

>Mviol_Dsx

PRTPPNCARCRNH--RMKVALKGHKRYCKFR-TCKCEKCRLTSERQRVMAMQTALRRAQAQ----------DE-AM--------------------------------------------------------MK-T

>Hydr_Dsx

PRTPPNCARCRNH--RLKIALKGHKRYCKFR-HCNCDRCRLTAERQRVMALQTALRRAQAQ----------DE-AR--------------------------------------------------------AK-A

>Ctos_DM

PRTPPNCARCRNH--LLKIALKGHKRFCRYR-YCDCDKCRLTAERQRVMAAQTALRRNQAQ----------DE-AI--------------------------------------------------------MA-A

>Same_DM

PRTPPNCARCRNH--LLKIALKGHKRFCRYR-YCDCDKCCLTAERQRVMAAQTALRRNQAQ----------DE-AI--------------------------------------------------------MA-N

>Ptap_DM

PRTPPNCARCRNH--LLKIALKGHKRFCRYR-DCNCDKCLLTLERQRVMAAQTALRRNQAQ----------DE-AL--------------------------------------------------------MA-T

>Annu_Dsx

PRTPPNCARCRNH--RLKIALKGHKRYCKYR-YCNCDKCRLTAERQRVMALQTALRRAQAQ----------DE-AR--------------------------------------------------------AR-S

>Ppra_Dsx

PRTPPNCARCRNH--RLKIPLRGHKRYCRFR-NCICHKCKLTAERQRVMAMQTALRRAQAQ----------DE-AM--------------------------------------------------------QT-S

>Bhye_Dsx

PRTPPNCARCRNH--RLKIALKGHKRYCKFR-NCNCYKCILTAERQRIMAVQTAQRRAQAQ----------DE-AR--------------------------------------------------------EA-K

>Ccor_Dsx

PRTRPNCARCRNH--RVKVPLKGHKRYCKYR-TCSCQKCCLTAERQRVMAMQTALRRAQAQ----------DE-AM--------------------------------------------------------LN-S

>Lcup_Dsx

PRTKPNCARCHNH--GFKIKLKGHKRYCKYR-NCNCEKCRLTADRQRVMALQTALRRAQQQ----------DE-QR--------------------------------------------------------IL-Q

>Mdom_Dsx

PRTKPNCARCHNH--GLKIKLKGHKRYCKYR-FCNCEKCRLTADRQRVMALQTALRRAQQQ----------DE-AR--------------------------------------------------------IL-Q

>Auri_DM

PRTPPNCARCRNH--SIKIILKGHKRYCRFR-DCYCSKCLLTAERQRVMAAQTQLRRAQAQ----------DE-KY--------------------------------------------------------IQ-E

>Pcat_DM

SRTAPNCARCRNH--GLKIELKGHRRYCKYR-SCGCGKCSLTAARQRIMASQTALRRAQAQ----------DE-RY--------------------------------------------------------QQ-I

>Acha_DM

KRKTPNCARCRNH--YVKIKLKGHKRYCKYR-NCRCNKCTLTAERQRIMAAQTAIRRSQAQ----------DE-ER--------------------------------------------------------IE-Q

>Dipl_DM

HRKAPNCARCRNH--YLKIELKGHKRYCKFR-FCRCDKCTLTAERQRVMALQTALRRGQAQ----------DE-QR--------------------------------------------------------MA-R

>Faur_DM

HRKAPNCARCRNH--YLKMGLKGHKRYCKFR-YCECSKCRLTYERQRVMALQTALRRGQAQ----------DE-QR--------------------------------------------------------LA-R

>Faur_DM2

HRKAPNCAKCRNH--SLNNGLKGHKRYCKFR-FCQCAKCRLTVQRQRVMAKQTAVRRAQAQ----------DE-QR--------------------------------------------------------EG-Q

>Gmar_DM

QRKAPKCARCRNH--YLKIGLKGHKRYCKYR-FCQCKKCELTAERQRVMAQQTALRRGQAQ----------DE-IR--------------------------------------------------------LA-R

>Gmar_DM2

QRTAPNCARCRNH--YLTIELKGHKRYCKFR-YCHCFKCQLTAKRQRVMAQQTALRRSQAQ----------DE-KR--------------------------------------------------------L---

>Mmol_DM

PRTPPNCARCRNH--QLKIPLKGHKRYCRYR-SCECGKCRLTSERQRVMAMQTALRRAQAQ----------DE-IY--------------------------------------------------------YQ-A

>Smel_DM

PRTPPNCARCRNH--LLKIPLKGHKRYCRYR-TCACNKCCLTSERQRVMAMQTALRRAQAQ----------DE-MF--------------------------------------------------------FQ-T

>Xves_DM

PRTPPNCARCRNH--LLKIPLKGHKRYCRYR-TCACNKCCLTSERQRVMAMQTALRRAQAQ----------DE-MF--------------------------------------------------------FQ-N

>Pcat_DM2

SLTDSKCARCRNH--NMKVELKGHKRYCKYR-SCGCVKCSLTAERQTIIALQLAVRRTQAQ----------DE-RY--------------------------------------------------------RQIQ

>Aasp_Dsx-like

NSMNRLCALCRNH--GLKIPVKGHKRFCGYR-LCLCKECCLVKERQRVVAMHLYFRRAQEQ----------EE-AD--------------------------------------------------------RG-S

>Mext_Dsxlike

NGMNRLCALCRNH--GLKIPVKGHKRFCGYR-LCLCKECCLVKERQRVVAMHLYFRRAQEQ----------EE-AD--------------------------------------------------------RG-T

>Choo_Dsxlike

NSINRLCALCRNH--GLKIPVKGHKRYCGYR-LCLCKECCLVKERQRVVAMHLYFRRAQEQ----------EE-AD--------------------------------------------------------RG-D

>Psch_Dsxlike

NNMNRLCALCRNH--GLKMPVKGHKRFCGYR-LCLCKECCLVKERQRVVAMHLYFRRAQEQ----------EE-SD--------------------------------------------------------KG-A

>Tcri_Dsx-like

NNLNRLCALCRNH--GLKKPVKGHKRFCAYK-LCVCRECCLVKERQRVVALHLYYRRAQDQ----------EE-ND--------------------------------------------------------RK-P

>Tdom_Dsx-like

VGKTPSCAQCRNH--GLRVPVKGHKRYCAYK-LCTCKECCLVKERQRVVALHLYVRRAQDQ----------EE-AD--------------------------------------------------------SS-Q

>Tger_Dsx-like

KIPSRLCAQCRNH--GLRVPVKGHKRFCAYK-LCHCAECLLVKERQRVVALHLYIRRAQDQ----------EE-AD--------------------------------------------------------SS-Q

>Edan_Dsxlike

TSSNRLCAQCRNH--GLRVPVRGHKRYCTFR-LCTCRECRLVRERQRVVALHLYVRRAQEQ----------EE-EA--------------------------------------------------------AE-A

>Eins_Dsxlike

QGSNRLCAQCRNH--GLRVPVRGHKRYCTFR-LCSCRDCRLVRERQRVVALHLYVRRAQEQ----------EE-EA--------------------------------------------------------AA-A

>Eury_Dsx2

KTASRLCAFCRNH--SLKIPVKGHKRFCRNR-TCNCAECKLVRERQRVVALHLYVRRAQEQ----------EE-EA--------------------------------------------------------AA-A

>Ibic_Dsxlike

QGSNRLCAQCRNH--GLRVPVRGHKRYCTYR-LCTCRECRLVRERQRIVALHLYVRRAQEQ----------EE-EA--------------------------------------------------------AA-A

>Baet_Dsx

ALSNRMCAQCQNH--GLKIPVRGHKRFCKYR-LCNCQNCLLVKERQRIVALHLYVRRAQQQ----------EE-DA--------------------------------------------------------AE-A

>Cdip_DM3

PVSKRMCAQCQNH--GLKEPVRGHKRHCKYR-LCNCKNCLLVKERQRIVALHLYVRRAQQQ----------EE-DA--------------------------------------------------------AE-A

>Afus_B

SPRTPKCARCRNH--KKSIPVKGHKRYCEFR-SCTCEKCCLTAERQRVMALQVAVRRAQAQ----------DE-AR--------------------------------------------------------AA-Q

>Enos_Dsx

PRTPPNCARCRNH--RLKIPLRGHKRYCRFR-TCTCEKCRLTAERQRVMAMQTALRRAQAQ----------DE-AM--------------------------------------------------------LS-A

>Esup_Dsx

ARTPPKCARCRNH--RLKIPLKGHKRYCKFR-YCKCDKCRLTAERQRVMAMQTALRRAQAQ----------DE-AN--------------------------------------------------------RG-M

>Isen_Dsx

ARTPPKCARCRNH--LLKIALKGHKRYCKYR-YCKCDKCRLTAERQRVMAMQTALRRAQAQ----------DE-AN--------------------------------------------------------RG-M

>Lful_Dsx

ARTPPKCARCRNH--RLKIPLKGHKRYCKFR-YCKCDKCRLTAERQRVMAMQTALRRAQAQ----------DE-AN--------------------------------------------------------RG-L

>Cfel_Dsx

PRTPPNCARCRNH--RLKIPLKGHKRYCRYL-YCKCEKCRLTADRQRDMARQTAMRRAQAQ----------DE-AR--------------------------------------------------------GL-S

>Cgal_Dsx

PRTPPNCARCRNH--RLKIALKGHKRYCRFL-YCKCEKCKLTADRQRVMAKQTALRRAQAQ----------DE-AR--------------------------------------------------------GL-S

>Atub_Dsx

PRTKPNCARCGNH--HLKMRLRGHKRYCKYR-YCTCEKCILTTDRQKVMALQTALRRAQEQ----------DA-TR--------------------------------------------------------EE-P

>Tpal_DmrtA2

ARTPPNCALCRNH--RLKIGLKGHKRYCKYR-YCDCDKCRLTAERRRVMALQTALRRAQAQ----------DE-AR--------------------------------------------------------HP-V

>Edan_Dsx

ARTPPKCARCRNH--RLKIPLKGHKRYCKYR-FCNCYKCTLTADRQRVMAEQTALRRAQAQ----------DE-AF--------------------------------------------------------AA-C

>Focc_Dsx

ARTPPNCALCRNH--RLKIGLKGHKRYCKYR-YCDCDKCQLTAERRRVMALQTALRRAQAQ----------DE-QR--------------------------------------------------------QP-N

>Afus_A

ILRTPKCARCRNH--GVISCLRGHKKMCRWK-ECICEMCELVAERQRIMAAQVALRRQQIG----------QK-LK--------------------------------------------------------ET-N

>Afus_C

YQRTPKCARCRNH--GVVSALKGHKRYCRWR-DCVCAKCTLIAERQRVMAAQVALRRQQAQ----------EE-NE--------------------------------------------------------AR-E

>Annu_Dmrt99B

YQRTPKCARCRNH--GVVSALKGHKRYCRWR-DCVCAKCTLIAERQRVMAAQVALRRQQAQ----------EE-SE--------------------------------------------------------AR-E

>Btab_DmrtA2-2

YQRTPKCARCRNH--GVVSALKGHKRYCRWR-DCVCAKCTLIAERQRVMAAQVALRRQQAQ----------EE-NE--------------------------------------------------------AR-E

>Caqu_DM1

YQRTPKCARCRNH--GVVSALKGHKRYCRWR-DCVCAKCTLIAERQRVMAAQVALRRQQAQ----------EE-NE--------------------------------------------------------AR-E

>Tdom_Dmrt99B

YQRTPKCARCRNH--GVVSALKGHKRYCRWR-DCVCAKCTLIAERQRVMAAQVALRRQQAQ----------EE-NE--------------------------------------------------------AR-E

>Clec_DmrtA2-like

YQRTPKCARCRNH--GVVSALKGHKRYCRWR-DCVCAKCTLIAERQRVMAAQVALRRQQAQ----------EE-SE--------------------------------------------------------AR-E

>Gbue_DM1

YQRTPKCARCRNH--GVVSALKGHKRYCRWR-DCVCAKCTLIAERQRVMAAQVALRRQQAQ----------EE-NE--------------------------------------------------------AR-E

>Mext_Dmrt99B

YQRTPKCARCRNH--GVVSALKGHKRYCRWR-DCACAKCTLIAERQRVMAAQVALRRQQAQ----------EE-SE--------------------------------------------------------AR-E

>Lstri_DM

YQRTPKCARCRNH--GVVSALKGHKRYCRWR-DCMCAKCTLIAERQRVMAAQVALRRQQAQ----------EE-SE--------------------------------------------------------AR-E

>Ocin_DmrtA2

YQRTPKCARCRNH--GVVSALKGHKRYCRWR-DCSCAKCNLIAERQRVMAAQVALRRQQAQ----------EE-NE--------------------------------------------------------AR-E

>Edan_Dmrt99B

YQRTPKCARCRNH--GVVSALKGHKRYCRWR-DCVCAKCTLIAERQRVMAAQVALRRQQAQ----------EE-NE--------------------------------------------------------AR-E

>Tpal_DmrtA2-like

YQRTPKCARCRNH--GVVSALKGHKRYCRWR-DCVCAKCTLIAERQRVMAAQVALRRQQAQ----------EE-SE--------------------------------------------------------AR-E

>Dmel_Dmrt99B

YQRTPKCARCRNH--GVVSALKGHKRYCRWR-DCVCAKCTLIAERQRVMAAQVALRRQQAQ----------EE-NE--------------------------------------------------------AR-E

>Lcup_Dmrt99B

YQRTPKCARCRNH--GVVSALKGHKRYCRWR-DCLCAKCTLIAERQRVMAAQVALRRQQAQ----------EE-NE--------------------------------------------------------AR-E

>Mdom_Dmrt99B

YQRTPKCARCRNH--GVVSALKGHKRYCRWR-DCLCAKCTLIAERQRVMAAQVALRRQQAQ----------EE-NE--------------------------------------------------------AR-E

>Tcas_Dmrt99B

YQRTPKCARCRNH--GVVSALKGHKRYCRWR-DCNCAKCTLIAERQRVMAAQVALRRQQAQ----------EE-NE--------------------------------------------------------AR-E

>Bmor_Dmrt99B

YQRTPKCARCRNH--GVVSALKGHKRYCRWR-DCVCAKCTLIAERQRVMAAQVALRRQQAQ----------EE-NE--------------------------------------------------------AR-E

>Esin_Dmrt-like

YQRTPKCARCRNH--GVVSALKGHKRYCRWR-DCNCAKCTLIAERQRVMAAQVALRRQQAQ----------EE-NE--------------------------------------------------------VR-E

>Spar_DM

YQRTPKCARCRNH--GVVSALKGHKRYCRWR-DCNCAKCTLIAERQRVMAAQVALRRQQAQ----------EE-NE--------------------------------------------------------VR-E

>Hazt_DM

FQRTPKCARCRNH--GVVSALKGHKRYCRFR-DCMCAKCTLIAERQRVMAAQVALRRQQAR----------EE-NE--------------------------------------------------------VR-D

>Mmol_DM2

YQRTPKCARCRNH--GVVSALKGHKRYCRWR-DCVCAKCTLIAERQRVMAAQVALRRQQAQ----------EE-NE--------------------------------------------------------AR-E

>Emex_DmertA2

YQRTPKCARCRNH--GVVSALKGHKRYCRWR-DCVCAKCTLIAERQRVMAAQVALRRQQAQ----------EE-SE--------------------------------------------------------AR-E

>Fvar_DmrtA2

YQRTPKCARCRNH--GVVSALKGHKRYCRWR-DCVCAKCTLIAERQRVMAAQVALRRQQAQ----------EE-SE--------------------------------------------------------AR-E

>Dmag_Dmrt99B

YQRTPKCARCRNH--GVVSALKGHKRYCRWR-DCACAKCTLIAERQRVMAAQVALRRQQAQ----------EE-NE--------------------------------------------------------AR-E

>Xlae_Dmrt5

YPRTPKCARCRNH--GVVSALKGHKRYCRWK-DCMCAKCTLIAERQRVMAAQVALRRQQAQ----------EE-NE--------------------------------------------------------AR-E

>Bmor_Dmrt11E

ALRTPKCARCRNH--GVISCLKGHKRLCRWR-DCRCPGCLLVLERQRVMAAQVALRRQQGA----------GG-PE--------------------------------------------------------SR-N

>Btau_Dmrt2

LSRTPKCARCRNH--GVVSCLKGHKRFCRWR-DCQCANCLLVVERQRVMAAQVALRRQQAT----------ED-KK--------------------------------------------------------GL-S

>Caqu_DM3

LLRTPKCARCRNH--GVISCLKGHKKLCRWR-ECQCPNCLLVVERQRVMAAQVALRRQQSS----------ED-NS--------------------------------------------------KDGSAKQK-T

>Ceut_Dmrt11E

LLRTPKCARCRNH--GVISCLKGHKRLCRWR-ECQCPNCQLVVERQRVMAAQVALRRQQSS----------EE-GQ---------------------------------------------------DVRSRAK-S

>Kbie_DM2

LLRTPKCARCRNH--GVISCLKGHKRLCRWR-ECQCPNCQLVVERQRVMAAQVALRRQQSS----------ED-GQ---------------------------------------------------DSRSRVQ-S

>Rvir_DM

LLRTPKCARCRNH--GVISCLKGHKRLCRWR-ECQCPNCQLVVERQRVMAAQVALRRQQSS----------ED-SD----------------------------------------------------DRARVQ-S

>Gdja_DM

LLRTPKCARCRNH--GVISCLKGHKRLCRWR-ECQCPNCQLVVERQRVMAAQVALRRQQSS----------EE-GQ---------------------------------------------------DVRSRVQ-T

>Tdom_Dmrt11E

LLRTPKCARCRNH--GVISCLKGHKKLCRWR-ECQCPNCLLVVERQRVMAAQVALRRQQSS----------ED-SK---------------------------------------------------DSRSRVQ-S

>Choo_Dmrt11E

LLRTPKCARCRNH--GVISCLKGHKRLCRWR-ECRCPNCQLVVERQRVMAAQVALRRQQSS----------ED-GP---------------------------------------------------ESHAKAQ-T

>Dmag_Dmrt11E

LLRTPKCARCRNH--GVVSCLKGHKKLCRWK-ECQCTNCLLVVERQRVMAAQVALRRQQNS----------ES-AKSDGQVKP--------------------------------------------NQSKVTK-S

>Sver_Dmrt11E

LLRTPKCARCRNH--GVVSCLKGHKKLCRWR-DCRCANCLLVVERQRVMAAQVALRRQQAS----------EH-KDQVSTTSPSGTTTTPSAPAAGEAPG----QRQDSPAT--------------VAARAKLK-S

>Zcau_DM2

LSRTPKCARCRNH--GVISCLKGHKKMCRWK-ECECSNCQLVVERQRIMAAQVALRRQQCT----------EE-KD----------------------------------------------------LGTRYK-I

>Edan_Dmrt11E

VLRTPKCARCRNH--GVISGLKGHKRLCRWR-ECRCPSCLLVVERQRVMAAQVALRRQQSS----------EE-GG------------------------------NSGP----------------CRPGVIPR-S

>Lcup_Dmrt11E

MLRTPKCARCRNH--GVISCVKGHKKLCRWR-ECTCPNCQLVVDRQRVMAAQVALRRQQTM----------EE-PYETNSDDSS------------------SLPNCTSP----------------QNTSVLKR-T

>Mdom_Dmrt11E

MLRTPKCARCRNH--GVISCVKGHKKLCRWR-ECTCQNCQLVVDRQRVMAAQVALRRQQTTLA----DENNSN-GYETHSDDSN------------------SSQSCHQPAA-------------AAASAVYQR-T

>Drer_Dmrt2

LSRTPKCARCRNH--GVVSCLKGHKRFCRWR-DCQCANCLLVVERQRVMAAQVALRRQQAT----------ED-KK--------------------------------------------------------GI-T

>Eury_Dmrt11E

VLRSPKCARCRNH--GVISSLKGHKRLCRWR-ECRCPSCLLVVERQRVMAAQVALRRQHCN----------SE-KK------------------------------CSSGAE--------------NKMSCASR-S

>Dmel_Dmrt11E

LLRTPKCARCRNH--GVISCVKGHKRLCRWR-ECCCPNCQLVVDRQRVMAAQVALRRQQTM----------EA-LEATASSTKSTGVTTASANNSSSSEGEDSLSSTSPPPAHSHPHSHSHPTSCVSNSSSSSA-T

>Nlug_Dsx-like3

SGRNPKCARCRNH--GVVSTLKGHKKMCHWK-NCTCTRCQLVLERQRVMAVQVALRRYQQA----------QSDLQ--------------------------------------------------------SS-H

>Eaff_DmrtA2-like

YQRTPKCARCRNH--GLVSALKGHKRYCRWR-DCMCAKCTLIAERQRVMAAQVALRRQQAQ----------EE-AE--------------------------------------------------------AR-Q

>Btab_Dmrt2-like

RLRTPKCARCRNH--GIISYLRGHKKLCRWR-ECRCANCLLIIQRQKVMAAQVALRRHQIN----------LK-ND--------------------------------------------------------GY-R

>Xlae_Dmrt4

YPRTPKCARCRNH--GVVSALKGHKRFCRWR-DCSCAKCTLIAERQRVMAAQVALRRQQAQ----------EE-CE--------------------------------------------------------VR-D

>Bmut_Dmrt1

SPRLPKCARCRNH--GYASPLKGHKRFCMWR-DCQCKKCNLIAERQRVMAAQVALRRQQAQ----------EE-EL--------------------------------------------------------GI-S

>Mmus_Dmrt1

SPRLPKCARCRNH--GYASPLKGHKRFCMWR-DCQCKKCSLIAERQRVMAAQVALRRQQAQ----------EE-EL--------------------------------------------------------GI-S

>Xlae_Dmrt1

SPRLPKCARCRNH--GYASPLKGHKRYCMWR-DCQCKKCSLIAERQRVMAAQVALRRQQAQ----------EE-EL--------------------------------------------------------GI-S

>Drer_Dmrt1

PSRMPKCSRCRNH--GFVSPLKGHKRFCNWR-DCQCQKCRLIAERQRVMAAQVALRRQQAQ----------EE-EM--------------------------------------------------------GI-C

>Tcal_DM

AQRTPKCARCRNH--GIVSALKGHKRYCRWK-DCFCAKCTLIAERQRVMAAQVALRRQQAQ----------EE-AE--------------------------------------------------------AK-Q

>Bmor_Dmrt93B

RARVPKCARCRNH--GLISSLRGHKKACAYR-HCQCPKCGLIKERQRIMAAQVALKRQQAA----------ED-KI--------------------------------------------------------AL-H

>Tcas_Dmrt93B

SARVPKCARCRNH--GMISTLRGHKKQCIYK-NCSCAKCGLIKERQRIMAAQVALKRQQAA----------ED-AI--------------------------------------------------------AL-H

>Btab_DmrtA2-like

GGRRPKCARCRNH--GLISWLKGHKRQCRFK-SCVCPKCNLIAERQRVMAAQVALKRQQAA----------ED-AI--------------------------------------------------------AL-G

>Caqu_DM2

GARLPKCARCRNH--GMISWLKGHKRHCRFK-DCICAKCNLIAERQRVMAAQVALKRQQAA----------ED-AI--------------------------------------------------------AL-G

>Edan_Dmrt93B

GARRPKCARCRNH--GMISWLKGHKRHCKYK-DCACVKCNLIAERQRVMAAQVALKRQQAA----------ED-AI--------------------------------------------------------AL-G

>Tdom_Dmrt93B

GARRPKCARCRNH--GMISWLKGHKRHCRFK-DCVCAKCNLIAERQRVMAAQVALKRQQAA----------ED-AI--------------------------------------------------------AL-G

>Hazt_DM2

GGRKPKCARCRNH--GMISWLKGHKRHCKFK-DCTCVRCNLIAERQRVMAAQVALKRQQAA----------ED-AL--------------------------------------------------------AL-T

>Emex_DmertA2-like

KQRRPKCARCRNH--GLISWLRGHKRECRYR-ECLCPKCSLIAERQRVMAAQVALKRQQAA----------ED-AI--------------------------------------------------------AL-K

>Fvar_DmrtA2-like

KQRRPKCARCRNH--GLISWLRGHKRECRYR-ECLCPKCSLIAERQRVMAAQVALKRQQAA----------ED-AI--------------------------------------------------------AL-K

>Nlug_Dsx-like2

KGRHPKCARCRNH--GMISWLKGHKKQCMFK-SCSCAKCSLIAERQRVMAAQVALKRQQAA----------ED-TI--------------------------------------------------------AL-G

>Dmag_Dmrt93B

ALRRPKCARCRNH--GVISWLKGHKRHCRFK-DCLCVKCNLIAERQRVMAAQVALKRQQAT----------ED-AI--------------------------------------------------------AL-G

>Hdeu_Dmrt

KVRRPKCARCRNH--GVISWLKGHKKQCRFR-LCACAKCNLIAERQRVMAAQVALKRQQAA----------ED-AV--------------------------------------------------------AL-G

>Dmel_Dmrt93B

TNRVPKCARCRNH--GIISELRGHKKLCTYK-NCKCAKCVLIFERQRIMAAQVALKRQQAV----------ED-AI--------------------------------------------------------AM-R

>Lcup_Dmrt93B

TNRVPKCARCRNH--GWISELRGHKKHCTYK-NCRCAKCVLIFERQRIMAAQVALKRQQAV----------ED-AI--------------------------------------------------------AL-R

>Phum_Dsx

PRTPPNCARCRNH--DIKIALKNHKRYCRYK-HCKCTKCILTPQRQTVMAAQTALRRAQAQ----------DE-AR--------------------------------------------------------GI-K

>Nlug_Dsx-like1a

QTRIPNCARCRNH--FVTTPLKGHKNYCKFR-KCTCEGCLLIKKRQVVMAEHTALRREKAL----------FK-DR--------------------------------------------------------LV--

>Rpro_Dsx

ARTPPNCARCRNH--SKTEPLKGHKRFCKYR-TCTCKKCHLTVERQREMAKQTALRRELAQ----------DE-AR--------------------------------------------------------AR-A

>Hvit_DM

SRTPPNCARCRNH--EVILPLKGHKRYCKFM-DCKCEKCELTAERQKVMARQTAIRRAEEQ----------DR-QR--------------------------------------------------------LK-S

>Dcit_DmrtA2-like

GRTTPHCARCRNH--KISVPLQAHKRYCPFR-DCTCKDCILTKERQRVMALQTALRRQQQQ----------DE-MR--------------------------------------------------------AK-K

>Cdip_DM

TRTLPKCARCKNH--SVIAPLRGHKRYCKHR-LCECDKCVLVAERQRVMARQTALRRAQAQ----------DE-LL--------------------------------------------------------IR-Q

>Cdip_DM2

TRTPPRCARCRNH--SIKVPLRGHKRYCVYR-SCKCPKCKLTAERQKVMAQQTALRRAQTQ----------DE-LF--------------------------------------------------------GR-Q

>Zger_DM3

PRQKPNCAKCKHHIKNGTIPVRYHRRYCKYE-NCVCKKCISVDERRDRMAKQVAIRRKISQ----------DV-RL--------------------------------------------------------GYIE

>Aamp_DmrtC2-like

ITRSPLCSRCRNH--GIKISLRGHKRFCGYK-ECLCDKCALVKARQRVMARQVALRRAQEH----------EE-VR--------------------------------------------------------GQ-A

>Nvit_Dsx

SQRIPKCTRCQNH--GKKVQVKFHKRECEFR-YCLCEMCILTTKRQQIMKVQTAQRRARQQ----------HE-ML--------------------------------------------------------ME-M

>Tsar_Dsx

SQRIPQCTRCQNH--GKKVRVKFHKRECEFR-YCLCRECILTTKRQQIMKVQTAQRRAREQ----------RE-LL--------------------------------------------------------KE-M

>Caqu_Dsx

NSRTPKCARCRNH--KLNIAVKGHKRYCRYR-DCMCEKCRLTAERQRVMALQVALRRAQVR----------QA-AF--------------------------------------------------------NS--

>Afra_Dsx3

RTRVPTCARCRNH--GKITKLRGHKRYCQFR-ACSCKLCVLTVDKQRVMAAQVANRRALKQ----------DE-EN--------------------------------------------------------GV--

>Afra_Dsx4

KMRMPTCARCRNH--GQVVKLRGHKRYCSFR-HCLCDRCALTSEKQRVMAAQVALRRAQKQ----------DE-EN--------------------------------------------------------GI-V

>Hdeu_Dsx

SARTPKCARCRNH--KVNVPVKGHKRYCEYR-YCECERCVLTAERQRVMALQDEARAAQLR----------HQ-AA--------------------------------------------------------TS-P

>Zcau_DM

VSRTPKCGRCRNH--GAINELKGHKDACRWK-DCSCSRCALLIARQKINQDLKILKEKEKQ----------KE-ET--------------------------------------------------------GT-Q

>Zger_DM

KPRIPMCGRCRNH--GVINALKGHKDACRWK-NCKCSRCSLLIARQKINQDLKVLKEQKRK----------DE-EA--------------------------------------------------------AT-R

>Zcau_DM3

ERAKPTCEKCKHH--GIIIFLEGHKNRCKYKDELDCEMCLAVDEKRRVMKDQTQLTREITM----------NI-ER--------------------------------------------------------MN--

>Zger_DM2

PRAKPTCEKCKHH--NIIVLLDGHKKRCRYRNNLDCEMCIKVDEKRKVMKDQTKLSREISM----------KT-AR--------------------------------------------------------AE--

>Auri_DM2

TRTGPACARCRHH--GITNYLKGHKRYCRFR-NCCCSKCNLTRLRQKVMANQSALRRAEYL----------EK-SR--------------------------------------------------------VAFE

>Nlug_Dsx

NRTSPKCVRCRNH--NEVSLLSGHKRFCRFR-HCVCRNCQRTVKRQKKMAKQIAIRRRKKL----------EE-ER--------------------------------------------------------GE--

>Dcar_Dsx1

SQRHPTCALCKNH--QTISTLKGHKRYCPWR-QCMCELCYGTNKKRKINAEQVALRRAQAQ----------DE-EL--------------------------------------------------------RK-K

>Dmag_Dsx1

SQRHPTCALCKNH--QTISTLKGHKRYCPWR-QCMCELCYGTNKKRKINAEQVALRRAQAQ----------DE-EL--------------------------------------------------------RK-K

>Dgal_Dsx1

TQRHPTCALCKNH--QTISTLKGHKRYCPWR-QCMCELCYGTNKKRKINAEQVALRRAQAQ----------DE-EL--------------------------------------------------------RK-K

>Dpul_Dsx1

TQRHPTCALCKNH--QTISTLKGHKRYCPWR-QCMCELCYGTNKKRKINAEQVALRRAQAQ----------DE-EL--------------------------------------------------------RK-K

>Mmac_Dsx

NQRHPTCALCKNH--QTISTLKGHKRYCPWR-TCLCELCYSTNKKRKINAEQVALRRAQAQ----------DE-EL--------------------------------------------------------RK-K

>Dcar_Dsx2

SCRNPTCALCKNH--GINSPLKGHKRYCPFG-RCSCDLCRVTRKKQKINASQVASRRAQQQ----------DR-EL--------------------------------------------------------GI-D

>Dmag_Dsx2

SCRNPTCALCKNH--GINSPLKGHKRYCPFG-RCSCDLCRVTRKKQKINASQVASRRAQQQ----------DR-EL--------------------------------------------------------GI-D

>Dgal_Dsx2

SCRNPTCALCKNH--GINSPLKGHKRYCPFG-RCSCDLCRVTRKKQKINASQVATRRAQQQ----------DR-EL--------------------------------------------------------GI-D

>Dpul_Dsx2

SCRNPTCALCKNH--GINSPLKGHKRYCPFG-RCSCDLCRVTRKKQKINASQVATRRAQQQ----------DR-EL--------------------------------------------------------GI-D

>Ocin_Dmrt1

KPRNPNCALCQNH--GKVVPIKAHKRYCPWK-DCTCSKCNLTNYRRKFVASQIAARRAVIQ----------DR-ER--------------------------------------------------------KMME

>Acer_DM

KKTTPLCARCKNH--GLKIKLKGHKRYCRYI-RCICQECIATKARQIKMARQVYFRRNCQNAV--------DQ-ND--------------------------------------------------------NS-G

>Btab_Dsx

SRAPRCCVRCRNH--GLKKLVRGHKRYCPYT-LCPCRLCLATKERQVHMAKTIKKRRYFLQ----------DL-AM--------------------------------------------------------QA-H

>Hazt_DM3

LTSRQLCDKCRNH--IKFSKKRGHKGKCQFE-NCKCAYCHLTDMRRLIMKHQQRVRRANVTAKLISSEIDDDEDSKS------------------------------------------------------ESH-E

>Hazt_DM4

LTTRRLCDKCRNH--SCFKTKRGHKKFCAYE-ACKCVLCQLTDKRRLIMKYQQRVRRVNVTAKAIPPNNELRESENE------------------------------------------------------QSA-E

>Sver_iDMY

SSKQQKCDMCRNH--GVMKEKRAHKNACPYQ-DCLCALCGLTKKRRDVMRHQQRVRRSCGE----PV----PC-RR--------------------------------------------------------SP-P

>Sver_iDmrt1

SSKQQKCDMCRNH--GFMKEKRAHKNACPYQ-DCSCALCGLTRKRRDIMRHQQRVRRSQVT----------SQ-QR--------------------------------------------------------DE-A

>Cqua_Dsx

GKRKQRCRMCANH--GIYVEVKGHKWYCPYRENHNCEKCEITRKRQYYMAEQQKLTREQQQ----------QR-EF--------------------------------------------------------QR-G

>Sver_Dsx

NKRKQRCRMCANH--GVYVEVKGHKWVCPYRLKHNCEKCEITKKRQYYMAEQQKLTRDQQQ----------QQ-LN--------------------------------------------------------PH-G

**Supplementary Sequence 2**

>Aage_Dsx-like

PRAPPNCARCRNHRLKIELKGHKRYCKYRYCNCEKCRLTADRQRVMALQTALRRAQAQDEARARA

>Harm_Dsx

PRAPPNCARCRNHRLKIELKGHKRYCKYRNCTCEKCRLTADRQRVMALQTALRRAQAQDEARARA

>Bmor_Dsx

PRAPPNCARCRNHRLKIELKGHKRYCKYQHCTCEKCRLTADRQRVMAKQTAIRRAQAQDEARARA

>Ekue_Dsx

PRAPPNCARCRNHRLKIELKGHKRYCKYRNCMCEKCRLTADRQRVMALQTALRRAQAQDEARARS

>Msex_Dsx

PRAPPNCARCRNHRLKIELKGHKRYCKYRYCTCEKCRLTADRQRVMAMQTAMRRAQAQDEARARA

>Ofur_Dsx

PRAPPNCARCRNHRLKVELKGHKRECKYRYCTCEKCRLTADRQRVMALQTALRRAQAQDEARARS

>Osca_Dsx

PRAPPNCARCRNHRLKVELKGHKRYCKYRYCTCEKCRLTADRQRVMALQTALRRAQAQDEARARS

>Paeg_Dsx

PRAPPNCARCRNHRLKIELKGHKRYCKYRYCTCEKCRLTADRQRVMALQTALRRAQAQDEARARA

>Pmac_Dsx

PRAPPNCARCRNHRLKVELKGHKRYCKYRYCTCEKCRLTADRQRVMAMQTALRRAQAQDEARARA

>Pxyl_Dsx

PRAPPNCARCRNHRLKVELKGHKRYCKYRYCNCEKCRLTADRQRVMALQTALRRAQAQDEARARA

>Obru_Dsx

PRAPPNCARCRNHRLKVELKGHKRYCKYRYCTCEKCRLTADRQRVMALQTALRRAQAQDEARARH

>Rnub_DM

PRTPPNCARCRNHRLKIALKGHKRYCKYRFCNCDKCRLTAERQRVMALQTALRRAQAQDEARARV

>Amel_Dsx

PRA-RNCARCLNHRLEITLKSHKRYCKYRTCTCEKCKITANRQQVMRQNMKLKRHLAQDKVKVRV

>Bpyr_Dsx

PRAPRNCARCRNHRLKITLKSHKRYCKYRYCNCEKCKITADRQRVMAQQTKLRRQLAQDEVKVRA

>Emex_Dsx

PRAPRNCARCRNHRLKITLKSHKRYCKYRYCNCEKCKITADRQRVMAQQTKLRRQLAQDEVKVRA

>Fvar_Dmrt1

PRAPRNCQRCRNHRLKITVKSHKRYCKYRFCECDRCKVTADKQRVTAMQAKLRRQLAQDEVKVRV

>Cgig_Dsx

PRTPPNCARCRNHRLKIPLKGHKRYCKYRYCKCEKCKLTADRQRVMAKQTALRRALAQDEVKIRA

>Olig_Dsx

PRKPPNCARCRNHQLKIAVKGHKRYCRYRYCKCDSCKLTADRQRVMARQTALRRALALDEVRVRA

>Aros_Dsx

PRTPPNCARCRNHRLKIALKGHKRYCKYRSCNCEKCRLTAERQRVMALQTALRRAQAQDEARVRG

>Xalp_Dsx

PRTPPNCARCRNHRLKIALKGHKRYCKYRYCNCEKCRLTAERQRVMALQTALRRAQAQDEARVRG

>Nlec_Dsx

PRTPPNCARCRNHRLKIALKGHKRYCKYRSCNCEKCRLTAERQRVMALQTALRRAQAQDEARVRG

>Bdor_Dsx

PRTPPNCARCRNHGLKITLKGHKRYCKFRFCTCEKCRLTADRQRVMALQTALRRAQAQDEQRVLQ

>Btry_Dsx

PRTPPNCARCRNHGLKITLKGHKRYCKFRFCTCEKCRLTADRQRVMALQTALRRAQAQDEQRVLQ

>Ccap_Dsx

PRTPPNCARCRNHGLKITLKGHKRYCKFRYCTCEKCRLTADRQRVMALQTALRRAQAQDEQRVLQ

>Hpal_DM

PRTPPNCARCRNHNFKIGLKGHKRYCRFRSCTCSKCCLTAERQRVMALQTALRRAQAQDEKYYEQ

>Icra_Dsx

PRTPPNCARCRNHRVKIPLKGHKRYCKYRTCNCQKCRLTAERQRVMAMQTALRRAQAQDEAMLHA

>Xant_Dsx

PRTPPNCARCRNHRVKIPLKGHKRYCKYRTCNCQKCRLTAERQRVMAMQTALRRAQAQDEAMLHA

>Lmig_Dsx

QRTPPNCARCRNHGYKIPLKGHKRYCKYRYHTCDKCLLTAERQRVMAMQTALRRAQAQDEAMGLK

>Tsub_Dsx

QRTPPNCARCRNHGLKIPLKGHKRYCKYRYHDCDKCLLTAERQRVMALQTALRRAQAQDEALAAS

>Kbie_DM

PRTPPNCARCRNHRLKIGLKGHKRYCKFRSCKCEKCCLTAERQRVMAAQTALRRAQAQDEQHLTS

>Tgla_DM

PRTPPNCARCRNHRLKIGLKGHKRYCKFRYCTCDKCRLTAERQRVMAAQTALRRAQAQDEKYLSS

>Mext_Dsx

PRTPPNCARCRNHRLKIGLKGHKRYCKYRYCTCSKCRLTAERQRVMALQTALRRAQAQDEKYLAQ

>Mfas_Dsx

PRTPPNCARCRNHGLKITLKGHKRYCKYRYCTCEKCRLTADRQRVMALQTALRRAQAQDEQRALQ

>Dmel_Dsx

PRTPPNCARCRNHGLKITLKGHKRYCKFRYCTCEKCRLTADRQRVMALQTALRRAQAQDEQRALH

>Epen_Dsx

PRTPPNCARCRNHRLKIGLKGHKRYCKYRYCNCDKCCLTAERQRVMALQTALRRAQAQDEARQQH

>Mrel_Dsx

PRTPPNCARCRNHRLKIGLKGHKRYCKYRYCNCDKCCLTAERQRVMALQTALRRAQAQDEARQQH

>Gnip_DM

PRTPPNCARCRNHRLKIGLKGHKRYCKYRHCTCDKCCLTAERQRVMAMQTALRRAQAQDETLATQ

>Gryl_DM

PRTPPNCARCRNHRLKIGLKGHKRYCKYRACTCDKCCLTAERQRVMAMQTALRRAQAQDETLATQ

>Cpun_Dsx

PRTPPNCARCRNHRLKIGLKGHKRYCKYRYCNCDKCCLTAERQRVMALQTALRRAQAQDEARMAT

>Bger_Dsx

PRTPPNCARCRNHRLKIGLKGHKRYCTFRSCVCEKCVLTAERQRVMALQTALRRAQAQDEARERQ

>Tdom_Dsx

ART-PKCARCRNHRLKIPLKGHKRYCKYRFCNCDKCLLTAERQRVMALQTALRRAQAQDEARAAG

>Gcor_Dsx

PRTPPNCARCRNHRMKIALKGHKRYCKYRTCKCEKCRLTSERQRVMAMQTALRRAQAQDEAMMRN

>Tcas_Dsx

PRTPPNCARCRNHRLKIALKGHKRYCKYRTCKCEKCRLTTERQRVMAMQTALRRAQAQDEAMLRS

>Otau_Dsx

PRTPPNCARCRNHRVKVPLKGHKRYCKYRHCKCEKCRLTSERQRVMAMQTALRRAQAQDEAMLRQ

>Tdic_Dsx

PRTPPNCARCRNHRLKIALKGHKRYCKYRHCKCEKCRLTSERQRVMAMQTALRRAQAQDEAMLRQ

>Mviol_Dsx

PRTPPNCARCRNHRMKVALKGHKRYCKFRTCKCEKCRLTSERQRVMAMQTALRRAQAQDEAMMKT

>Hydr_Dsx

PRTPPNCARCRNHRLKIALKGHKRYCKFRHCNCDRCRLTAERQRVMALQTALRRAQAQDEARAKA

>Ctos_DM

PRTPPNCARCRNHLLKIALKGHKRFCRYRYCDCDKCRLTAERQRVMAAQTALRRNQAQDEAIMAA

>Same_DM

PRTPPNCARCRNHLLKIALKGHKRFCRYRYCDCDKCCLTAERQRVMAAQTALRRNQAQDEAIMAN

>Ptap_DM

PRTPPNCARCRNHLLKIALKGHKRFCRYRDCNCDKCLLTLERQRVMAAQTALRRNQAQDEALMAT

>Annu_Dsx

PRTPPNCARCRNHRLKIALKGHKRYCKYRYCNCDKCRLTAERQRVMALQTALRRAQAQDEARARS

>Ppra_Dsx

PRTPPNCARCRNHRLKIPLRGHKRYCRFRNCICHKCKLTAERQRVMAMQTALRRAQAQDEAMQTS

>Bhye_Dsx

PRTPPNCARCRNHRLKIALKGHKRYCKFRNCNCYKCILTAERQRIMAVQTAQRRAQAQDEAREAK

>Ccor_Dsx

PRTRPNCARCRNHRVKVPLKGHKRYCKYRTCSCQKCCLTAERQRVMAMQTALRRAQAQDEAMLNS

>Lcup_Dsx

PRTKPNCARCHNHGFKIKLKGHKRYCKYRNCNCEKCRLTADRQRVMALQTALRRAQQQDEQRILQ

>Mdom_Dsx

PRTKPNCARCHNHGLKIKLKGHKRYCKYRFCNCEKCRLTADRQRVMALQTALRRAQQQDEARILQ

>Mmol_DM

PRTPPNCARCRNHQLKIPLKGHKRYCRYRSCECGKCRLTSERQRVMAMQTALRRAQAQDEIYYQA

>Acha_DM

KRKTPNCARCRNHYVKIKLKGHKRYCKYRNCRCNKCTLTAERQRIMAAQTAIRRSQAQDEERIEQ

>Dipl_DM

HRKAPNCARCRNHYLKIELKGHKRYCKFRFCRCDKCTLTAERQRVMALQTALRRGQAQDEQRMAR

>Faur_DM

HRKAPNCARCRNHYLKMGLKGHKRYCKFRYCECSKCRLTYERQRVMALQTALRRGQAQDEQRLAR

>Faur_DM2

HRKAPNCAKCRNHSLNNGLKGHKRYCKFRFCQCAKCRLTVQRQRVMAKQTAVRRAQAQDEQREGQ

>Gmar_DM

QRKAPKCARCRNHYLKIGLKGHKRYCKYRFCQCKKCELTAERQRVMAQQTALRRGQAQDEIRLAR

>Gmar_DM2

QRTAPNCARCRNHYLTIELKGHKRYCKFRYCHCFKCQLTAKRQRVMAQQTALRRSQAQDEKRLYG

>Smel_DM

PRTPPNCARCRNHLLKIPLKGHKRYCRYRTCACNKCCLTSERQRVMAMQTALRRAQAQDEMFFQT

>Xves_DM

PRTPPNCARCRNHLLKIPLKGHKRYCRYRTCACNKCCLTSERQRVMAMQTALRRAQAQDEMFFQN

>Pcat_DM

SRTAPNCARCRNHGLKIELKGHRRYCKYRSCGCGKCSLTAARQRIMASQTALRRAQAQDERYQQI

>Pcat_DM2

SLTDSKCARCRNHNMKVELKGHKRYCKYRSCGCVKCSLTAERQTIIALQLAVRRTQAQDERYRQI

>Enos_Dsx

PRTPPNCARCRNHRLKIPLRGHKRYCRFRTCTCEKCRLTAERQRVMAMQTALRRAQAQDEAMLSA

>Aasp_Dsx-like

NSMNRLCALCRNHGLKIPVKGHKRFCGYRLCLCKECCLVKERQRVVAMHLYFRRAQEQEEADRGS

>Mext_Dsxlike

NGMNRLCALCRNHGLKIPVKGHKRFCGYRLCLCKECCLVKERQRVVAMHLYFRRAQEQEEADRGT

>Choo_Dsxlike

NSINRLCALCRNHGLKIPVKGHKRYCGYRLCLCKECCLVKERQRVVAMHLYFRRAQEQEEADRGD

>Psch_Dsxlike

NNMNRLCALCRNHGLKMPVKGHKRFCGYRLCLCKECCLVKERQRVVAMHLYFRRAQEQEESDKGA

>Tcri_Dsx-like

NNLNRLCALCRNHGLKKPVKGHKRFCAYKLCVCRECCLVKERQRVVALHLYYRRAQDQEENDRKP

>Tdom_Dsx-like

--KTPSCAQCRNHGLRVPVKGHKRYCAYKLCTCKECCLVKERQRVVALHLYVRRAQDQEEADSSQ

>Tger_Dsx-like

KIPSRLCAQCRNHGLRVPVKGHKRFCAYKLCHCAECLLVKERQRVVALHLYIRRAQDQEEADSSQ

>Edan_Dsxlike

TSSNRLCAQCRNHGLRVPVRGHKRYCTFRLCTCRECRLVRERQRVVALHLYVRRAQEQEEEAAEA

>Eins_Dsxlike

QGSNRLCAQCRNHGLRVPVRGHKRYCTFRLCSCRDCRLVRERQRVVALHLYVRRAQEQEEEAAAA

>Eury_Dsx2

KTASRLCAFCRNHSLKIPVKGHKRFCRNRTCNCAECKLVRERQRVVALHLYVRRAQEQEEEAAAA

>Ibic_Dsxlike

QGSNRLCAQCRNHGLRVPVRGHKRYCTYRLCTCRECRLVRERQRIVALHLYVRRAQEQEEEAAAA

>Baet_Dsx

ALSNRMCAQCQNHGLKIPVRGHKRFCKYRLCNCQNCLLVKERQRIVALHLYVRRAQQQEEDAAEA

>Cdip_DM3

PVSKRMCAQCQNHGLKEPVRGHKRHCKYRLCNCKNCLLVKERQRIVALHLYVRRAQQQEEDAAEA

>Afus_B

SPRTPKCARCRNHKKSIPVKGHKRYCEFRSCTCEKCCLTAERQRVMALQVAVRRAQAQDEARAAQ

>Esup_Dsx

ARTPPKCARCRNHRLKIPLKGHKRYCKFRYCKCDKCRLTAERQRVMAMQTALRRAQAQDEANRGM

>Isen_Dsx

ARTPPKCARCRNHLLKIALKGHKRYCKYRYCKCDKCRLTAERQRVMAMQTALRRAQAQDEANRGM

>Lful_Dsx

ARTPPKCARCRNHRLKIPLKGHKRYCKFRYCKCDKCRLTAERQRVMAMQTALRRAQAQDEANRGL

>Cfel_Dsx

PRTPPNCARCRNHRLKIPLKGHKRYCRYLYCKCEKCRLTADRQRDMARQTAMRRAQAQDEARGLS

>Cgal_Dsx

PRTPPNCARCRNHRLKIALKGHKRYCRFLYCKCEKCKLTADRQRVMAKQTALRRAQAQDEARGLS

>Atub_Dsx

PRTKPNCARCGNHHLKMRLRGHKRYCKYRYCTCEKCILTTDRQKVMALQTALRRAQEQDATREEP

>Tpal_DmrtA2

ARTPPNCALCRNHRLKIGLKGHKRYCKYRYCDCDKCRLTAERRRVMALQTALRRAQAQDEARHPV

>Edan_Dsx

ARTPPKCARCRNHRLKIPLKGHKRYCKYRFCNCYKCTLTADRQRVMAEQTALRRAQAQDEAFAAC

>Focc_Dsx

ARTPPNCALCRNHRLKIGLKGHKRYCKYRYCDCDKCQLTAERRRVMALQTALRRAQAQDEQRQPN

>Afus_A

ILRTPKCARCRNHGVISCLRGHKKMCRWKECICEMCELVAERQRIMAAQVALRRQQIGQKLKETN

>Afus_C

YQRTPKCARCRNHGVVSALKGHKRYCRWRDCVCAKCTLIAERQRVMAAQVALRRQQAQEENEARE

>Caqu_DM1

YQRTPKCARCRNHGVVSALKGHKRYCRWRDCVCAKCTLIAERQRVMAAQVALRRQQAQEENEARE

>Tdom_Dmrt99B

YQRTPKCARCRNHGVVSALKGHKRYCRWRDCVCAKCTLIAERQRVMAAQVALRRQQAQEENEARE

>Annu_Dmrt99B

YQRTPKCARCRNHGVVSALKGHKRYCRWRDCVCAKCTLIAERQRVMAAQVALRRQQAQEESEARE

>Mext_Dmrt99B

YQRTPKCARCRNHGVVSALKGHKRYCRWRDCACAKCTLIAERQRVMAAQVALRRQQAQEESEARE

>Lstri_DM

YQRTPKCARCRNHGVVSALKGHKRYCRWRDCMCAKCTLIAERQRVMAAQVALRRQQAQEESEARE

>Clec_DmrtA2-like

YQRTPKCARCRNHGVVSALKGHKRYCRWRDCVCAKCTLIAERQRVMAAQVALRRQQAQEESEARE

>Gbue_DM1

YQRTPKCARCRNHGVVSALKGHKRYCRWRDCVCAKCTLIAERQRVMAAQVALRRQQAQEENEARE

>Dmel_Dmrt99B

YQRTPKCARCRNHGVVSALKGHKRYCRWRDCVCAKCTLIAERQRVMAAQVALRRQQAQEENEARE

>Lcup_Dmrt99B

YQRTPKCARCRNHGVVSALKGHKRYCRWRDCLCAKCTLIAERQRVMAAQVALRRQQAQEENEARE

>Mdom_Dmrt99B

YQRTPKCARCRNHGVVSALKGHKRYCRWRDCLCAKCTLIAERQRVMAAQVALRRQQAQEENEARE

>Edan_Dmrt99B

YQRTPKCARCRNHGVVSALKGHKRYCRWRDCVCAKCTLIAERQRVMAAQVALRRQQAQEENEARE

>Tpal_DmrtA2-like

YQRTPKCARCRNHGVVSALKGHKRYCRWRDCVCAKCTLIAERQRVMAAQVALRRQQAQEESEARE

>Tcas_Dmrt99B

YQRTPKCARCRNHGVVSALKGHKRYCRWRDCNCAKCTLIAERQRVMAAQVALRRQQAQEENEARE

>Bmor_Dmrt99B

YQRTPKCARCRNHGVVSALKGHKRYCRWRDCVCAKCTLIAERQRVMAAQVALRRQQAQEENEARE

>Mmol_DM2

YQRTPKCARCRNHGVVSALKGHKRYCRWRDCVCAKCTLIAERQRVMAAQVALRRQQAQEENEARE

>Esin_Dmrt-like

YQRTPKCARCRNHGVVSALKGHKRYCRWRDCNCAKCTLIAERQRVMAAQVALRRQQAQEENEVRE

>Spar_DM

YQRTPKCARCRNHGVVSALKGHKRYCRWRDCNCAKCTLIAERQRVMAAQVALRRQQAQEENEVRE

>Emex_DmertA2

YQRTPKCARCRNHGVVSALKGHKRYCRWRDCVCAKCTLIAERQRVMAAQVALRRQQAQEESEARE

>Fvar_DmrtA2

YQRTPKCARCRNHGVVSALKGHKRYCRWRDCVCAKCTLIAERQRVMAAQVALRRQQAQEESEARE

>Dmag_Dmrt99B

YQRTPKCARCRNHGVVSALKGHKRYCRWRDCACAKCTLIAERQRVMAAQVALRRQQAQEENEARE

>Xlae_Dmrt5

YPRTPKCARCRNHGVVSALKGHKRYCRWKDCMCAKCTLIAERQRVMAAQVALRRQQAQEENEARE

>Eaff_DmrtA2-like

YQRTPKCARCRNHGLVSALKGHKRYCRWRDCMCAKCTLIAERQRVMAAQVALRRQQAQEEAEARQ

>Bmor_Dmrt11E

ALRTPKCARCRNHGVISCLKGHKRLCRWRDCRCPGCLLVLERQRVMAAQVALRRQQGAGGPESRN

>Caqu_DM3

LLRTPKCARCRNHGVISCLKGHKKLCRWRECQCPNCLLVVERQRVMAAQVALRRQQSSEDNSKDG

>Ceut_Dmrt11E

LLRTPKCARCRNHGVISCLKGHKRLCRWRECQCPNCQLVVERQRVMAAQVALRRQQSSEEGQDVR

>Kbie_DM2

LLRTPKCARCRNHGVISCLKGHKRLCRWRECQCPNCQLVVERQRVMAAQVALRRQQSSEDGQDSR

>Rvir_DM

LLRTPKCARCRNHGVISCLKGHKRLCRWRECQCPNCQLVVERQRVMAAQVALRRQQSSEDSDDR-

>Gdja_DM

LLRTPKCARCRNHGVISCLKGHKRLCRWRECQCPNCQLVVERQRVMAAQVALRRQQSSEEGQDVR

>Tdom_Dmrt11E

LLRTPKCARCRNHGVISCLKGHKKLCRWRECQCPNCLLVVERQRVMAAQVALRRQQSSEDSKDSR

>Choo_Dmrt11E

LLRTPKCARCRNHGVISCLKGHKRLCRWRECRCPNCQLVVERQRVMAAQVALRRQQSSEDGPESH

>Dmag_Dmrt11E

LLRTPKCARCRNHGVVSCLKGHKKLCRWKECQCTNCLLVVERQRVMAAQVALRRQQNSESAKSDG

>Edan_Dmrt11E

VLRTPKCARCRNHGVISGLKGHKRLCRWRECRCPSCLLVVERQRVMAAQVALRRQQSSEEGGNSG

>Lcup_Dmrt11E

MLRTPKCARCRNHGVISCVKGHKKLCRWRECTCPNCQLVVDRQRVMAAQVALRRQQTMEEPYETN

>Mdom_Dmrt11E

MLRTPKCARCRNHGVISCVKGHKKLCRWRECTCQNCQLVVDRQRVMAAQVALRRQQTTLADENNS

>Dmel_Dmrt11E

LLRTPKCARCRNHGVISCVKGHKRLCRWRECCCPNCQLVVDRQRVMAAQVALRRQQTMEALEATA

>Eury_Dmrt11E

VLRSPKCARCRNHGVISSLKGHKRLCRWRECRCPSCLLVVERQRVMAAQVALRRQHCNSEKKCSS

>Drer_Dmrt2

LSRTPKCARCRNHGVVSCLKGHKRFCRWRDCQCANCLLVVERQRVMAAQVALRRQQATEDKKGIT

>Btau_Dmrt2

LSRTPKCARCRNHGVVSCLKGHKRFCRWRDCQCANCLLVVERQRVMAAQVALRRQQATEDKK---

>Xlae_Dmrt4

YPRTPKCARCRNHGVVSALKGHKRFCRWRDCSCAKCTLIAERQRVMAAQVALRRQQAQEECEVRD

>Bmut_Dmrt1

SPRLPKCARCRNHGYASPLKGHKRFCMWRDCQCKKCNLIAERQRVMAAQVALRRQQAQEEELGIS

>Mmus_Dmrt1

SPRLPKCARCRNHGYASPLKGHKRFCMWRDCQCKKCSLIAERQRVMAAQVALRRQQAQEEELGIS

>Xlae_Dmrt1

SPRLPKCARCRNHGYASPLKGHKRYCMWRDCQCKKCSLIAERQRVMAAQVALRRQQAQEEELGIS

>Drer_Dmrt1

PSRMPKCSRCRNHGFVSPLKGHKRFCNWRDCQCQKCRLIAERQRVMAAQVALRRQQAQEEEMGIC

>Tcal_DM

AQRTPKCARCRNHGIVSALKGHKRYCRWKDCFCAKCTLIAERQRVMAAQVALRRQQAQEEAEAKQ

>Bmor_Dmrt93B

RARVPKCARCRNHGLISSLRGHKKACAYRHCQCPKCGLIKERQRIMAAQVALKRQQAAEDKIALH

>Tcas_Dmrt93B

SARVPKCARCRNHGMISTLRGHKKQCIYKNCSCAKCGLIKERQRIMAAQVALKRQQAAEDAIALH

>Caqu_DM2

GARLPKCARCRNHGMISWLKGHKRHCRFKDCICAKCNLIAERQRVMAAQVALKRQQAAEDAIALG

>Edan_Dmrt93B

GARRPKCARCRNHGMISWLKGHKRHCKYKDCACVKCNLIAERQRVMAAQVALKRQQAAEDAIALG

>Tdom_Dmrt93B

GARRPKCARCRNHGMISWLKGHKRHCRFKDCVCAKCNLIAERQRVMAAQVALKRQQAAEDAIALG

>Emex_DmertA2-like

KQRRPKCARCRNHGLISWLRGHKRECRYRECLCPKCSLIAERQRVMAAQVALKRQQAAEDAIALK

>Fvar_DmrtA2-like

KQRRPKCARCRNHGLISWLRGHKRECRYRECLCPKCSLIAERQRVMAAQVALKRQQAAEDAIALK

>Dmag_Dmrt93B

ALRRPKCARCRNHGVISWLKGHKRHCRFKDCLCVKCNLIAERQRVMAAQVALKRQQATEDAIALG

>Dmel_Dmrt93B

TNRVPKCARCRNHGIISELRGHKKLCTYKNCKCAKCVLIFERQRIMAAQVALKRQQAVEDAIAMR

>Lcup_Dmrt93B

TNRVPKCARCRNHGWISELRGHKKHCTYKNCRCAKCVLIFERQRIMAAQVALKRQQAVEDAIALR

>Hdeu_Dmrt

KVRRPKCARCRNHGVISWLKGHKKQCRFRLCACAKCNLIAERQRVMAAQVALKRQQAAEDAVALG

>Phum_Dsx

PRTPPNCARCRNHDIKIALKNHKRYCRYKHCKCTKCILTPQRQTVMAAQTALRRAQAQDEARGIK

>Cdip_DM

TRTLPKCARCKNHSVIAPLRGHKRYCKHRLCECDKCVLVAERQRVMARQTALRRAQAQDELLIRQ

>Cdip_DM2

TRTPPRCARCRNHSIKVPLRGHKRYCVYRSCKCPKCKLTAERQKVMAQQTALRRAQTQDELFGRQ

>Rpro_Dsx

ARTPPNCARCRNHSKTEPLKGHKRFCKYRTCTCKKCHLTVERQREMAKQTALRRELAQDEARARA

>Hvit_DM

SRTPPNCARCRNHEVILPLKGHKRYCKFMDCKCEKCELTAERQKVMARQTAIRRAEEQDRQRLKS

>Caqu_Dsx

NSRTPKCARCRNHKLNIAVKGHKRYCRYRDCMCEKCRLTAERQRVMALQVALRRAQVRQAAFNSC

>Afra_Dsx3

RTRVPTCARCRNHGKITKLRGHKRYCQFRACSCKLCVLTVDKQRVMAAQVANRRALKQDEENGVF

>Afra_Dsx4

KMRMPTCARCRNHGQVVKLRGHKRYCSFRHCLCDRCALTSEKQRVMAAQVALRRAQKQDEENGIV

>Hdeu_Dsx

SARTPKCARCRNHKVNVPVKGHKRYCEYRYCECERCVLTAERQRVMALQDEARAAQLRHQAATSP

>Dcar_Dsx1

SQRHPTCALCKNHQTISTLKGHKRYCPWRQCMCELCYGTNKKRKINAEQVALRRAQAQDEELRKK

>Dmag_Dsx1

SQRHPTCALCKNHQTISTLKGHKRYCPWRQCMCELCYGTNKKRKINAEQVALRRAQAQDEELRKK

>Dgal_Dsx1

TQRHPTCALCKNHQTISTLKGHKRYCPWRQCMCELCYGTNKKRKINAEQVALRRAQAQDEELRKK

>Dpul_Dsx1

TQRHPTCALCKNHQTISTLKGHKRYCPWRQCMCELCYGTNKKRKINAEQVALRRAQAQDEELRKK

>Mmac_Dsx

NQRHPTCALCKNHQTISTLKGHKRYCPWRTCLCELCYSTNKKRKINAEQVALRRAQAQDEELRKK

>Dcar_Dsx2

SCRNPTCALCKNHGINSPLKGHKRYCPFGRCSCDLCRVTRKKQKINASQVASRRAQQQDRELGID

>Dmag_Dsx2

SCRNPTCALCKNHGINSPLKGHKRYCPFGRCSCDLCRVTRKKQKINASQVASRRAQQQDRELGID

>Dgal_Dsx2

SCRNPTCALCKNHGINSPLKGHKRYCPFGRCSCDLCRVTRKKQKINASQVATRRAQQQDRELGID

>Dpul_Dsx2

SCRNPTCALCKNHGINSPLKGHKRYCPFGRCSCDLCRVTRKKQKINASQVATRRAQQQDRELGID

**Supplementary Sequence 3**

>Dmel_DsxF

PLMYVILKDADANIEEASRRIEEGQYV-------VNE-----------------------

-------------------YSRQHNL------NIYDGGELR---------------NTTR

QCG---------------------------------------------------------

------------------------------------------------------------

------------------------------------------------------

>Aobl_DsxF

PLMYVILKDAGADIEEASRRIEEGQHV-------VNE-----------------------

-------------------YSRQHNL------NIYDGGELR---------------STTR

QCG---------------------------------------------------------

------------------------------------------------------------

------------------------------------------------------

>Bdor_DsxF

PLMYVILKDAGADIEEASRRIEEGQHV-------VNE-----------------------

-------------------YSRQHNL------NIYDGGELR---------------STTR

QCG---------------------------------------------------------

------------------------------------------------------------

------------------------------------------------------

>Ccap_DsxF

PLMYVILKDAGADIEEASRRIEEGQHV-------VNE-----------------------

-------------------YSRQHNL------NIFDGGELR---------------STTR

QCG---------------------------------------------------------

------------------------------------------------------------

------------------------------------------------------

>Lcup_DsxF

PLMYVILKDAGVDIDEASKRIEEGQHV-------VNE-----------------------

-------------------YSRQHNL------NIYDGCELR---------------CATR

QCG---------------------------------------------------------

------------------------------------------------------------

------------------------------------------------------

>Agam_DsxF

PLMYVILKSADGDVQKAHQRIDEGQAV-------VNE-----------------------

-------------------YSRLHNL------NMFDGVELR---------------NTTR

QSG---------------------------------------------------------

------------------------------------------------------------

------------------------------------------------------

>Cqui_DsxF

PLMYVILKGADGDVQTAHRRIDEGQAV-------VNE-----------------------

-------------------YSRLHNL------NMFDGVELR---------------STQR

QSG---------------------------------------------------------

------------------------------------------------------------

------------------------------------------------------

>Aaeg_DsxF

PLMYVILKGADGDVNKARQRIDEGQAV-------VNE-----------------------

-------------------YSRLHNL------NMFDGVELR---------------STTR

QSG---------------------------------------------------------

------------------------------------------------------------

------------------------------------------------------

>Mdom_DsxF

PLMYVILKDAGVDIDEASKRIEEGQHV-------VNE-----------------------

-------------------YSRQHNL------NIYDGCELR---------------CATR

QCG---------------------------------------------------------

------------------------------------------------------------

------------------------------------------------------

>Bmor_DsxF

PLVLVIMNYARSDLDEASRKIYEGKMI-------VDE-----------------------

-------------------YARKHNL------NVFDGLELR---------------NSTR

QKML--EINNISGVL-----------------------------------SSSMK-----

----------------------------------LFCE----------------------

------------------------------------------------------

>Amyl_DsxF

PLVLVILNYAGSDLEEASRKIDEGKMI-------INE-----------------------

-------------------YARKHNL------NVFDGLELR---------------NSTR

QKML--EINNISGVL-----------------------------------SSSMK-----

----------------------------------LFCE----------------------

------------------------------------------------------

>Osca_DsxF

PLVLVILNYAGSDLDEASRKIDEGKMI-------INE-----------------------

-------------------YARKHNL------NIFDGLELR---------------NSTR

QKMLQSEINNISGVL-----------------------------------SSSMK-----

----------------------------------LFCE----------------------

------------------------------------------------------

>Ekue_DsxF

PLVLVILNYAGSDLDEASRKIDEGKMI-------INE-----------------------

-------------------YARKHNL------NIFDGLELR---------------NSTR

QKMLRSEINNISGVL-----------------------------------SSSIK-----

----------------------------------LFCE----------------------

------------------------------------------------------

>Tvar_DsxF

PLVLVIMNYARSDLDEASRKIYEGKMI-------VDE-----------------------

-------------------YARKHNL------NVFDGLELR---------------NSTR

QYGL--------------------------------------------------------

------------------------------------------------------------

------------------------------------------------------

>Harm_DsxF

PLVLVILNYAGSDLDEATRKIDEGKMI-------INE-----------------------

-------------------YARKHNL------NVFDGLELR---------------NSTR

HAVF--CVDACCAMLCCERLRTLLWLPLDPRPLGGPPMAVVRAVAVLATGATSAQGLVLH

V------LFAEVRAGAGVRAAPAAAAPHHAARVAAACALVAMRTCAASGGARAPGPRFDF

HVDVSGGRRRQLLVSSRHCGDSVYIPGHLVLDLLSYTHTKMYLMLCCCKTIFAH

>Pxut_DsxF

PLVLVILNYAGSDLDEASRKIDEGKLI-------VNE-----------------------

-------------------YARKHNL------NIFDGLELR---------------NSTR

QYGL--------------------------------------------------------

------------------------------------------------------------

------------------------------------------------------

>Msex_DsxF

PLVLVILNYAGSDLDEASRKINEGKTI-------INE-----------------------

-------------------YARKHNL------NVFDGTEFR---------------DSTR

QYGL--------------------------------------------------------

------------------------------------------------------------

------------------------------------------------------

>Pxyl_DsxF

PLVLVILNYAGSDLEEASRKIDEGKIM-------IDD-----------------------

-------------------YARKHNL------NIFAGLELR---------------NSTR

QYGL--------------------------------------------------------

------------------------------------------------------------

------------------------------------------------------

>Prap_DsxF

PLVLVILNYAGNDLEEASRKIDEGKMI-------INE-----------------------

-------------------YARKHNL------NIFDGLELR---------------NSTR

QKML--SEINISGVF-----------------------------------SSSMK-----

----------------------------------LFCE----------------------

------------------------------------------------------

>Tcas_DsxF

PLMYAILKDARADLEEASRRIDEGKRV-------VNE-----------------------

-------------------YSRLHNL------NMYDGVELR---------------NSTH

NKNQ--DRRSFSS-------------------------------------NPSFKSVNLQ

F----------------------------------KCNVLI-------------------

------------------------------------------------------

>Gcor_DsxF

PLMYAILKDARADLEEASRRIDEGKRA-------VNE-----------------------

-------------------YSRMHNL------NMYDGVELR---------------NSTH

NKNQ--ERRING--------------------------------------NPPFKSVNVQ

F----------------------------------KCNVLI-------------------

------------------------------------------------------

>Tdic_DsxF

PLLYAIMKLA-PNLEEASKRIDEGKQM-------VYE-----------------------

-------------------YSQRYNL------NMYDGGELR---------------KSTH

KENR--EKNNFPK--------------------------------------ATLTSVNLQ

L----------------------------------KCNLLMPY-----------------

------------------------------------------------------

>Cmet_DsxF

PLMYAILKDA-PDLEEASKRIDEGKRV-------VYE-----------------------

-------------------YSKMNNI------NMFDGGLLR---------------NSTH

EKNQ--QRRNFSA-------------------------------------ATTFKSVNLQ

L----------------------------------KCNLLI-------------------

------------------------------------------------------

>Otau_DsxF

PLIYAIVKDT-PDLEEASKRIDEGQRA-------VKE-----------------------

-------------------YSIINNL------NMYDGGELR---------------YPTH

KRAQ--ERRSSGT-------------------------------------FKPIGGVNLQ

V----------------------------------KRNLHVK------------------

------------------------------------------------------

>Dpon_DsxF

PLMYAILKDARADLDEASRRIDEGKQV-------VNE-----------------------

-------------------YSRTHNL------NMYDGVKLR---------------PSSP

AYLD--FRTKAAAYL---------YAPYN---------------NCYSSGATVFPPLYLS

SFYPASSLL------------PPTMPPN-------LCS-------------RPPSPR---

------------------------SPHNLTMPLRLHS--------------IAG

>Swra_DsxF

PLMYVILKDAQADLEEASRRIDEGKRV-------VNE-----------------------

-------------------YSRIHNL------NMYDGIELR---------------NSTR

QYG---------------------------------------------------------

------------------------------------------------------------

------------------------------------------------------

>Eaur_DsxF

PLMYVILKDAQADIEEASRRIDEGKRA-------VNE-----------------------

-------------------YSRIHNL------NMYDGIELR---------------NSTR

QYG---------------------------------------------------------

------------------------------------------------------------

------------------------------------------------------

>Hred_DsxF

TLIYTVLRLVNGDEEYAAKTIREGKRV-------VNE-----------------------

-------------------YSLTHNL------NMYDGVELR---------------NSTR

HYG---------------------------------------------------------

------------------------------------------------------------

------------------------------------------------------

>Mlim_DsxF

PLMYAILKDARRDLEEAARRIDEGKRV-------VNE-----------------------

-------------------YSRMHNL------NMYDGVELR---------------NSTH

NNKLQ-ERRNFN--------------------------------------NSTFKSVNLQ

F----------------------------------KCNVLI-------------------

------------------------------------------------------

>Aros_DsxF

PLMLVILKDAQADLEEASRRIAEADFT-------AAARSEIIYFIAPFWPVDNTEKNDEP

CSSAICRLLHFSRDECKI-FCMIFSY------FLYTATAIRRIAVICAAESIKFHFFSIR

RYRM--SRSNVQYRIKH-----------------------------R---LEDYKSINED

LY----DL----------------------------------------------------

----------------------------------------------------SG

>Nlec_DsxF

PLMFVILKDARADLEEASRRITEDATT-------RKLLSL--------------------

CM-------------TTISYSLMFINRC----KVFVAITV----------------FYSH

Q----------------------------------------------------IELVN--

------------------------------------------------------------

------------------------------------------------------

>Xalp_DsxF

PLMYVILKDARADFEEASRRIAEAVDF-------FGL-----------------------

----------------KFTFSTIIT-----------------------------------

------------------------------------------------------------

------------------------------------------------------------

------------------------------------------------------

>Ccin_DsxF

PLMYVILKDAKADLEEATRRIAEAVDV-------FHL-----------------------

----------------KLTYSTMII-----------------------------------

------------------------------------------------------------

------------------------------------------------------------

------------------------------------------------------

>Dall_DsxF

PLMYVILKDAKADLDEARRRIEEAVDT-------FRV-----------------------

----------------NLTFSTMVI-----------------------------------

------------------------------------------------------------

------------------------------------------------------------

------------------------------------------------------

>Atab_DsxF

PLMYVILKDAKADLDEARRRIAEAVDT-------FRD-----------------------

----------------ELTFDTMVI-----------------------------------

------------------------------------------------------------

------------------------------------------------------------

------------------------------------------------------

>Nvit_DsxF

TLMYVILKDSRADVEVAMRRITQAKNV-------WQP-----------------------

----------------EL-YSRIISV----------------------------------

------------------------------------------------------------

------------------------------------------------------------

------------------------------------------------------

>Tdub_DsxF

TLMYVILKDSRADVEVAMRRITQAKNV-------WQP-----------------------

----------------EV-YSRIISI----------------------------------

------------------------------------------------------------

------------------------------------------------------------

------------------------------------------------------

>Fvar_DsxF

LLMYINLKYAGANPEEVVRRMVDVSIV-------FCP-----------------------

---------------KNFTWNSILRKILSFIVNLSPA-----------------------

------------------------------------------------------------

------------------------------------------------------------

------------------------------------------------------

>Amel_DsxF

LLMYINLKYAGANPEEVVRRMVDALII-------FCS-----------------------

---------------KNFIWNSILNKIVSFI-NLLPT-----------------------

------------------------------------------------------------

------------------------------------------------------------

------------------------------------------------------

>Phum_DsxF

PLAYVVFKIAGGDYEVATKMILEGYLTYPFTDCETQE-----------------------

------------NEEMDL-YCRFF------------------------------------

------------------------------------------------------------

------------------------------------------------------------

------------------------------------------------------

>Gewi_DsxF

PLAYVVFKIAGGDYEVATKMILEGYLSYPFTDSETQE-----------------------

------------NDEMDL-YCRFF------------------------------------

------------------------------------------------------------

------------------------------------------------------------

------------------------------------------------------

>Rpro_DsxF

PLVYIILKFTKS-YDEALKIIAERMQVKK-THFLIKK-----------------------

-------------------NIRIDNL------NLNDNFIL--------------------

------------------------------------------------------------

----------------------------------IRC-----------------------

------------------------------------------------------

>Xmet_DsxF

PLLHIILNKITPNIKEAYSLILAGRPQLP-NLYKLTE-----------------------

-------------------WNSIS------------------------------------

------------------------------------------------------------

------------------------------------------------------------

------------------------------------------------------

>Nlug_DsxF

TLLYVILNIVCPNVDEAYNRILNGSMN------SVVERTV-------------------P

C-----------------------------------------------------------

------------------------------------------------------------

----------------------------------IPC------C----------------

------------------------------------------------------

>Cpun_DsxF

PLIYVVLQVSQSDVTMASNRILKGKTLHV---FICYELII--------------------

-------------------YQTIYTIFI----LVYS------------------------

------------------------------------------------------------

------------------------------------------------------------

------------------------------------------------------

>Bger_DsxF1

PLIYVVLQLSHSDVKEAATRIIKGQID------ILAE-----------------------

-------------------YKFRLTLRS----RL--------------------------

------------------------------------------------------------

------------------------------------------------------------

------------------------------------------------------

>Bger_DsxF2

PLIYVVLQLSHSDVKEAATRIIKGQML------VLCELAK--------------------

-------------------YK---------------------------------------

------------------------------------------------------------

------------------------------------------------------------

------------------------------------------------------

>Isen_DsxF

PLIYAILQNARCDVKEATHQIQDGGYR-------ISRLPL--------------------

-------------------VAVK-------------------------------------

------------------------------------------------------------

------------------------------------------------------------

------------------------------------------------------

>Tdom_DsxF

PLIYVVLKDARSDVKEASNRIMEGVYQ-------CMNQGI--------------------

-------------------IAPIG------------------------------------

------------------------------------------------------------

------------------------------------------------------------

------------------------------------------------------

**Supplementary Sequence 3**

>Aparaglossata_AS

PLMYVILKDARADLEEASRRIDEGKRVVNEYSRMHNLNMYDGVELRNSTRQYG

>Holometabola_AS

PLMYVILKDARADLEEASRRIDEGKRVVNEKLYSRMHNL

>Eumetabola_AS

PLMYVILKDARADLEEASKRIMEGKRVVVNERTIYSRMH

>Neoptera_AS

PLIYVVLKDARSDVKEASNRIMEGKYLICCEQAIYAPIH

>Pterygota_AS

PLIYVVLKDARSDVKEASNRIMEGVYQCMNQGIIAPIG

**Supplementary references**

Beutel RG, Yavorskaya MI, Mashimo Y, Fukui M, Meusemann K. 2017. The phylogeny of Hexapoda (Arthropoda) and the evolution of megadiversity. *Proc Arthropod Embryol Soc Jap.* 51:1–15.

Boudinot BE. 2018. A general theory of genital homologies for the Hexapoda (Pancrustacea) derived from skeletomuscular correspondences, with emphasis on the Endopterygota. *Arthropod Struct Dev.* 47(6):563–613.

Emeljanov AF. 2014. The evolutionary role and fate of the primary ovipositor in insects. *Entomol Rev.* 94(3):367–396.

Gustafson GT, Baca SM, Alexander AM, Short AEZ. 2020. Phylogenomic analysis of the beetle suborder Adephaga with comparison of tailored and generalized ultraconserved element probe performance. *Syst Entomol.* 45(3):552–570.

Kawahara AY, Plotkin D, Espeland M, Meusemann K, Toussaint EFA, Donath A, Gimnich F, Frandsen PB, Zwick A, dos Reis M, et al. 2019. Phylogenomics reveals the evolutionary timing and pattern of butterflies and moths. *Proc Natl Acad Sci USA.* 116(45):22657–22663.

Kristensen NP. 1975. The phylogeny of hexapod “orders”. A critical review of recent accounts. *J Zool Syst Evol Res.* 13(1):1–44.

Li H, Leavengood JM, Chapman EG, Burkhardt D, Song F, Jiang P, Liu J, Zhou X, Cai W. 2017. Mitochondrial phylogenomics of Hemiptera reveals adaptive innovations driving the diversification of true bugs. *Proc R Soc B.* 284:20171223.

Matsuda R.1976. *Morphology and Evolution of the Insect Abdomen: With Special Reference to Developmental Patterns and their Bearings upon Systematics.* Oxford: Pergamon Press.

Misof B, Liu S, Meusemann K, Peters RS, Donath A, Mayer C, Frandsen PB, Ware J, Flouri T, Beutel RG, et al. 2014. Phylogenomics resolves the timing and pattern of insect evolution. *Science* 346(6210):763–767.

Peters RS, Krogmann L, Mayer C, Donath A, Gunkel S, Meusemann K, Kozlov A, Podsiadlowski L, Petersen M, Lanfear R, et al. 2017. Evolutionary History of the Hymenoptera. *Curr Biol.* 27(7):1013–1018.

Snodgrass RE. 1957. A revised interpretation of the external reproductive organs of male insects. *Smithson Misc Collect.* 135:1–60.

Wiegmann BM, Trautwein MD, Winkler IS, Barr NB, Kim JW, Lambkin C, Bertone MA, Cassel BL, Bayless KM, Heimberg AM, et al. 2011. Episodic radiations in the fly tree of life. *Proc Natl Acad Sci USA.* 108(14):5690–5695.

Zhang SQ, Che LH, Li Y, Liang D, Pang H, Ślipiński A, Zhang P. 2018. Evolutionary history of Coleoptera revealed by extensive sampling of genes and species. *Nat Communu.* 9(1):1–11.
